# Molecular architecture of heterochromatin at the nuclear periphery of primary human cells

**DOI:** 10.1038/s41467-026-75087-5

**Published:** 2026-07-03

**Authors:** Jan Philipp Kreysing, Sergio Cruz-León, Johannes Betz, Carlotta Penzo, Tomáš Majtner, Markus Schreiber, Beata Turoňová, Marina Lusic, Gerhard Hummer, Martin Beck

**Affiliations:** 1 Department of Molecular Sociology, Max Planck Institute of Biophysics, Max-von-Laue-Straße 3, Frankfurt am Main, Germany; 2 IMPRS on Cellular Biophysics, Max Planck Institute of Biophysics, Max-von-Laue-Straße 3, Frankfurt am Main, Germany; 3 Department of Theoretical Biophysics, Max Planck Institute of Biophysics, Max-von-Laue-Straße 3, Frankfurt am Main, Germany; 4 Department of Infectious Diseases, Integrative Virology, Heidelberg University, Heidelberg, Germany; 5 Institute of Biophysics, Goethe University Frankfurt, Frankfurt am Main, Germany; 6 Institute of Biochemistry, Goethe University Frankfurt, Frankfurt am Main, Germany

**Keywords:** Cryoelectron tomography, Computational biophysics

## Abstract

In eukaryotes, meters of DNA are packaged into micrometer scale nuclei. Nucleosomes, as the major organizational unit, have been extensively studied in vitro, yet the elaborate 3D structure of chromatin inside cells and its distinct oligo-nucleosome arrangements remain poorly resolved. Here, we combine cryo-electron tomography with template matching, subtomogram averaging and molecular simulations to visualize nucleosomes and chromatin structure inside human cells. We confidently assign individual nucleosomes and report their in-situ structure at secondary structure resolution. By predicting the paths of linker DNA, we identify oligo-nucleosome arrangements and uncover higher-order chromatin structures in situ, including a 37-nm wide, elongated but non-fibrous arrangement. In situ structural biology thus reveals the molecular chromatin organization inside cells and sets the stage for 3D genomics.

## Abstract (web summary)

The 3D organisation of chromatin inside a human cell is still not fully resolved. Here the authors reveal that in situ cryo-ET resolves individual nucleosomes and predicts DNA linkers, thus showing the molecular architecture of heterochromatin inside of resting T cells.

## Introduction

The genetic material within each human cell consists of ~two meters of DNA that needs to be packaged into a nucleus that is six orders of magnitude smaller in diameter. This packaging challenge is compounded by the requirement that the compacted genome must remain accessible for transcription, replication and remodeling in a controlled, efficient, and temporally precise manner. One mechanism by which cells meet this challenge is by wrapping DNA around histone proteins. The resulting protein-DNA complexes form nucleosomes, often depicted as”beads on a string”, which in turn fold into stable, higher-order structures, to finally form the complex three-dimensional arrangement known as chromatin. The hierarchical architecture of chromatin has been studied extensively. Light microscopy imaging and advanced sequencing techniques have revealed chromosome organization, chromatin compartments or topologically associating domains (TADs), while structural biology techniques have been used to analyze the molecular details of how DNA is wrapped around individual reconstituted nucleosomes^1^. However, how native chromatin is organized at the nucleosome level in living cells remains the subject of active research.

Canonical nucleosomes are formed by a C2-symmetric octamer of two copies of each of the core histones H2A, H2B, H3 and H4. 147 base pairs (bp) of DNA are wrapped in a left-handed superhelical turn around this octamer, and flanked by DNA linkers of variable length that project towards the next nucleosome^2^. A single H1 linker histone can bind to the DNA adjacent to the core nucleosome superhelix, thereby disrupting the nucleosome’s 2-fold symmetry^3^. The core nucleosome with H1 and its two DNA linkers, referred to as a chromatosome, is thought to be the building block of higher-order chromatin structures. H1 variants can bind to the two DNA linkers, the DNA wound around the histone octamer, or all three DNA structures simultaneously, resulting in differential stabilization and either on-dyad or off-dyad arrangements^4,5^.

Local chromatin folding is known to be determined by nucleosome organization^5^. Several models have been proposed for the arrangement of nucleosomes into a 30-nm chromatin fiber. In the zig-zag model, alternating nucleosomes interact, whereas the solenoid model implies interactions of neighboring nucleosomes^5^. Indeed, arrangements of multiple nucleosomes on DNA in vitro are characterized by a zig-zag arrangement, whereby nucleosomes can stack with each other^6–9^, and the DNA linkers restrain the orientation of consecutive nucleosomes^10^. In addition, a flat ladder-like structure and twisted arrangements have been described^5^. However, recent studies have suggested that chromatin can be highly dynamic and variable inside cells questioning the physiological relevance of a static 30-nm fiber^11^. Chromatin folding and its compaction behavior is influenced by H1 binding in on-dyad or off-dyad position^12^. Reduced DNA linker lengths and consequently shorter nucleosome repeat lengths (NRLs) sterically hinder H1 binding and result in less regular arrays, which in turn are associated with active chromatin^6,13^. These molecular details have been derived from extensive in vitro structural analyses of reconstituted or purified histones, often using artificial, so-called 601 nucleosome positioning sequence (NPS) repeats^14^. To which extent these studies reliably recapitulate the architecture of native chromatin in situ, which is not only dependent on NRLs, but also affected by phase separation, ionic strength and various other associated proteins beyond linker H1^15,16^, remains largely unknown.

Several recent studies have investigated nucleosome arrangements in different cell types in situ using cryo-electron tomography (cryo-ET)^17–25^. An overall relatively heterogenous chromatin arrangement was reported^20^. Human cells contained a fibrous chromatin structure lining the nuclear envelope^18,21^, with exclusion zones beneath nuclear pores^21^. However, the respective in situ structures of nucleosomes were not resolved to high resolution^18–21^, and the topology of neighboring nucleosomes was not assessed with respect to the above-discussed structural features that are known to constitute chromatin architecture in vitro. Studies in Drosophila embryos^17^ and frog erythrocyte nuclei^22^ have pointed to irregular zig-zag chromatin folding patterns.

Here, we determine the structure of nucleosomes and their arrangements in situ by combining cryo-ET with template matching, subtomogram averaging (STA) and molecular simulations. We use resting T cells for our analysis because heterochromatin is prominently enriched in proximity to the nuclear envelope. We resolve the chromatosome to secondary structure in situ; we predict DNA linkers between individual chromatosomes up to kilobase pair (kbp) genomic fragments; we identify key molecular determinants that induce order or disorder in oligo-nucleosome arrangements; and we explain how nucleosome stacking as well as orientation and length of the DNA linkers contribute to the ~37 nm broad, elongated, but non-fibrous arrangement we observe in situ. Our study highlights the potential of in situ structural biology to examine chromatin structure at the oligo-nucleosome scale directly within the dense environment of the cell nucleus. As such, it opens the door for 3D genomics and the study of spatial genome regulation within the nuclear space inside cells.

## Results

**Fig. 1:**
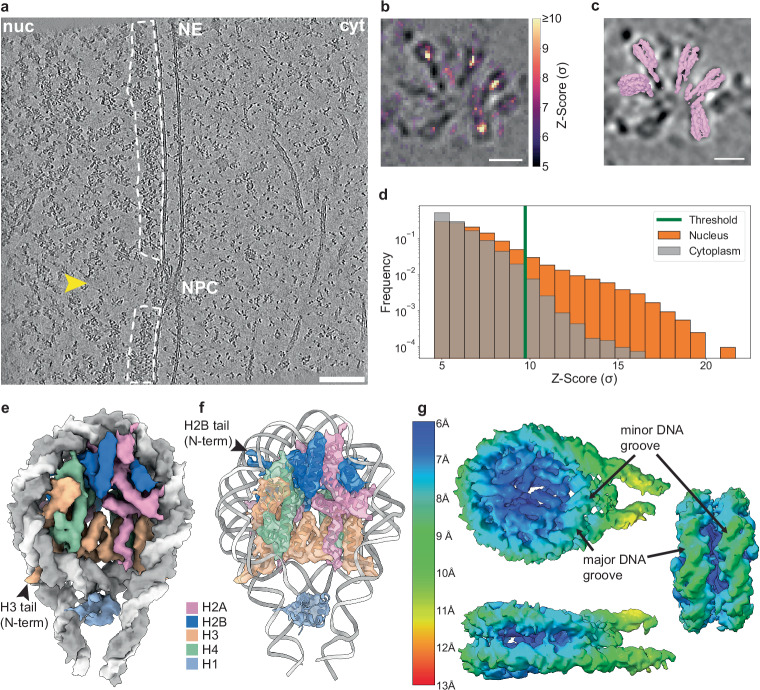
In situ structural analysis identifies nucleosomes inside of cells and resolves their secondary structure. **a** Slice through a cryoCARE-denoised^55^ tomogram acquired at the nuclear periphery of resting T cells. Chromatin-free space is indicated with a yellow arrow, a dense layer of chromatin underlying the nuclear envelope is framed white. **b** A local feature showing multiple nucleosomes. Superimposed is the cross-correlation function obtained by template matching. **c** The same local feature but with the identified nucleosomes shown as isosurfaces. **d** Histogram of Z-scores for the cytosolic and nucleoplasmic region for an exemplary tomogram. **e**, **f** STA map of the human chromatosome, color-coded by DNA strands (white and gray) and histone proteins H2A (pink), H2B (blue), H3 (amber), H4 (green) and H1 (light blue). The atomic model of a canonical chromatosome (PDB: 7DBP^30^) was fitted into our map. The partially resolved N-terminal tails of the H2B and H3 histones are highlighted with arrows. **g** Color-coded local resolution STA map of the human chromatosome (from high resolution in blue to low resolution in red, in Å). The major and minor DNA groove are highlighted with arrows. Scale bars 100 nm in a and 10 nm in (**b**, **c**). Source data are provided as a Source Data file.

To study nucleosome arrangements in their native state, we vitrified resting T cells from human donors and subjected them to specimen thinning by focused ion beam (FIB) milling. 14 tilt series were acquired at random positions at the nuclear periphery (Supplementary Fig. 1), with a field of view ranging up to 400 nm into the nucleus. To confirm that this region is enriched for heterochromatin, we quantified the localization of suitable histone marks (Supplementary Fig. 2). Tilt series were further processed with a projection interpolation algorithm, cryoTIGER^26^, to improve angular sampling reconstructed in novaCTF^27^ and template matched using GAPSTOP^TM^^28,29^ with a nucleosome structure (Fig. 1a–c) (see “Methods” and Supplementary Fig. 3 for details). The resulting cross-correlation peaks (Fig. 1b) visually coincided with the particles that resembled nucleosomes (Fig. 1c). These were considerably more frequent in the nucleoplasm as compared to the cytosol (Supplementary Fig. 4), in line with the expected localization of chromatin. We therefore set the threshold for peak extraction at the 99^th^ percentile of cytoplasmic peaks (Fig. 1d, see “Methods” for details). We observed an about 50 nm thick layer densely packed with nucleosomes underneath the inner nuclear membrane (Fig. 1a), which is consistent with recent findings on lamin-nucleosome interactions in Murine Embryonic Fibroblasts^21^.

STA (performed with non-interpolated data) confirmed that the extracted peaks primarily account for nucleosomes. We obtained an in situ structure of the chromatosome from resting T cells locally resolved to 6.0 Å in the nucleosome core, 8–10 Å in the DNA (6.8 Å overall) with imposed C2 symmetry (Fig. 1e-g). It closely resembles previous in vitro structures^30^ of canonical chromatosomes with H1 bound and two DNA linker arms. The STA map clearly showed the secondary structure features of all core histones, the major and minor groove of the wrapped DNA emerging into the DNA linkers, and the histone tails of histone H2B and H3 projecting outwards through the DNA. Without imposed 2-fold symmetry the resolution was slightly reduced (Supplementary Figs. 3, 5, and Supplementary Table 1), but the asymmetrically associated histone H1 was more clearly resolved. In contrast, efforts using H1-free nucleosomes as reference for extensive classification did not result in decently resolved H1-free nucleosome structures (Supplementary Fig. 6). Instead, we identified nucleosomes in which H1 was shifted to either DNA linker (Supplementary Fig. 7). These data suggest that the H1 histone influences the arrangement of nucleosomes relative to one another and is particularly abundant at the nuclear periphery in resting T cells. In addition, to the best of our knowledge, our nucleosome structure from resting T cells represents the smallest non-oligomeric structure resolved to secondary structure resolution by STA in situ to date, with a molecular weight of about 250 kDa.

**Fig. 2:**
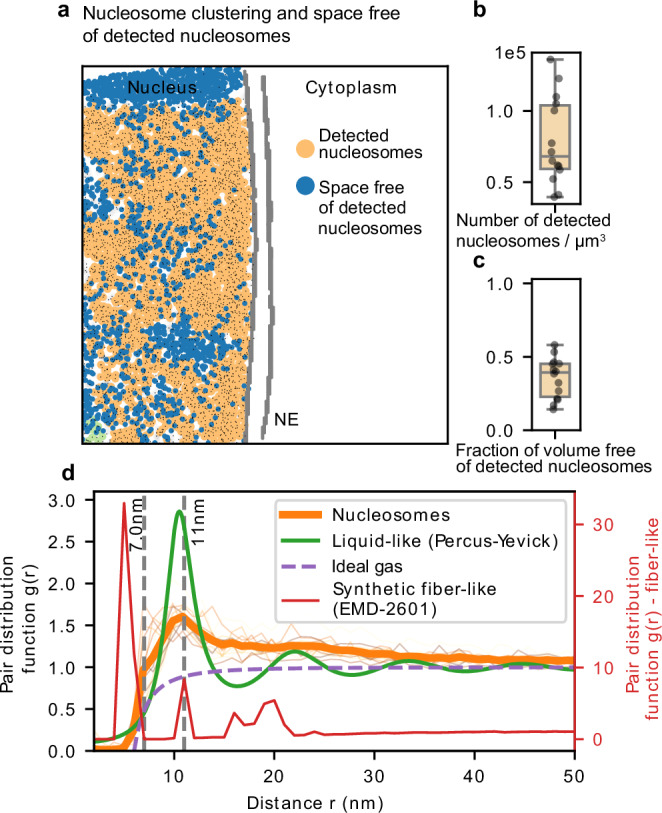
Spatial properties of detected nucleosomes at nuclear periphery of T cells. **a** Hierarchical density-based clustering (orange and green clusters) and space free of detected nucleosomes visualized from the same exemplifying tomogram as in Fig. 1a. **b** Overall number of detected nucleosomes per unit volume with each tomogram displayed as single dot (*n* = 14 tomograms). **c** Fraction of nuclear volume free of detected nucleosomes with each tomogram displayed as single dot (*n* = 14 tomograms). **d** Pair distribution function g(r) of the detected nucleosomes. Reference curves for the ideal gas and liquid-like distributions^84^, and a synthetic tomogram made of 30-nm-fiber segments (EMD-2601^7^) are included for comparison. Gray dashed lines are the peaks of nucleosomes g(r) function. For **b**, **c** box plots show the median as the center line, the 25th and 75th percentiles as the lower and upper bounds of the box, and the minimum and maximum values as whiskers. Individual data points are shown as black dots. Source data are provided as a Source Data file.

We next used the set of detected nucleosomes to determine spatial properties (Fig. 2). Clustering analysis revealed an overall densely packed nucleoplasm with some occasional nucleosome-free patches, e.g., underneath nuclear pores (Fig. 1a). We also quantified how much nuclear volume was occupied by the detected nucleosomes as compared to free space (Fig. 2a, c), and found that less than half of the volume at the nuclear periphery is free of detected nucleosomes, using a 5 nm probe volume. Finally, we quantified native chromatin in terms of the nucleosome pair distribution function $g(r)$ (Fig. 2d). In terms of $g(r)$, we find chromatin to be intermediate between a liquid and a gas with two short-range peaks at 7 and 11 nm center-to-center distance likely representing stacked (Supplementary Fig. 8) and edge-contacting nucleosomes, and a gradual decay beyond 20 nm indicating a weak tendency to condense (Fig. 2d). The relatively featureless curve provides no evidence for long-range order in chromatin, arguing against fibrous chromatin structures (theoretical curve for 30-nm fiber shown for comparison in Fig. 2d), even if one accounts for false negatives in nucleosome detection.

**Fig. 3:**
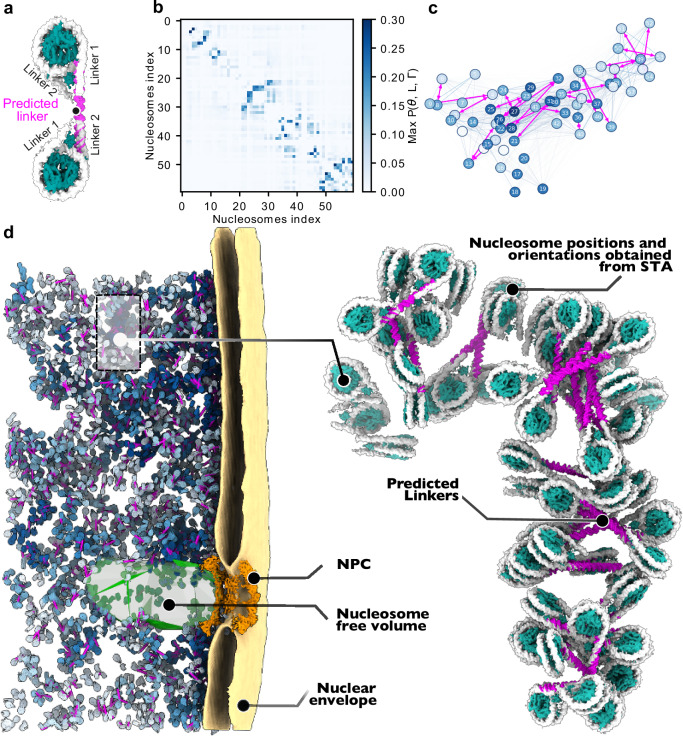
Cryo-ET and physics-based model allow kbp-long chromatin to be visualized. **a**–**c** A physics-based model of nucleosome pairs is used to add DNA linkers (magenta) to in situ nucleosome DNA (white) (**a**). All four distinct possibilities to connect two nucleosomes with two linkers each are scored, resulting in connectivity matrix for all nucleosomes within a given cluster (see Methods and Supplementary Fig. 9 for an example) (**b**). The connectivity matrix from b is represented as a graph, with nucleosomes as nodes connections as edges (**c**). Nodes are projected on the x-y plane and colored according to the z-coordinate. Edge thickness and color are proportional to the connection probability. Assigned linkers are shown as solid arrows between nodes. **d** Exemplifying, segmented and isosurface-rendered tomogram (corresponding to Fig. 1a) showing the densely interconnected chromatin structure within the nucleus. Nucleosomes are colored according to the length of the traced chromatin chain, from short (white-light blue) to long (dark blue); predicted linkers are shown in magenta. The nuclear envelope membrane was segmented with MemBrain-seg^85^. The zoom-in is the 3D representation of 60 nucleosomes in (**b**, **c**). The nucleosomes were placed back into the tomographic volume using the ChimeraX^61^ plugin ArtiaX^86^. Source data are provided as a Source Data file.

To analyze oligo-nucleosome arrangements in more detail, we connected consecutive nucleosomes by predicting the DNA linkers. We first used a distance-based clustering as implemented in cryoCAT^31^ to identify the candidate nucleosomes to which they could be connected by a DNA linker. For this, we select neighbors that have at least one of their linker arms (linker 1 and linker 2 in Supplementary Fig. 9b) within 20 nm of at least one of the two linker arms of the other nucleosome. Within a given local cluster defined by this distance criterion, the positions and orientations allowed us to connect candidate nucleosome pairs by using a simple physics-based DNA model of the bending energy *U*_bend_ of the DNA linker (Fig. 3a and Supplementary Fig. 9a). The bending energy *U*_bend_ is calculated based on the linker length (L), the bending angle (θ), and the configuration of the DNA linker between a pair of nucleosomes that is connected (where Γ denotes the specific linker connection in Supplementary Fig. 9b) (see “Methods” for details) as follows:

1 $${U}_{{{{\rm{bend}}}}}\left({{{\rm{\theta }}}},L\right)=\frac{2{l}_{p}}{L}{\left(\frac{{{{\rm{\theta }}}}}{2}\right)}^{2}{k}_{B}T$$

Here ${l}_{p}\approx 50$ ${{{\rm{nm}}}}$ is the B-DNA persistence length at physiological conditions, ${k}_{B}T$ is the thermal energy and $L=\theta r$ is the length of the arc of a circle sector of radius $r$ and central angle θ (in radians). The radius $r$ is related to the Euclidian distance $D$ between the ends of the linker arms on the two nucleosomes (Supplementary Fig. 9a) through

2 $${r}=\frac{D}{{2} \, {{\rm{sin}}}\left(\frac{\theta}{2}\right)}$$

With the energy function in Eq. 1, we estimated the probability that the DNA linker arms of nearby nucleosomes are connected by a DNA linker as

3 $$P\left(L,\theta \right)={e}^{-\frac{L}{{L}_{0}}}{e}^{-\frac{{U}_{{{{\rm{bend}}}}}\left({{{\rm{\theta }}}},L\right)}{{k}_{B}T}}$$

where ${L}_{0}$ is a reference linker length, which we set to ${L}_{0}=15$ nm as an expected typical linker length. The resulting probability function thus penalizes very long but straight as well as very short but strongly bent linkers. Both ${L}_{0}$ and ${l}_{p}$ are parameters of the algorithm that can be adjusted according to species, tissue, cell type, cell state, and genome location, if needed.

In a spatial cluster of $N$ nucleosomes, we estimated in this way the relative probabilities for each of the $2N(N-1)$ possible distinct connections to be formed, with four possible connections between the two arms of a nucleosome pair. The resulting connectivity “matrix” ${P}_{{ijk}}=P\left({L}_{{ijk}},{\theta }_{{ijk}}\right)$ of the size $4\times N \times N$ accounts for all possible connections in a probabilistic manner, where $i$ and $j$ index the $N$ nucleosomes of the cluster and $k$ indexes the four possible linker-arm connections between two nucleosomes. Connections are formed in a step-wise manner whereby the most likely connections are assigned in a “greedy” algorithm. This algorithm has three steps that are applied iteratively:

1. Identify the highest value in the current ${P}_{{ijk}}$ matrix, which gives a connection $(n,m,l)$ between nucleosomes $n$ and $m$ for linker connection $l$.
2. If the probability exceeds a predefined threshold, ${P}_{{nml}} > {P}_{\min }$, add $(n,m,l)$ to the list of connections; otherwise stop the search.
3. If $(n,m,l)$ has been added as new connection, update the connectivity matrix by setting ${P}_{{nml}}$ to zero; in addition, we set to zero all entries in the ${P}_{{ijk}}$ matrix that lead to an unfeasible solution with a closed form of the DNA (i.e., with the DNA emanating from one nucleosome arm and eventually returning to the other arm of the same nucleosome if traced along the list of connections) or to multiple connections to the same linker arm.
4. Return to step 1.

Results for the matrix ${P}_{{ijk}}=P\left({L}_{{ijk}},{\theta }_{{ijk}}\right)$ of relative connection probabilities between nucleosome arms are visually illustrated in Fig. 3b and Supplementary Fig. 9c. Shown is the ${P}_{{ij}}={\max }_{{{{\rm{k}}}}}{P}_{{ijk}}.$ We find that multiple nucleosomes can be confidently linked by choosing pairs connected with high probability. Similar probabilities for multiple possible pathways rarely arise, leaving the linker solution clear in the vast majority of assigned nucleosome pairs (Supplementary Fig. 10a). Therefore, a simple “greedy” algorithm sufficed here to connect nucleosomes, starting with the pair of nucleosome arms connected with the highest probability to the not-yet-connected pair with the next-highest probability and so on, always ensuring that no closed DNA loops are formed. The algorithm stops if the next connection falls below a preset threshold, here set to ${P}_{min }=0.1$. Tests of the algorithm with known in-vitro structures showed that our algorithm is robust to positional and orientational noise (Supplementary Fig. 12) and resilient to false negatives in the nucleosome detection (Supplementary Fig. 13). Performance remains strong under the uncertainties estimated by Relion 3.1^32^. Although most nucleosome connections are pairs (Supplementary Fig. 14), in some cases, up to kbp-long chromatin fragments become interconnected (Fig. 3c,d, and Supplementary Fig. 14), and ultimately break as the chain of nucleosomes reaches the edge of the FIB lamella.

To validate the outcome of the algorithm, i.e., the assigned nucleosome pairs, we subjected the predicted DNA linkers to STA. The averages clearly showed elongated density with the expected width underscoring that we had identified regions that contained DNA (Supplementary Fig. 10). We next grouped the subtomograms according to the predicted linker length, and subjected the individual classes to STA separately. We found that the length observed in the averages corresponds to the linker length predicted by our algorithm (Supplementary Fig. 10d). As the length of linker DNA increases, it can bend more and becomes less apparent in the averages, likely due to conformational variability (Supplementary Fig. 10c). Beyond ~70 bp, this flexibility blurs out linker densities in the STA. For further validation, we overlayed a selection of our predicted linkers on tomographic slices and confirmed the presence of electron optical density connecting the nucleosomes (Supplementary Fig. 11).

**Fig. 4:**
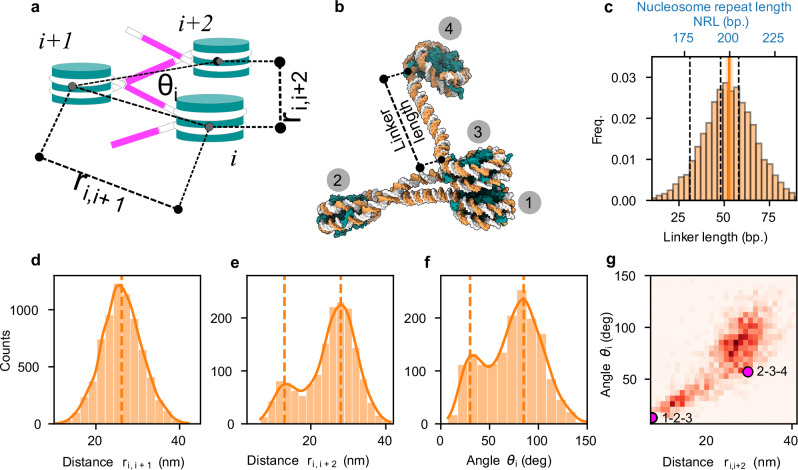
Molecular determinants of oligo-nucleosomes that define heterochromatin architecture in situ. Panels **a**, **b** depict the oligo-nucleosome arrangement analysis described by the center-to-center distance; **c** DNA linker length distributions, with dashed vertical lines for chromatin marks taken from ref. ^13^: heterochromatin (NRL: 205 bp), euchromatic gene bodies (NRL: 195 bp), active promoters and enhancer (NRL: ~178 bp); **d** Distance between connected nucleosomes r_i,i+1_; **e** Second-neighbor distance r_i,i+2_; **f** Angle ${{{{\rm{\theta }}}}}_{i}$ formed by the connected nucleosomes i, i + 1 and i + 2 (refer to panel **a**); **g** Correlation between r_i,i+2_ and ${{{{\rm{\theta }}}}}_{i}$. Magenta points are the values for the sequence of numbered nucleosomes from panel (**b**). Source data are provided as a Source Data file.

The connected chromatin fragments provide a close-up view of 3D heterochromatin organization. In the following, we will refer to consecutive nucleosomes as i, i + 1, …, i + n species (Fig. 4a), as seen in an exemplifying tetra-nucleosome arrangement from our tomograms (Fig. 4b). These data allow us to systematically determine both the center-to-center distance of consecutive nucleosomes (Fig. 4a) and the DNA linker length (Fig. 4b,c). The average center-to-center distance between consecutive nucleosomes, i and i + 1, was 26.2 nm. This corresponds to a ~ 37 nm edge to edge boundary and to nucleosome repeat lengths (NRLs) of ~200 bp (Fig. 4c). We also systematically quantified several parameters, including the i, i + 1 distance r_i,i+1_ (Fig. 4a, d), i, i + 2 distance r_i,i+2_ (Fig. 4a, e) and the i,i + 1,i + 2 angle *θ*_i_ (Fig. 4a, f). A prominent organizational feature is the stacking of the i and i + 2 nucleosome at an average center-to-center distance of 11 nm (Fig. 4e), whereby the connecting i + 1 nucleosome is in an opposing position. This is exemplified by nucleosomes 1-2-3 in Fig. 4b,g. STA confirmed two different types of stackings, with i and i + 2 nucleosomes either directly on top of each other or in a laterally shifted position (Supplementary Fig. 8). The center-to-center distance distribution of the i, i + 2 nucleosomes shows a second, even more prominent peak at an average distance of 28 nm, that is representative for non-stacking arrangements. This is exemplified by nucleosomes 2-3-4 in Fig. 4b, g.

The distribution of angles formed by three consecutive nucleosomes is bimodal with peaks at about 30 and 85 degrees (Fig. 4f, g). An angle of about 30 degrees is observed when the i and i + 2 nucleosome stack with each other, turning to 85 degrees without stacking. These two alternative tri-nucleosome arrangements are frequently re-occurring motifs, and often observed consecutively, as exemplified in (Fig. 4b). In a plot of the i, i + 2 nucleosome distance against the respective angle, which has been previously used to visualize the molecular determinants of chromatin folding in vitro^6^, two clusters are apparent (Fig. 4g). The two clusters are associated with preferred angles, given linker lengths, and stacking (Fig. 4b). Strikingly, these two clusters have been previously observed for tetra-nucleosomes in vitro^6^, showing that those constituting features of chromatin architecture can be reconstituted in simplified systems. This analysis suggests that the above-described parameters are the molecular determinants of chromatin folding in vivo.

**Fig. 5:**
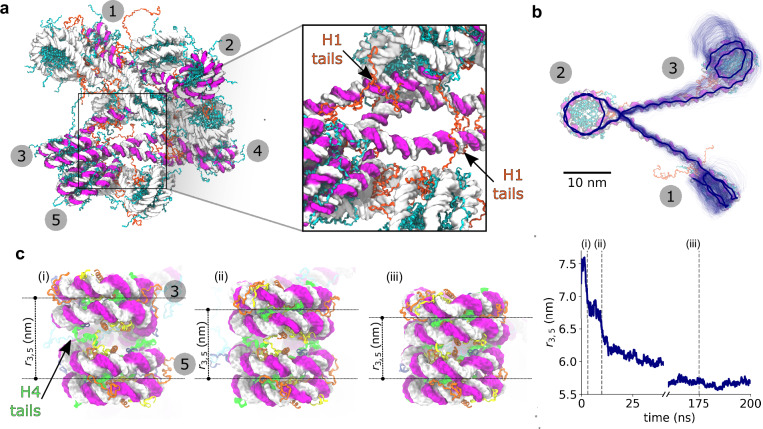
Molecular dynamics (MD) simulation of 13 chromatosomes, 5 of which are connected. **a** Exemplifying penta-nucleosome arrangement obtained from a tomogram and modeled in atomic detail, inset highlighting histone H1 tails. **b** Visualization of the dynamics of angle formed by a tri-nucleosome arrangement. **c** Formation of a nucleosome stacking interaction initially mediated by the H4 tail in (i), progression from (i) to (iii). Source data are provided as a Source Data file.

To explore the dynamics of those key molecular determinants, we selected a representative patch, modeled it in atomic detail and subjected it to all-atom molecular dynamics (MD) simulations (Fig. 5a). The overall molecular arrangement was stable throughout the simulated 200 ns, indicating a reasonable structural representation of the highly charged DNA and histones. The largest motion occurred at a terminal, singly connected nucleosome, which tightened its stacking interaction. The simulation illustrates the overall dynamic, but nonetheless well-defined ~37 nm wide arrangement. We observe changes in the angle of a tri-nucleosome of about 10° across the trajectory (Figs. 4f, 5b). Linker DNAs are correspondingly flexible (Supplementary Fig. 10c), in agreement with the assumptions of the linker prediction algorithm and the lack of longer DNA linkers in our chromatosome STA (Fig. 1g). The histone tails were initially modeled in random conformations (see “Methods” for details). As expected from their sequence rich in basic residues, they engage in multiple contacts with neighboring nucleosomes and linker DNA during the simulation (Supplementary Movie 3). Interestingly, in the MD simulations the histone tails also make contact with nucleosomes that are not directly proximate in the linear DNA chain. In particular, we observe the H1 tails binding to distant nucleosome cores and linker DNAs, promoting chromatin compaction (Supplementary Movie 3). Although we cannot prove that such interactions occur in cells, this observation illustrates that in a tomographically verified nucleosome arrangement such interactions have to be considered as a possible interaction. Furthermore, the formation of nucleosome stacking interactions is observed (Fig. 5c), partially mediated by H4-tail interaction with the histones of the neighboring nucleosome (Fig. 5c) as has been previously reported^33^.

## Discussion

We have found that nucleosomes can be confidently identified in cryo electron tomograms and that the trajectory of their DNA linkers can be estimated. Thus, our study demonstrates that the analysis of oligo-nucleosome arrangements is feasible in situ, and allows us to extract the key molecular determinants of higher-order heterochromatin structure. Overall, our analysis points to the following molecular traits as determinants: DNA linker lengths or NRLs, frequent nucleosome stacking, angular restraint by relatively inflexible DNA linkers and H1 binding, multi-valent contacts of histone tails to neighboring histones. Thereby some features, such as smaller NRL variability, smaller tri-nucleosome angles and angular restraining by H1 binding, promote nucleosome stacking and thus induce order. Conversely, larger NRL variability and larger tri-nucleosome angles will counteract nucleosome stacking, and thus induce disorder.

A regular 30 nm fiber has been suggested to be a major feature of heterochromatin in situ, but remains debated^3,7,8,11,22,34,35^. Whereas no evidence for an organized 30-nm fiber could be detected, we find a more heterogeneous, but still elongated structure with a characteristic width of ~37 nm. Differences to the aforementioned 30-nm fiber are that the DNA linker length observed in this study was on average ~17.5 nm, while a 30-nm fiber would require considerably shorter linker DNA. Furthermore, deviations in linker length, lateral displacement of stacked nucleosomes and occasionally occurring unusual angles break with the regularity of chromatin folding in situ. This results in a more heterogenous arrangement that is about 37 nm broad and elongated, thus appearing somewhat fibrous without showing the hallmark features of a highly ordered material (Fig. 2d).

We focused our analysis on the nuclear periphery, where heterochromatin^36^ is known to attach to the inner nuclear membrane^37–39^. STED imaging in human primary resting T cells showed a clear difference between the peripheral staining of a repressive histone mark (НЗK27me3) and the non-peripheral staining of an active histone mark (H3K4me3) (Supplementary Fig. 2). Therefore, it is likely that transcriptionally inactive chromatin is prominently contained in our data. Traditional imaging techniques such as light microscopy or plastic embedding electron microscopy suggest that the heterochromatin layer underneath the nuclear envelope is variable in thickness, but may considerably extend into the nucleus, such that active chromatin may be outside our present field of view (Supplementary Fig. 2). This would explain why our extensive efforts to template match polymerases and chromatin remodelers, did not yield any statistically compelling results. Interestingly, we did observe a layer of chromatin that was firmly attached to the nuclear envelope (Fig. 1a, b). This morphologically distinct layer was only about 50 nm thick, and is thus beyond the diffraction limit for light microscopy.

Our in situ structural analysis can be compared directly to genomics data. We quantified DNA linker length in our tomograms, finding on average a nucleosome repeating length (NRL) of ~200 bp in resting T cells (Fig. 4c). While our data only capture the nuclear periphery, genomics methods were previously used to estimate nucleosome spacing in T cells in a genome-wide and histone-mark specific manner^13^. The spacing for chromatin marks H3K27me3, H3K9me3 attributed to heterochromatin (NRL: 205 bp) and H3K27me1^40,41^ attributed to euchromatic gene bodies (NRL: 195 bp) was higher in comparison to H3K4me1 and H3K27ac (NRL: ~178 bp) that were attributed to enhancers and super enhancers^42^. The distribution in our data considerably overlaps with the values expected for heterochromatin (Fig. 4c).

In addition, we here demonstrate the power of cryo-ET to analyze smaller complexes in the range of only about 250 kDa at subnanometer resolution. This may have broader applications and enable the in situ structural analysis of a range of biological processes. For example, one could envision our approach being used to compare different cellular states associated with, e.g., cell differentiation, genetic and pharmacological perturbations, or in response to external stimuli. Further technical improvements in tomographic data acquisition and processing schemes should yield cryo electron tomograms of active chromatin in the near future, thus opening up the exciting possibility of observing gene expression at work. Such data may also allow pinpointing histone variants by extensive image classification combined with molecular simulations, and to spatially associate them with proximate regulatory complexes. Thus, genomics and in situ structural biology may go hand in hand in the future towards improving our understanding of chromatin structure and function relationships. Taken together, our study bridges the understanding of chromatin from molecular to oligo-nucleosome arrangements and illustrates the potential of in situ structural biology in contributing to future mechanistic analyses of chromatin compaction and remodeling.

### Limitations of the study

One limitation of our cryo-ET study is that we cannot observe dynamics. Chromatin is known to be a dynamic structure albeit less so in heterochromatic regions^43–46^. Each tomogram can therefore be seen as one chromatin state frozen in time out of many possible configurations within a cell and between cells. In addition, we estimated the accuracy of the orientations and positions of the nucleosome particles only with global metrics for the entire ensemble, but not for each individual particle. Therefore, due to local fluctuations in SNR the accuracy of the linker DNA prediction may locally differ. Nevertheless, given that our linker prediction correlates well with the experimental data (Supplementary Fig. 10d), particles with poor alignments likely make up only a minor fraction of all particles.

In this study, the majority of the detected nucleosomes appear to be H1-bound. We based this interpretation on the observation that extensive classification efforts did not yield highly resolved subtomogram averages of H1-free nucleosomes (Supplementary Fig. 6). However, a minor subpopulation of H1-free nucleosomes likely exists in the nuclear regions we analyzed, leading to a certain degree of nucleosome heterogeneity. At this stage, we cannot fully exclude the possibility that the performance of our algorithm is negatively influenced by the presence of such H1-free nucleosomes with more flexible linker arms, even if they are not prominent in our samples. The angular distribution of the detected nucleosomes was biased towards side views (Supplementary Fig. 3c), where the thin edge of the nucleosome disk showing the two DNA gyres is parallel to the electron optical axis. These findings are consistent with a recent study on Chlamydomonas reinhardtii, which also reported nucleosome structures in situ^24^. This will affect the number of false negative nucleosomes, thus resulting in an underestimation of nucleosomes present in the sample. However, since our algorithm estimates probabilities in pairs, discards non-confident assignments, and chromatin orients freely in the nucleus, we do not expect a strong influence on the estimated linker length pattern.

## Methods

### Ethics statement

Human peripheral blood of healthy donors was obtained from the blood bank Heidelberg upon informed consent and according to regulation by the ethics committee of the Medical faculty, Heidelberg University (ethics votes S-604/2020 and S-025/2022).

### Preparation of primary CD4 + T cells

Detailed CD4 + T cells purification method used is described in Lucic et al., 2019^47^. Briefly, cells were obtained by using RosetteSep Human CD4^+^T cells enrichment cocktail (STEMCELL technologies) and isolated by Ficoll gradient centrifugation. Following the lysis of red blood cells, purified T cells were cultured in RPMI-1640 10% FBS and penicillin/streptomycin, in the presence of IL2 10 ng/µL and incubated at 37 °C in a 5% CO_2_ atmosphere. Cells were then transferred to 20 mM Hepes pH 7.9 RPMI-1640 at a concentration of 4 × 10^6^ cells for further use.

### Immunofluorescence staining of CD4 + T cells

CD4 + T cells were seeded on PEI (0.5 mg/mL) pre-treated coverslips for 45 minutes at 37 °C in a 5% CO_2_ atmosphere and rinsed with PBS. Cells were then fixed with 4% PFA/PBS for 10 minutes at room temperature and washed three times with PBS. Permeabilization was performed by incubating the coverslips with 0.5% Triton X-100/PaBS for 10 minutes at room temperature. After three washes in PBST (0.1% Tween-20/PBS), blocking was carried out with 4% BSA/PBS for 1 hour at room temperature.

Coverslips were subsequently incubated overnight at 4 °C with the following primary antibodies diluted in 1% BSA/PBST: anti–lamin B1 (Santa Cruz, sc-365962; 1:100), anti–H3K27me3 (Cell Signaling Technology, 9733S; ~1:300), and anti–H3K4me3 (Millipore, 04-745; 1:100). The next day, coverslips were washed three times in PBST and incubated with secondary antibodies (Sigma: ATTO 76085 anti-mouse IgG 594 and ATTO 40839 anti-rabbit IgG 647 N; 1:200 in 1% BSA/PBST) for 1 hour at room temperature, protected from light.

Finally, cells were washed three times in PBST, incubated with Hoechst for 10 minutes at room temperature, rinsed in PBST and water, and mounted with Mowiol.

### STED microscopy

STED images of fixed cells were acquired using an Expert Line STED system (Abberior Instruments GmbH, Göttingen, Germany) coupled to an inverted Olympus IX83 microscope body, using an UPlanSApo 100x/1.4 N.A. oil immersion objective and Imspector v16.3 microscope-control software. Line sequential mode was used to acquire STED images using the 561 nm and 640 nm excitation laser and APD detectors with the spectral detection window set between 587–630 nm for ATTO594 imaging and between 650–754 nm for ATTO 647 N imaging. In addition, confocal images were acquired using the 405 nm excitation laser with a spectral detection window between 415–551 nm. A pulsed donut-shaped (SLM easy3D module) 3 W 775 nm depletion laser was employed at 30% (H3K27me3) or 50% (H3K4me3 and lamin B1) with pixel dwell time of 10 μs, 3-6x line accumulation and 1 AU pinhole. STED images were deconvoluted in Huygens Professional (Scientific Volume Imaging) using the express deconvolution option with the conservative strategy. Signal quantification was performed using a Fiji (ImageJ2, version 2.16.0/1.54p) macro based on consecutive concentric rings of 100 nm width generated from a lamin mask (thresholded and filtered) using a distance map. To exclude neighboring cells that were out of focus or incomplete, the ‘Clear Outside’ option was applied prior to analysis to the manually selected region of interest (ROI). Mean gray values were recorded for each ring and normalized to the mean intensity of the entire nucleus. Graph was created using GraphPad Prism version 10.6.0 (796).

### Cell vitrification and cryo-FIB milling

3.5 µL of cell suspension of resting primary human CD4 + T cells was applied on glow discharged 200-mM mesh EM gold grids (Quantifoil Micro Tool GmbH) coated with R 2/2 holey SiO2 films and plunge frozen in liquid ethane –183 °C using a Leica EM GP2 grid plunger (Leica Microsystems) with the blotting chamber at 37 °C. The grids were blotted from the back (cell-free) side for 4-5 seconds with a Whatman filter paper, Grade 1 before plunge freezing.

The cells were then cryo-FIB milled using an Aquilos 2 microscope (Thermo Fisher Scientific) in a similar fashion to a previously described workflow^48^. In brief, samples were coated with an organometallic platinum layer for 10 sec and additionally sputter coated with platinum at 1 kV and 10 mA current for 10 sec. The lamella milling was performed with AutoTEM (version 2.4.2) (Thermo Fisher Scientific) in a stepwise manner with a 30 kV ion beam while reducing the current from 1000 pA to 50 pA. Final polishing was performed with 30 pA current with a lamella target thickness of 200 nm.

### Cryo-ET data acquisition

Cryo-ET data for resting T-cells were collected at 300 kV on a Titan Krios G4 microscope (Thermo Fisher Scientific) equipped with a E-CFEG, Falcon 4 direct electron detector (Thermo Fisher Scientific) operated in counting mode and Selectris X imaging filter (Thermo Fisher Scientific). Montaged grid overviews were acquired for each grid with a 205 nm pixel size. Individual lamellae montages were taken with 3 nm pixel size. Tilt series were acquired using SerialEM (version 4.0.20)^49^ in low dose mode as 4 K x 4 K movies of 10 frames each and on-the-fly motion-corrected in SerialEM. The magnification for projection images of 64000x corresponded to a nominal pixel size of 1.971 Å. Tilt series acquisition was started from the lamella pretilt of ± 8° and a dose-symmetric acquisition scheme^50^ with 2° increments grouped by 2 was applied, resulting in 61 projections per tilt series with a constant exposure time and targeted total dose of ~135 e^-^ per Å^2^. The energy slit width was set to 10 eV and the nominal defocus was varied between -1.75 to -4.25 µm. Dose rate on the detector was targeted to be ~5 e^-^ /px/sec.

### Tomogram reconstruction

Dose exposure correction for the motion corrected tilt series was performed as previously described^51^ using a Matlab implementation that was adapted for tomographic tilt series^52^. Poor quality projection images were removed after visual inspection. The dose-filtered tilt series were then aligned with patch-tracking in AreTomo (v2.0)^53^ and reconstructed as back-projected tomograms with SIRT-like filtering of 15 iterations at a binned pixel size of 7.884 Å (bin4) in IMOD (version 4.11.5)^54^. The 14 tomograms containing a nuclear envelope were selected for further processing. These tomograms were then denoised with the cryoCARE software package^55^ for improved visualization.

For the same tomograms, reconstruction with 3D-CTF correction was also performed using novaCTF^27^ with phase-flip correction, astigmatism correction and 15 nm slab. Tomograms were binned 2x and 4x using Fourier3D^56^ and M^57^ these 14 tilt series were reprocessed in Warp^58^ with the alignment obtained from AreTomo^53^.

### Nucleosome template matching, including initial template generation

To obtain initial particle coordinates and orientations, the bin2 (3.942 Å/px) 3D-CTF corrected tomograms were template matched with an existing nucleosome structure (EMD-3947) (lowpass filtered to 13 Å) and 5 degree global angular sampling in GAPSTOP^TM^^28,29^. The resulting constrained cross correlation (CCC) volumes were used to extract positions and coordinates of peaks with a CCC score 4.5 sigmas above the mean CCC score value. The candidate particles determined through template matching were extracted as subtomograms in Warp (v1.09)^58^ at bin2. Subsequent junk classification and refinement in Relion (v3.1)^32^ lead to an initial STA nucleosome structure that was then used as an improved search template for further template matching.

To improve template matching performance, we used the cryoTIGER computational workflow^26^ to interpolate between tilt stacks using the FILM algorithm^59^, as detailed in ref. ^26^. The aligned bin2 tilt stacks were used as inputs for cryoTIGER where one tilt image was interpolated in between two real tilt images for each stack. The combined stacks containing real and interpolated tilt images were used for novaCTF^27,29^. Since all tomograms contained both cytoplasm and nucleoplasm the two regions were manually segmented in IMOD)^54^ for each tomogram. The constrained cross-correlation (CCC) peaks in both regions of the tomogram could thereby be compared and a peak extraction threshold for nucleoplasmic peaks could be set per tomogram. This threshold corresponded to the 99^th^ percentile of CCC values for cytoplasmic peaks in each tomogram (Fig. 1d), allowing for extraction of a high-confidence nucleosome particle list. This particle list was further cleaned by excluding clashing particles with a nucleosome shape mask around each peak in cryoCAT^31^.

### Nucleosome subtomogram averaging

The 33,560 particles determined through template matching were extracted as bin1 (1.971 Å/px) subtomograms in Warp)^58^ (no interpolation between tilt stacks) and subjected to subtomogram averaging and alignment in Relion 3.1^32^. Multiple attempts at classifying out junk particles were made, all resulting in no significant percentage of particles being classified as junk. The particle set was then imported into M^57^ to perform multi-particle refinement of the tilt-series and the nucleosome. Geometric and CTF parameters were refined in a sequential manner. Afterwards, new M-corrected bin1 subtomograms were again refined in Relion 3.1^32^. After a more precise calibration of the TEM at the given 64,000x magnification, the pixel size was determined to be 1.895 Å/px and the final resolutions were determined in Relion 3.1^32^ postprocessing with the new calibrated pixel size. This resulted in a chromatosome structure resolved to 7.4 Å with C1 symmetry and a 6.8 Å chromatosome structure with C2 symmetry imposed (at FSC 0.143 cutoff). Locally, the chromatosome with C2 symmetry imposed was resolved up to 6.0 Å. The particles refined with C1 symmetry were furthermore classified based on potential stacking neighbors and around the H1 linker histone binding site.

### Orientation bias correction factor calculation

To assess the extent of the orientation bias, we adapted existing code from the pyem python package^60^ to assign particle orientations to angular bins utilizing the HEALPix library. Each nucleosome orientation was assigned to one of 768 HEALPix bins (HEALPix order value = 3), then all bins with counts below the mean+1σ bin count value were filled up to that value. This approach yielded a correction factor of about 2.14X to get a balanced angular distribution of nucleosomes.

### Nucleosome classification for H1-free subpopulation

To assess the presence of an H1-free subpopulation of nucleosomes an extensive workflow was developed. First, template matching with 5 degree global angular sampling in GAPSTOP^TM^ ^28,29^ was performed on bin2 3D-CTF corrected tomograms enhanced with the cryoTIGER workflow^26^ (described above in “Methods” section about nucleosome template matching) with two different nucleosome templates, one with H1 density included, the other without it. To obtain the “no H1 included” template, the density of H1 was manually removed from the “with H1” template described above using the ChimeraX^61^ eraser function to only leave a core nucleosome density. Next, candidate particles were identified for both sets of TM volumes with a less stringent CCC threshold corresponding to the 95^th^ percentile of CCC values for cytoplasmic peaks in each tomogram (thresholding described above in “Methods” section about nucleosome template matching). The candidate subtomograms (62,665 for H1-including template, 57,390 for H1-free template) were extracted as bin1 subtomograms in Warp^60^ (no interpolation between tilt stacks). After merging the two particle sets and then distance cleaning using a nucleosome shape mask in cryoCAT^31^, a combined 70,663 candidate subtomograms were transferred to Relion3.1^32^ for 3D classification with a featureless cylinder as initial reference to filter out junk. This removed 17,071 particles and further 3D classification of the remaining 53,592 nucleosomes particles revealed two classes. There was one clear chromatosome class with defined linker arms and clear H1 density (class1 with 31,005 particles) and one partial chromatosome with less defined linker arms and only partial H1 density (class 2 with 22,587 particles). No class without any H1 density could be identified. The two class particle sets were then imported into M^57^ to perform multi-particle refinement of the tilt-series and the nucleosome. This only improved the resolution for the full chromatosome not the partial chromatosome class. Afterwards, new M-corrected bin1 subtomograms were again refined in Relion 3.1^32^. After a more precise calibration of the TEM at the given 64,000x magnification, the pixel size was determined to be 1.895 Å/px and the final resolutions were determined in Relion 3.1 postprocessing with the new calibrated pixel size. This resulted in a full chromatosome structure resolved to 6.9 Å with C1 symmetry and a partial chromatosome structure resolved to 12.6 Å with C1 symmetry (at FSC 0.143 cutoff).

### Nucleosome Analysis

To determine the organization, spatial properties and connectivity of heterochromatin at the nuclear periphery inside T-cells, we used the 33,560 positions obtained from subtomogram averaging and M refinement (with C1 symmetry) for further spatial analysis and linker prediction.

### Clustering Analysis

To determine how nucleosomes group together within heterochromatin, we performed cluster analysis of the positions of the nucleosomes using the hierarchical density-based spatial clustering of applications with noise (HDBSCAN)^62^ clustering algorithm, with a minimum cluster size of 7. HDBSCAN is a density-based clustering algorithm suitable for data with variable densities. For the visualization, each identified cluster was plotted in a unique color to distinguish it from others (Fig. 2a).

### Fraction of the chromatin-free nuclear volume

To further quantify heterochromatin compaction, we used Monte Carlo integration to estimate the fraction of the nuclear volume that is chromatin-free. First, we defined the volume of the nucleoplasm by manually segmenting the nucleus in each tomogram using IMOD^54^. We estimated the nuclear volume by calculating the volume of the convex hull that surrounding the segmented mask. We then attempted to draw 10,000 random particles within the convex hull of the nucleus, with a radius of 5 nm. To determine the relative volume occupied by nucleosomes, we repeated this process taking into account the positions of nucleosomes, which were modeled as spheres of 5 nm radius. A particle was accepted only if it did not collide with more than two nucleosomes. The latter was used to account for isolated nucleosomes. Finally, we calculated the ratio of accepted particles from the nucleosome-inclusive generation to the total number of random particles generated within the nucleoplasm.

### Pair distribution function

To quantify the spatial organization and analyze the local structure of nucleosomes, we calculated the normalized pair distribution function, $g\left(r\right)$ per tomogram. This function quantifies the probability of finding a pair of nucleosomes separated by a distance *r* and allows us to determine the degree of spatial ordering or clustering of nucleosomes.

The normalized pair distribution function, $g\left(r\right)$, was calculated as the ratio:

4 $$g\left(r\right)=\frac{{g}_{{nucleosome}}\left(r\right)}{{g}_{{random}}\left(r\right)}$$

where ${g}_{{nucleosome}}\left(r\right)$ is the pair distribution function obtained from a histogram of nucleosome positions, and ${g}_{{random}}\left(r\right)$ is a reference pair distribution function, computed from randomly drawn particles within the convex hull of the nucleosome positions. To mimic a random fiber-like arrangement, the 12-particle coordinate set describing one 30-nm fiber segment from the map EMD-2601^7^ was used as a rigid template. 6000 copies were placed in a 0.7 µm³ box with random orientations and translations while rejecting clashes. From this list of particles, $g\left(r\right)$ was obtained as described above.

### Linker prediction

We have developed a physics-based algorithm to infer linker connections between nucleosomes in tomograms. The algorithm utilizes the positions and orientations of nucleosomes obtained from STA after M-refinement to compute the energy required for linker formation, describing the DNA with the wormlike chain (WLC) model. Based on these energy calculations, probabilities are determined for all possible nucleosome pairings and their respective linker configurations. Linkers are then assigned iteratively, beginning with the configurations of the highest probability. The subsequent sections describe the theory, implementation details, and algorithm validation.

### Theory

Nucleosomes are connected by double-stranded (ds)DNA linkers. Single-molecule elasticity measurements have shown that the entropic elasticity of dsDNA in aqueous solutions is accurately described by the WLC model with a fixed contour length^63–65^. Deviations of the chain from its straight configuration result in a bending energy ${U}_{{bend}}$^66^. For in-plane bending to second order, we approximate the DNA bending energy^67^as:

5 $${{{{\rm{U}}}}}_{{{{\rm{bend}}}}}\left({{{\rm{\theta }}}},L\right)=\frac{2{l}_{p}}{L}{\left(\frac{{{{\rm{\theta }}}}}{2}\right)}^{2}{k}_{B}T$$

where ${l}_{p}\approx$ $50$ ${{{\rm{nm}}}}$ is the persistence length of DNA^68^ at physiological conditions, $L$ its length, $\theta$ its bending angle, ${k}_{B}$ the Boltzmann constant, and $T$ the temperature. We use Eq. (5) to estimate the energy required to bend a segment of DNA into a linker between the selected arms of a pair of nucleosomes.

### Implementation

Starting from the nucleosome positions and orientations obtained from STA after M refinement, we want to determine the most likely trajectory of the DNA that connects two nucleosomes. From the map obtained from STA (Fig. 1e-g), the approximate direction of the two linker arms in the nucleosome is determined (See Supplementary Fig. 9a). We denote the linkers of the nucleosome *i* as: ${{{\rm{Linker}}}}{1}_{i}$, and ${{{\rm{Linker}}}}{2}_{i}$. There are 4 possible ways $\Gamma$ to connect a pair of nucleosomes (*i, j*), that is: $\Gamma \in \,[({{{\rm{Linker}}}}{1}_{i}:{{{\rm{Linker}}}}{1}_{j}),({{{\rm{Linker}}}}{1}_{i}:{{{\rm{Linker}}}}{2}_{j}),({{{\rm{Linker}}}}{2}_{i}:{{{\rm{Linker}}}}{1}_{j}),({{{\rm{Linker}}}}{2}_{i}:{{{\rm{Linker}}}}{2}_{j})]$. For each pair of nucleosomes *i,j*, and each combination of k $\in \Gamma,$ we determine the probability of this connection as:

6 $${P}_{{ijk}}=P\left({L}_{{ijk}},{\theta }_{{ijk}}\right)=\left({e}^{\frac{-{L}_{{ijk}}}{{L}_{0}}}\right)\left({e}^{\frac{-\Delta {U}_{{bend}}({\theta }_{{ijk}},{L}_{{ijk}})}{{k}_{B}T}}\right)$$

Note that the length ${L}_{{ijk}}$ and the angle ${\theta }_{{ijk}}$, depend on the case $\Gamma$ (see Supplementary Movie 1). In Eq. (3), we penalize long linker lengths as our uncertainty increases. We set ${L}_{0}$ = 15 nm as an average value of the expected linker length since DNA linkers range from ~20-90 bp and vary among different species, tissues, and even fluctuate within a single cellular genome^69^.

To determine ${P}_{{ijk}}$, we computed ${L}_{{ijk}}$ and ${\theta }_{{ijk}}$ for a given connection k$\in \Gamma$ from the tangent vectors ${{{\bf{ T}}}}_{i}$, ${{{\bf{ T}}}}_{j}$ for given linkers in $i,j$ and the connecting vector ${{{\bf{ D}}}}_{{ijk}}$ (See Supplementary Fig. 9a).

### Calculation of the probabilities *P*_*ijk*_

To identify the most likely nucleosome connections, we computed the probabilities ${P}_{{ijk}}$, for all pairwise combinations and cases. To reduce the computational cost, the particle list was pre-processed using cryoCAT^31^. Using the previously described chain tracing algorithm^70^, nucleosomes that are spatially close to each other were grouped into chains. For the chain tracing, nucleosome coordinates were shifted to the position of bp 1 and bp 147 (points a_i_ and a_j_ in Supplementary Fig. 9a), generating two coordinate sets per nucleosome. Tracing decisions were based solely on the distance between a_i_ and a_j_, with a maximum distance of 20 nm. For each traced cluster *M*, pairwise probability “matrix” ${P}_{M}\in {{{{\rm{R}}}}}^{N\times N\times 4}$ was calculated (See Fig. 3b and Supplementary Fig. 9c), where |M | = N is the number of particles in the traced cluster, and the third dimension of the matrix corresponds to the four possible configurations, |$\Gamma$|=4. We use the verb ‘trace’ here to describe the connection of nucleosomes into patches of chromatin by interpolating the DNA linkers in-between them.

### Assignment of linker connections

From the matrix *P*, we iteratively established the connectivity between particles. First, we defined:

$$C=\{{C}_{1},{C}_{2},\ldots,{C}_{N}\}$$

where ${C}_{{{{\rm{i}}}}}$ represents the set of nucleosomes that are connected to nucleosome *i* and the configuration $\Gamma$ of the connection. Each particle can have at most one connection at linker 1 and one at linker 2.

For the iterative assignment, we identified the position (*i,j,k*) with the highest probability ${P}_{{ijk}}=\max \left(P\right)$ and checked if the connection (i ↔ j) was possible based on the topological constraints mentioned above. If the connection is valid, we added (i ↔ j) to ${C}_{i}$, and (j ↔ i) to ${C}_{j}$.

Next, we set ${P}_{{ij}0}={P}_{{ij}1}={P}_{{ij}2}={P}_{{ij}3}=0$ and ${P}_{{ji}0}={P}_{{ji}1}={P}_{{ji}2}={P}_{{jj}3}=0$, thereby removing the pair of nucleosomes from future iterations. This process continued until no more connections were possible, imposing a probability threshold, ${P}_{{ijk}} > 0.1$. At each iteration, it was also checked that there are no closed loops.

The connectivity structure $C$ can be represented as a graph $G(V.E)$ where, V are the nucleosomes (nodes) and E are the established connections (edges). See Fig. 3c and Supplementary Fig. 9c.

### Validation of the predicted linkers

Using the final connectivity graph *G*, we estimated the coordinates and orientation of the linker centers. A particle was placed at the geometric center between the predicted connected arms, and the orientation of the linkers was assigned to align with the vector connecting the corresponding linker arms (See Supplementary Fig. 9a).

### Subtomogram averaging of the predicted linkers

We performed STA in Relion3.1^32^ on the 7163 predicted linkers using the positions and orientations derived from the algorithm (see above) with subtomograms being extracted with Warp^58^, as before no interpolation was used for the STA. The average of the extracted subtomograms was used as the initial reference to avoid biasing the refinement. As a further test, we grouped the linkers based on their predicted length (Fig. 4c) and performed STA on each group (see Supplementary Fig. 10), resulting in cylindrical shapes with lengths consistent with the predictions.

### Validation and robustness analysis of the DNA linker prediction algorithm on in vitro chromatin reconstructions

To quantify the robustness of the linker-prediction pipeline against localization and angular uncertainty, we generated synthetic motive lists by adding isotropic Gaussian noise to the particle coordinates of the in vitro EMD-2601^7^ structure. Positional, and angular noise was independently swept in the range [0,30] Å and [0, 30] deg, respectively. For each σ we created 50 independent realizations and ran the complete workflow. Statistic over the linker predictions was determined via precision = (TP/(TP + FP)), recall = (TP/(TP + FN)) and the harmonic-mean F1 score, by comparing the predicted edge set with the ground-truth set obtained from the noise-free case.

### Robustness of linker prediction algorithm to false negatives

We quantified the robustness of the linker-prediction pipeline to false negatives by systematically removing subsets of nucleosomes. Starting from the list obtained by fitting our STA nucleosome to the EMD-2601^7^ volume, we deleted sets of k nucleosomes (k = 1, 2, 3 or 4) and reran the full linker-prediction pipeline. We then compared the new predictions with the ground truth of linkers and report precision, recall, and the F1 score. Note that for scoring, we excluded all linkers incident to the removed nodes; in other words, scores were calculated only on the linkers that could be predicted using the set of particles present. For k = 1,2 all possible combinations were evaluated, (i.e., n = 12, 66, respectively). For k = 3,4, and N = 12, we used a random subset of 100 combinations of the $$\left(\begin{array}{c}N\\ k\end{array}\right)$$ combinations (n = 100).

### ${{{{\boldsymbol{P}}}}}_{{{{\boldsymbol{max}}}}}/{{{{\boldsymbol{P}}}}}_{{{{\boldsymbol{second}}}}}$ of the predicted linkers

To evaluate the certainty of linker assignments in the connectivity analysis, we recorded the highest probability ${P}_{\max }$ and the second highest probability ${P}_{{second}}$, at each iteration of the algorithm where a linker was assigned. Specifically, ${P}_{\max }=\max \{{P}_{{ijk}}|\forall i,j,k\}={P}_{{i}_{0}{j}_{0}{k}_{0}}$ represents the highest probability for the assigned connection between a pair of particles *i* and *j*, reflecting the most confident linkage based on the computed probabilities. On the other hand, ${P}_{{second}}=\max \{{P}_{{i}_{0}{jk}}|\forall j,k\ne {j}_{0},{k}_{0}\}$ is the second-highest probability value from the same row in the probability matrix, serving as a measure of competing linkage possibilities. A large ratio ${P}_{\max }/{P}_{{second}}$ indicates a more confident linkage assignment (see Supplementary Fig. 10a).

### Analysis of the connected nucleosomes

To investigate the structural organization and spatial relationships of connected nucleosomes, we analyzed the spatial distances, and angular relationships between particles in 3D. First, we calculated the first-neighbor distance (r_i,i+1_): as the Euclidean distance between directly connected particles. We then measured the second-neighbor distance (r_i,i+2_) between particles separated by one intermediate nucleosome. r_i,i+2_ is the distance between the center of particles *i* and *i* + *2* within sorted connected components. Finally, we evaluated the angle (θ_i_) between connected triplets of nucleosomes by computing the angle between particles *i, i* + *1*, and *i* + *2* in sorted connected components. The angle was determined using the dot product of vectors connecting the particles *i, i* + *1* and *i* + *1, i* + *2*.

### All-atom model of the connected nucleosome structures

Starting from the STA positions of the nucleosomes, and the predicted linkers, we created all-atom models of the nucleosomes, linkers and intrinsically disordered regions (IDRs) of the histone tails. To model the predicted linkers, we adapted the previously established hierarchical chain growth (HCG) ^71^method, which has been used to efficiently sample the conformational space of disordered proteins and single-stranded RNA^72^. In the present implementation, we generated a library of input fragments by taking the central seven base pairs of 10,000 regularly spaced frames from a previously published molecular dynamics simulation of a 33 base pair B-DNA helix^73^. At each stitching move, fragments are aligned along backbone heavy atoms of one of two overlapping base pairs. A move is accepted if the alignment RMSD is below a 0.6 Å cut-off. With this, we generated a library of ensembles of 10,000 structures each at a range of possible linker lengths.

For each pair of connected nucleosomes, we then screened the library by aligning three base pair backbones on either end with the respective entry/exit points of the nucleosomal DNA. We considered structures that yield an alignment RMSD below a 2.5 Å cut-off to be suitable candidate structures. For assembly of the full system, we then drew randomly from candidate sub-ensembles at the shortest length that had at least 20 candidate structures until a clash-free combination was found.

In a final step, we added histone tails to the structure by randomly drawing from HCG ensembles and checking for clashes with the rest of the system. We used human histone-tail sequences to match the folded portions included in the template nucleosome. We created the HCG ensembles using a publicly available implementation (https://bio-phys.pages.mpcdf.de/hcg-from-library/)^74^ that draws from a pre-sampled library of dipeptide fragments covering the full sequence space.

### All-atom molecular dynamics simulations of the chromatin segment

We performed molecular dynamics simulations of a system of 13 nucleosomes with predicted linkers modelled explicitly using the Gromacs 2024.2 engine^75^ with the Amber14SB force-field^76^ augmented with Parmbsc1 parameters^77^. Systems were solvated with TIP3P water^78^, 150 mM KCl and 5 mM MgCl_2_ ensuring overall neutrality. We used Mamatkulov-Schwierz parameters for potassium and chloride ions^79^ and Grotz et al. parameters^80^ for magnesium ions. After energy minimization, we equilibrated the system in the NVT ensemble at 310 K for 5 ns at a 1 fs timestep using Berendsen temperature coupling^81^. We performed further equilibration in NPT at 1 bar for 20 ns at a 2 fs timestep using C-rescale pressure coupling. We ran the production simulation for 200 ns using velocity-rescale temperature coupling^82^ and Parrinello-Rahman pressure coupling^83^. We used frames every 100 ps for analysis.

To illustrate the dynamics of the system, we drew DNA base pair traces every 5 ns starting at 10 ns by aligning the central nucleosome of a trimer with the xy-plane and calculating the center of mass of complementary base pairs across the trajectory. For visualization we rendered the trimer at 70 ns (Fig. 5b). To illustrate linker dynamics, we traced the duplex helical axis between nucleosomes 4 and 5 (see Fig. 5a in the main text) after aligning on the two connected nucleosomes. Frames extracted every 5 ns (Supplementary Fig. 10c). Distances r_i,i+2_ and angles θ_i_ were calculated between centers of mass of the 147 base pairs of each nucleosome. To illustrate linker dynamics, we traced the duplex helical axis between nucleosomes 4 and 5 (see Fig. 5a in the main text) after aligning on the two connected nucleosomes.

### Reporting summary

Further information on research design is available in the Nature Portfolio Reporting Summary linked to this article.

## Supplementary information

Supplementary Information

Description of Additional Supplementary Files

Supplementary Movie 1

Supplementary Movie 2

Supplementary Movie 3

Reporting Summary

Transparent Peer Review file

## Source data

Source Data

## Data Availability

The chromatosome STA maps reported in this paper are deposited in the Electron Microscopy Data Bank (EMDB) with the following accession codes: EMD-58100 (chromatosome with C1 symmetry applied) (https://www.ebi.ac.uk/emdb/EMD-58100) and EMD-58101 (chromatosome with C2 symmetry applied) (https://www.ebi.ac.uk/emdb/EMD-58101). The lower resolution STA maps used for template matching are deposited at Zenodo (10.5281/zenodo.20184093). For the T cell cryo-ET dataset, the raw frames, files for tomogram reconstruction, and 4x binned reconstructed tomograms are deposited on EMPIAR with accession code EMPIAR-13566 (https://www.ebi.ac.uk/empiar/13566). The MD simulation data are deposited at Zenodo (10.5281/zenodo.20266252). Source data are provided with this paper Source data are provided with this paper.

## Code availability

The code for DNA linker prediction is deposited at github (https://github.com/sergiocruzleon/DNA_Linker_prediction).
