# Supplementary material for: Molecular architecture of heterochromatin at the nuclear periphery of primary human cells
Source: Nat Commun. 2026 Jul 3;17:5844. doi: 10.1038/s41467-026-75087-5 (PMC13332202; doi:10.1038/s41467-026-75087-5)
Supplement: Supplementary file 1 — Supplementary Information [file 41467_2026_75087_MOESM1_ESM.pdf]

Supplementary Information for

Molecular architecture of heterochromatin at the nuclear periphery of primary human cells

Authors: Jan Philipp Kreysing[1,2]† Sergio Cruz-León[3]†, Johannes Betz[3]†, Carlotta Penzo[4], Tomáš Majtner[1], Markus Schreiber[1,2], Beata Turoňová[1,*], Marina Lusic[4,*], Gerhard Hummer[3,5,*], Martin Beck[1,6,*]

† These authors contributed equally to this work.

Corresponding authors: beata.turonova@biophys.mpg.de, marina.lusic@med.uni-heidelberg.de, gerhard.hummer@biophys.mpg.de, martin.beck@biophys.mpg.de

The PDF file includes:

Supplementary Figures 1 to 14
Supplementary Table 1

Other Supplementary Information for this manuscript include the following:

Supplementary Movies 1 to 3

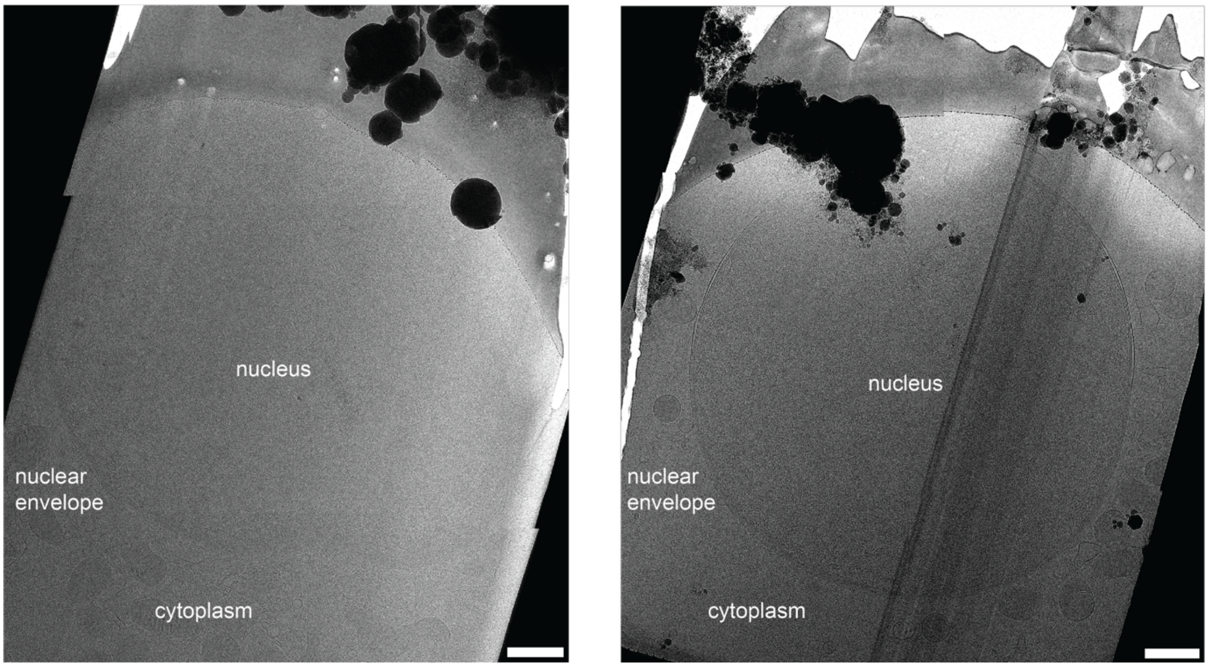

Supplementary Fig. 1. Lamella maps. Low-magnification lamella maps for two different lamellae with nucleus, nuclear envelope, and cytoplasm indicated. There are no apparent visual differences in terms of chromatin density inside the nuclei. All tilt series were collected at the nuclear envelope. Scale bar indicates 1 µm.

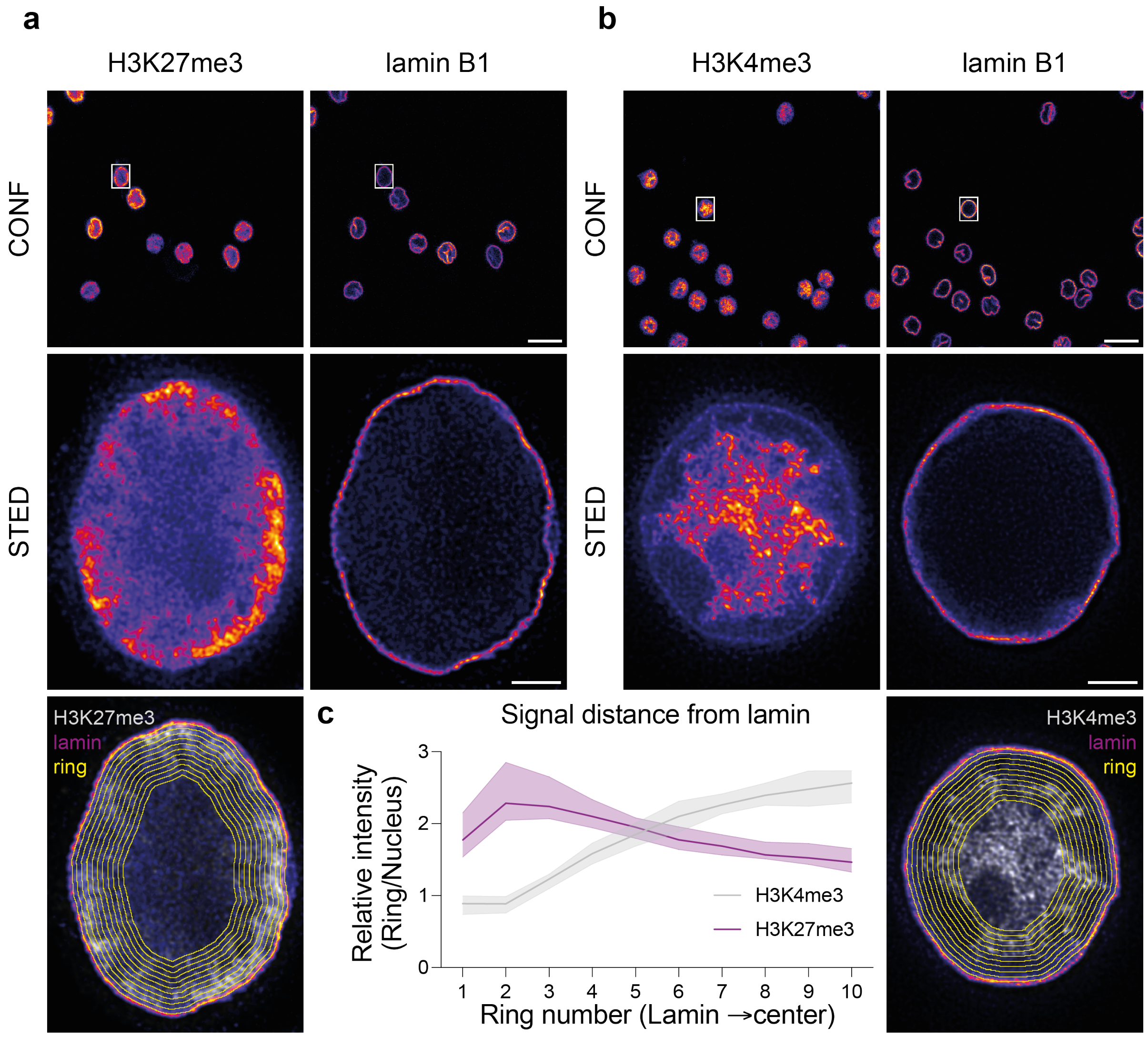

Supplementary Fig. 2. STED imaging of chromatin markers in resting CD4+ T cells and quantitative analysis. a-b Fire LUTs of lamin B1 and H3K27me3 (**a**) or H3K4me3 (**b**) signals. The top panels show a broad confocal view (CONF, scale bar = 10 μm), the middle panels a deconvoluted STED acquisition of a central Z-plane enlargement (STED, scale bar = 1 μm). The bottom panels display the channel overlap, with fire LUT for lamin B1, gray LUT for the histone mark, and ten concentric rings of 100 nm width generated from the lamin signal (yellow). **c** Quantification of signal intensity (mean gray values) measured in the indicated rings and normalized to the mean signal of the entire nucleus. The distance between rings is 100 nm. Graphs represent the median (solid line) and interquartile range (shaded area) of pooled cells from two independent blood donors (violet: H3K27me3, n = 8 and 7 cells from each donor; gray: H3K4me3, n = 12 and 10 cells). Source data are provided as a Source Data file.

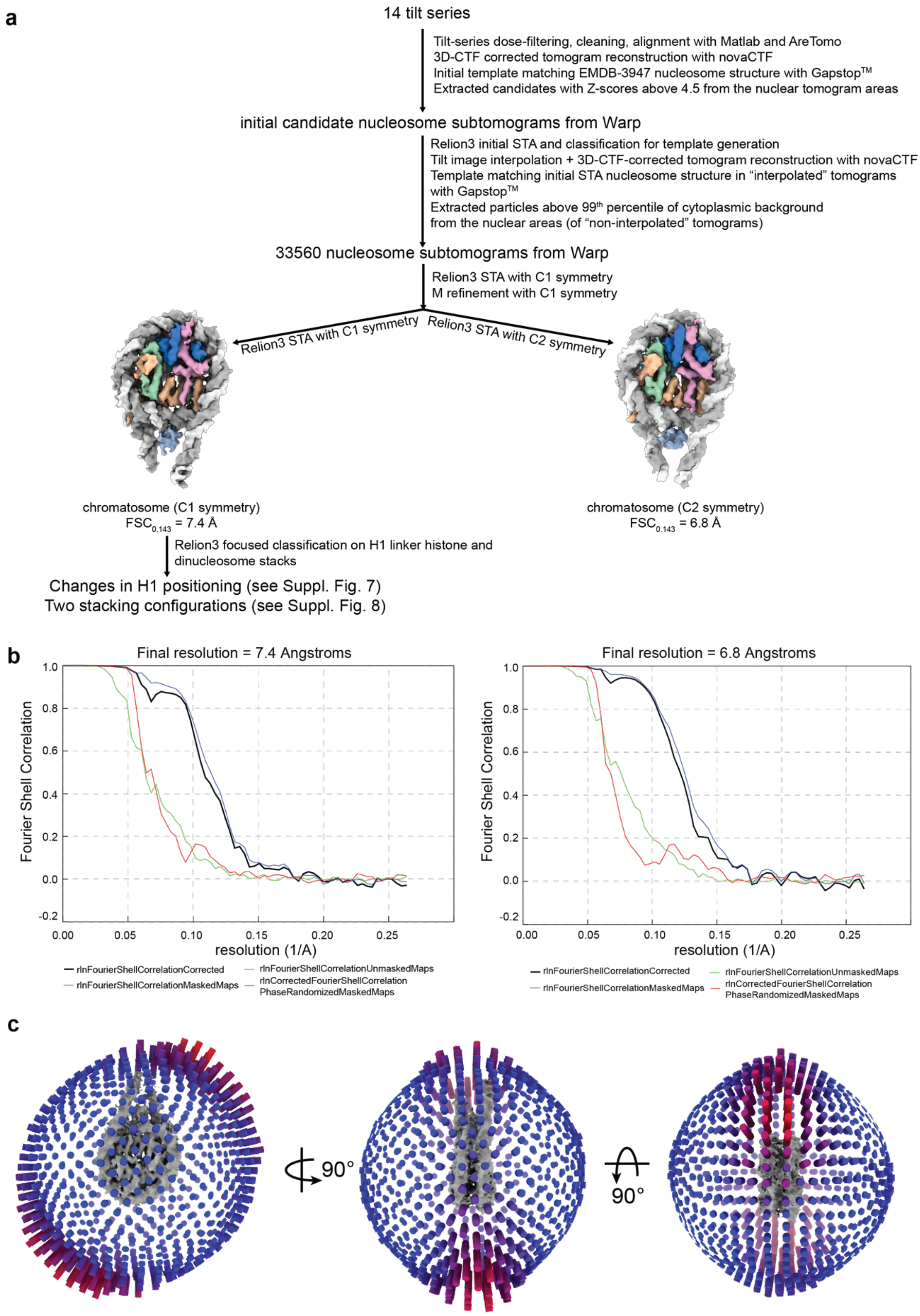

Supplementary Fig. 3. Image processing workflow for the chromatosome. a Workflow describing the steps taken from raw tilt series to final chromatosome STA structures. **b** Fourier shell correlation curves from Relion 3.1 [32] after M refinement[57] for structures with C1 or C2 symmetry imposed. **c** The angular distribution of the detected nucleosomes exhibits a bias towards side views, where the thin edge of the nucleosome disk showing the two DNA gyres is parallel to the electron optical axis. Source data are provided as a Source Data file.

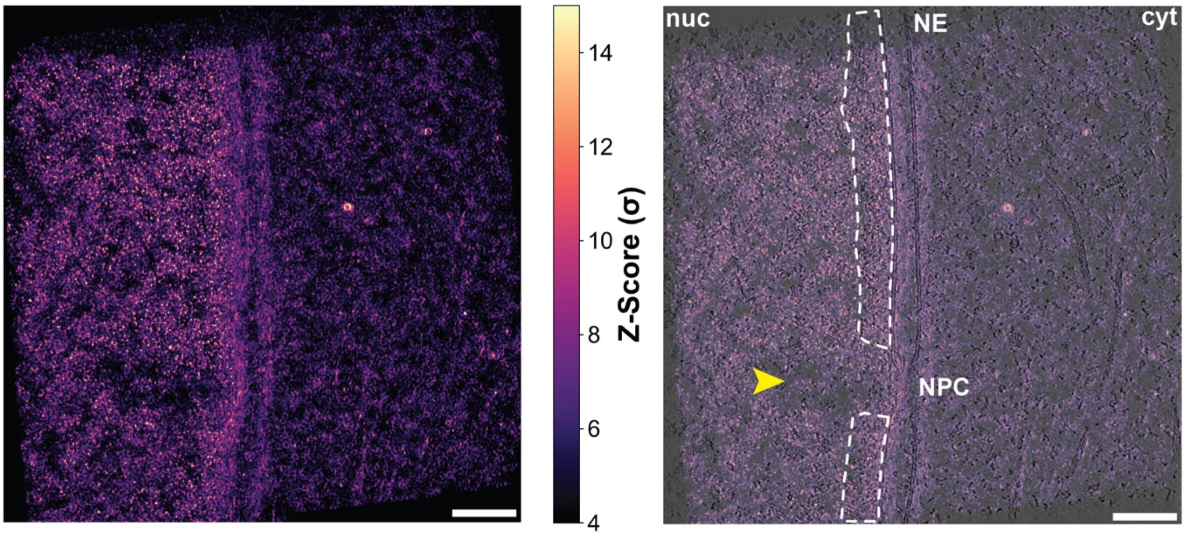

Supplementary Fig. 4. Template matching nucleosomes with GAPSTOP^{TM} [28,29]. Constrained cross-correlation (CCC) volume (transformed to Z-scores) from template matching nucleosome structure on an exemplary tomogram, shown as a maximum intensity projection and color-coded by Z-score. The number of and intensity of the peaks in the nuclear region of the tomogram is much higher as can be seen in the overlay of the CCC volume with a tomogram slice. Chromatin-free space is indicated with a yellow arrow, a dense layer of chromatin underlying the nuclear envelope is framed white.

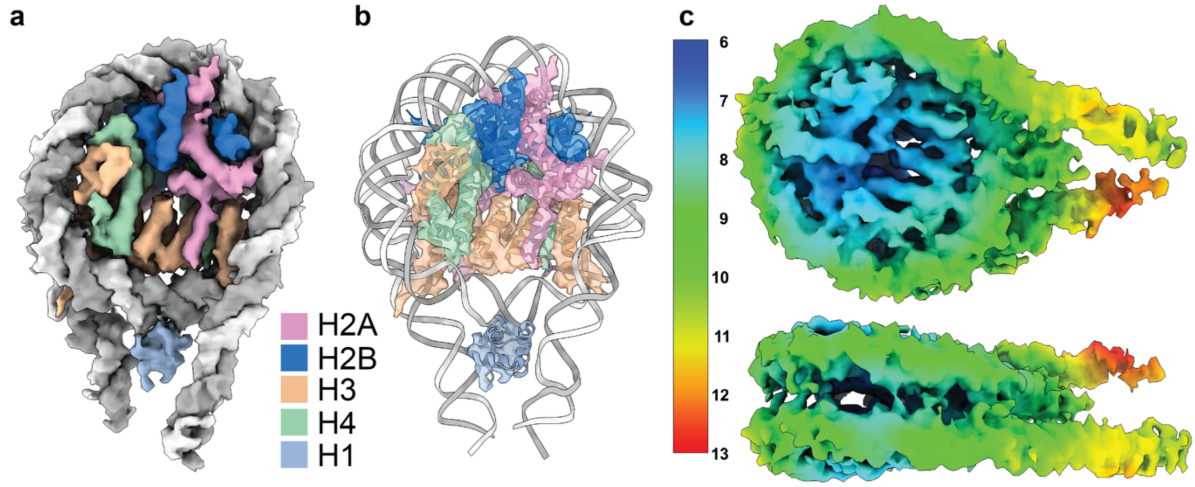

Supplementary Fig. 5. STA of the chromatosome with C1 symmetry. a Color-coded STA map by DNA strands (white and gray) and histone proteins H2A (pink), H2B (blue), H3 (amber), H4 (green) and H1 (light blue). **b** The atomic model of the canonical chromatosome (PDB: 7dbp[30]) was fitted into our map and is shown in the middle panel. **c** Color-coded local resolution STA map of the human chromatosome (from high resolution in blue to low resolution in red, in Å).

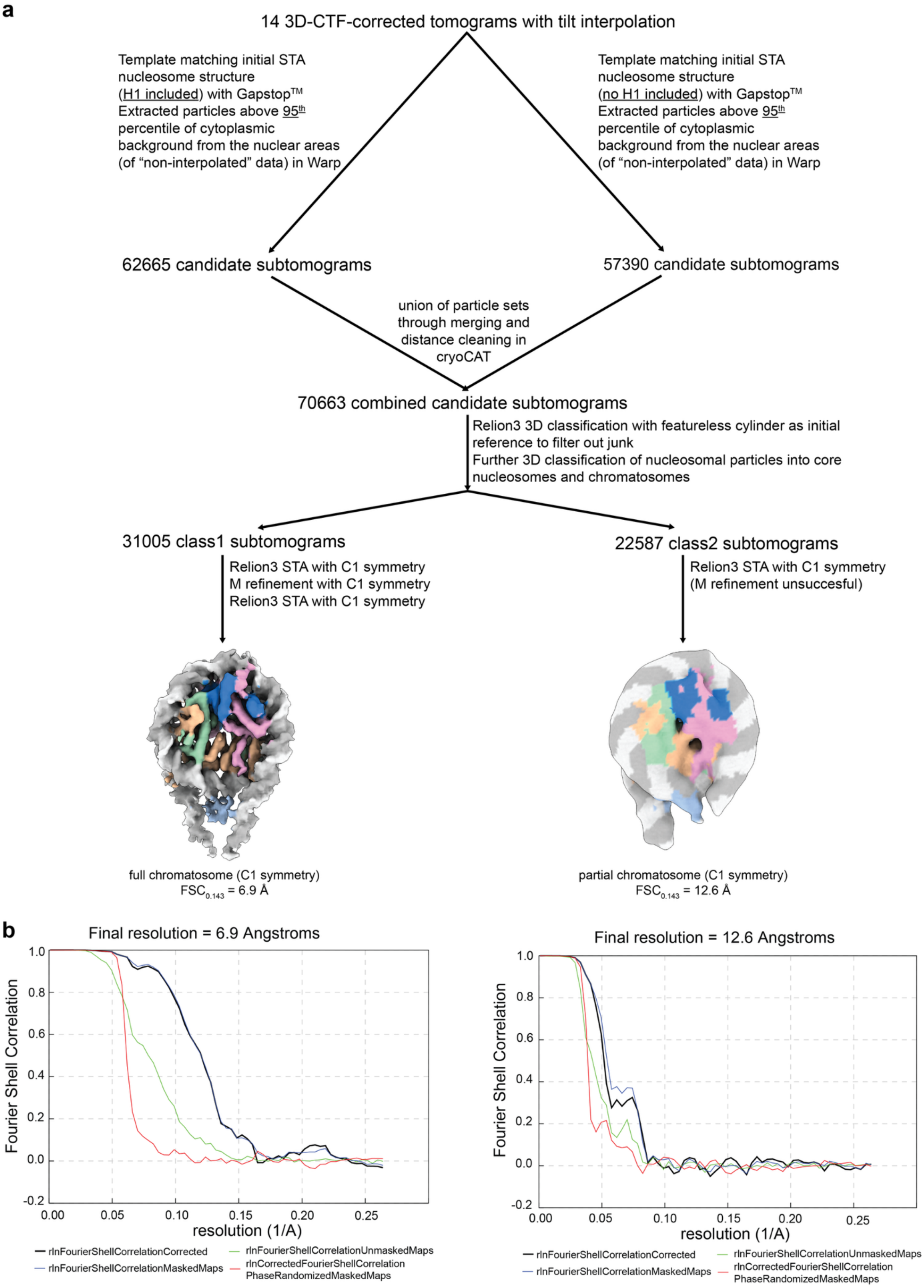

Supplementary Fig. 6. Processing workflow for extensive 3D classifications to find H1-free nucleosome class. a Workflow describing the steps taken to classify a H1-free nucleosome population. **b** Fourier shell correlation curves obtained from Relion 3.1[32] , for full and partial chromatosome averages. Source data are provided as a Source Data file.

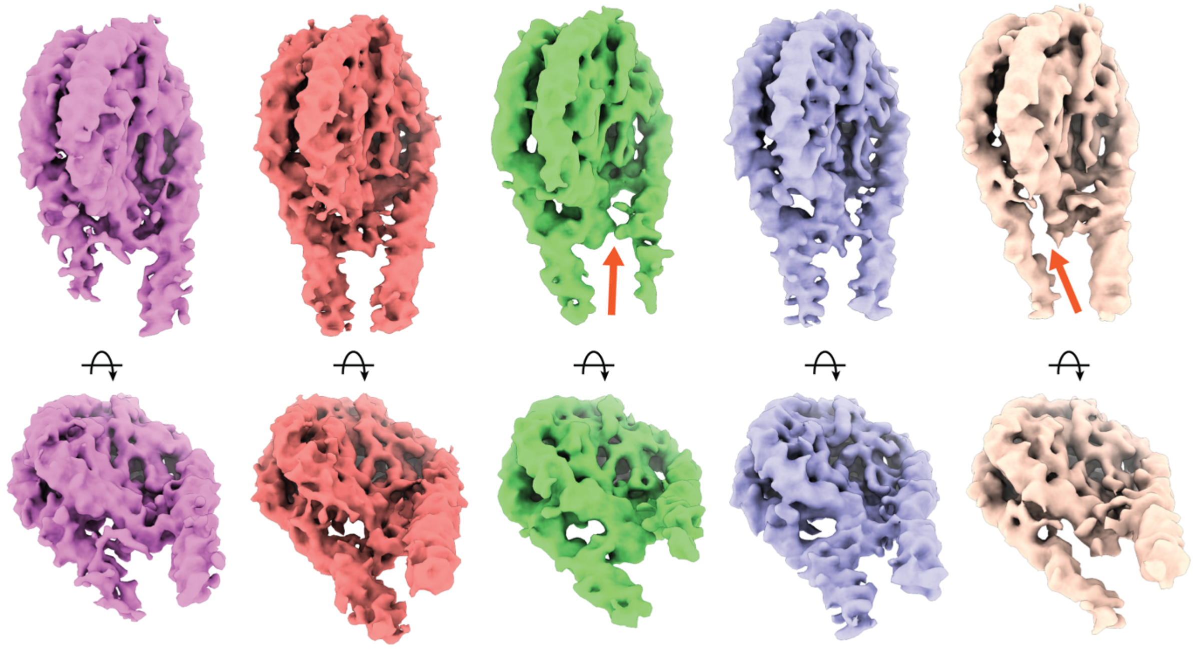

Supplementary Fig. 7. Focused 3D classification around H1 linker histone area reveals shifting H1 histone. 3D classification in Relion 3.1[32] identified nucleosome classes in which H1 was shifted to either DNA linker (highlighted with orange arrows).

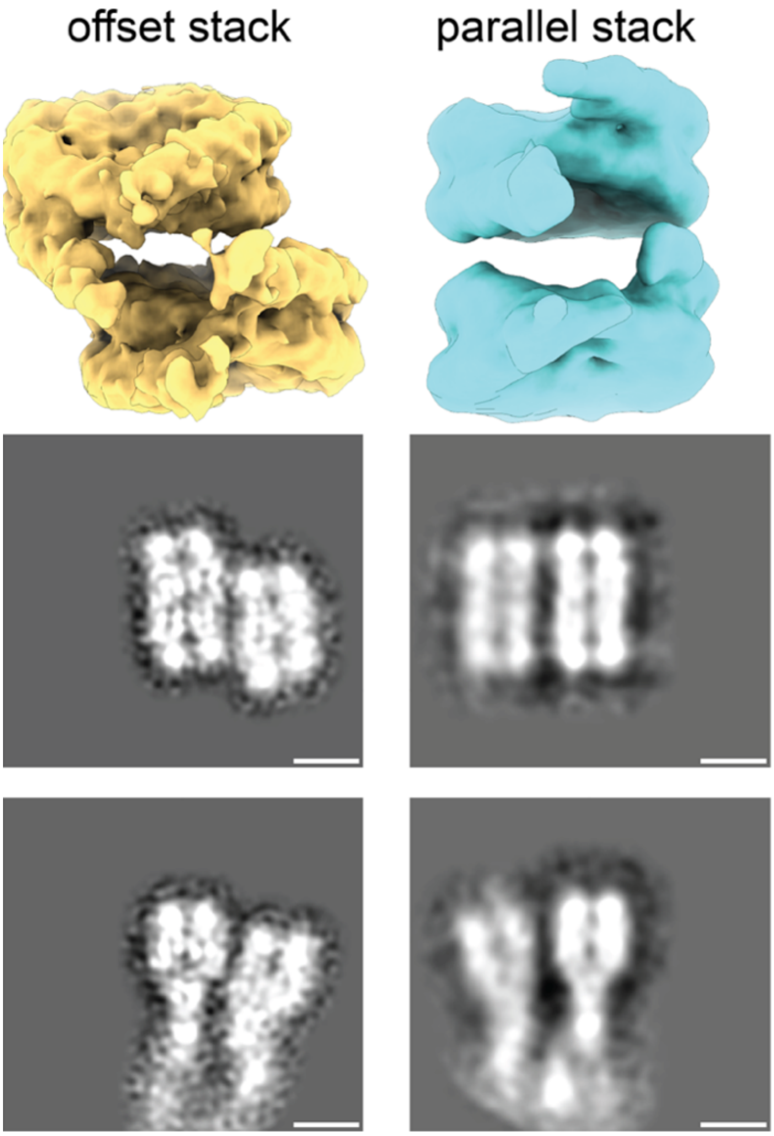

Supplementary Fig. 8. 3D classification of stacked nucleosomes reveals two stacking configurations. 3D classification in Relion 3.1[32] found that ~18% of all nucleosomes were in one of two stacking configurations. Stacking configuration 1 shows the particles stacked with an offset whereas stacking configuration 2 shows the particles stacked directly on top of each other. The center-to-center distance between the stacked nucleosomes was ~6.5 nm. Scale bars: 5 nm.

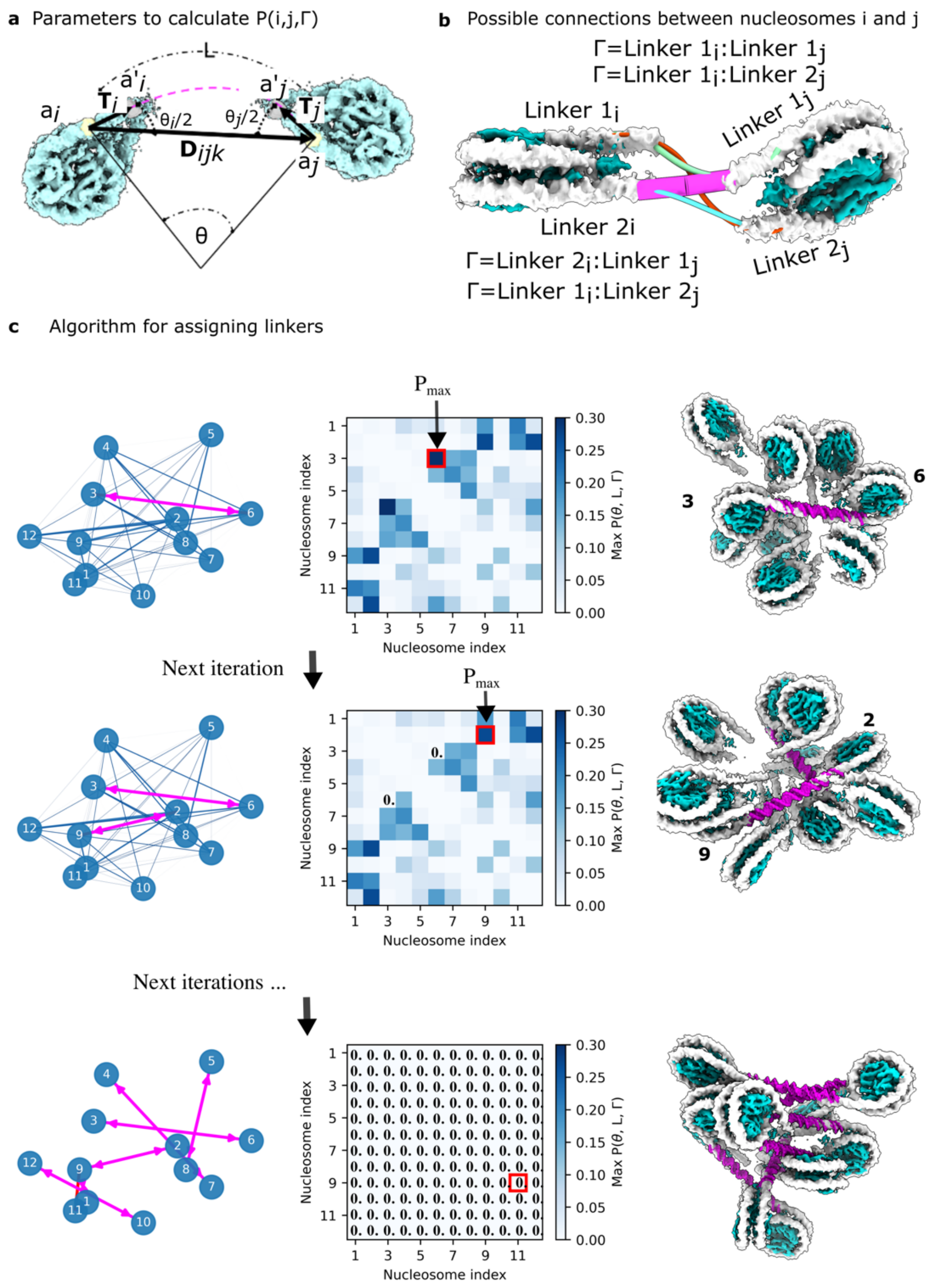

Supplementary Fig. 9. Visualization of the linker assignment. a Scheme for determining the parameters to for the probability of linkers. The probability of a potential linker (pink dashed line) to exist is calculated from the bending angle, θ, and the arc length L. For each pair of nucleosomes *i,j*, and each combination Γ of connections between the arms, the length L is determined as the arc length measure from the center of bp 1 or bp 147 in the nucleosome (points a_i, and a_j marked with yellow spheres). The bending angle is determined as $\theta = \frac{\theta_i + \theta_j}{2}$. **b** Set of possible connections Γ between a pair of nucleosomes (*i, j*). **c** Algorithm for assigning linker connections. The columns show the graph of the nucleosomes (left), the probability matrix P (middle) obtained as described in Methods, and the visualization of the nucleosome position and the predicted linkers (right). The linkers are assigned iteratively, starting with the configurations with the highest probability P_{max}. In the example shown the linker (3↔6) is created in the first iteration. For the next iteration, we set $P_{36k} = P_{63k} = 0, \forall\ k\ \in [0,1,2,3]$, and now P_{max} creates the connection (2↔9). The process continues until no further connections are possible with a threshold $P_{ijk} > 0.1$. Note that the indices of the particles are arbitrary.

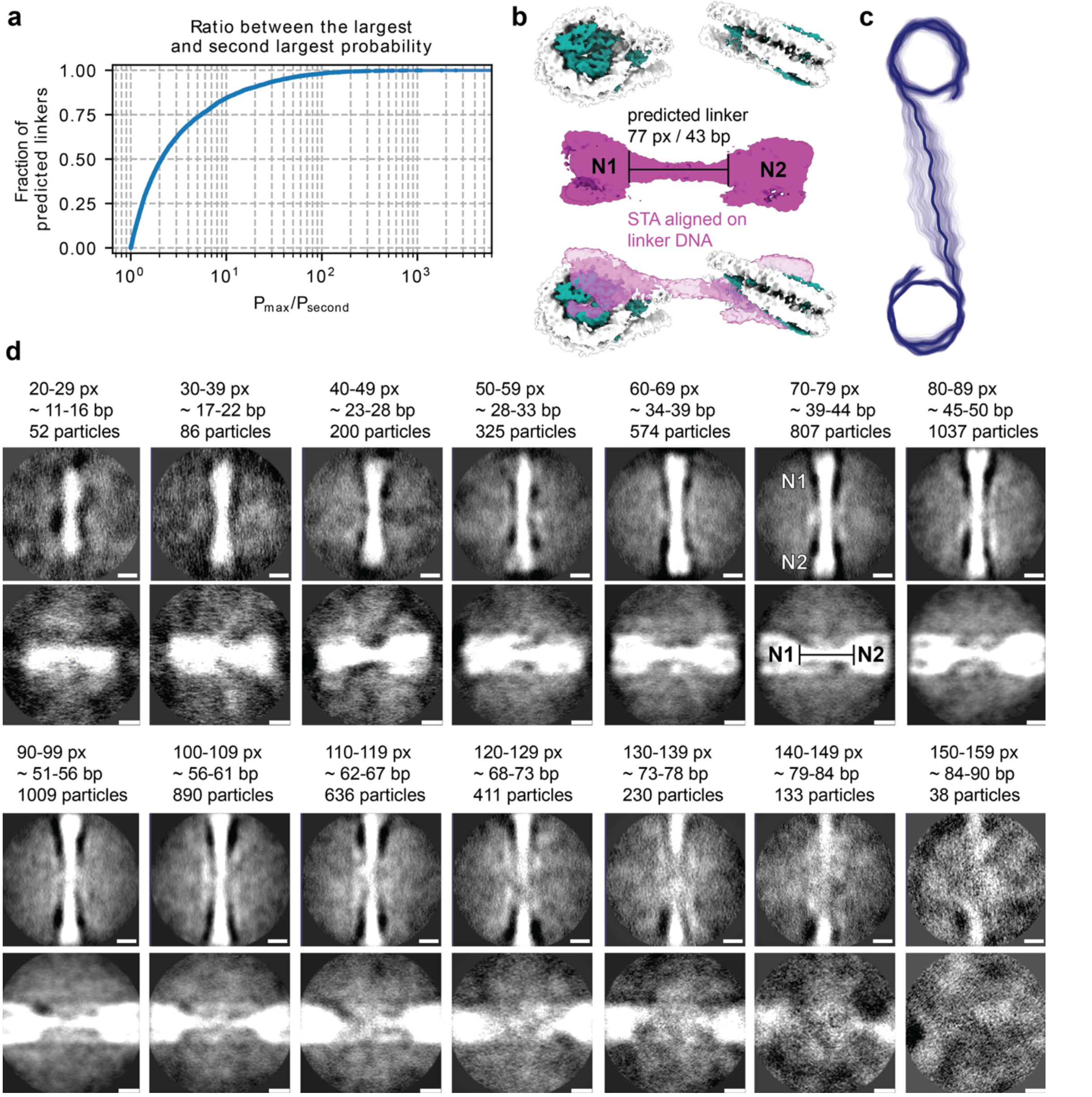

Supplementary Fig. 10. Predicted DNA linker validation through P_{max}/P_{second} and STA. a Cumulative distribution of the ratio between the highest probability P_{max} and the second highest probability P_{second}, for all the assigned linkers (see Methods). This ratio quantifies competing linkage possibilities, with larger values indicating higher confidence in the linkage assignment. **b** (top) Visualization of two nucleosomes predicted to be connected by a 77 px / 43 bp long linker (positions and orientations obtained through STA, DNA in transparent grey and histones in teal). (middle). Isosurface representation of STA map from all linkers with lengths between 70-79 px in pink (see also **d** and Methods) The nucleosomes (N1 and N2) are not well resolved due to the alignment being centered on the linker DNA. (bottom) Visualization of same two nucleosomes with the 70-79 px linker average placed according to STA positions and orientations**. c** Visualization of the conformational ensemble of a 73-bp linker DNA derived from MD simulations. Lines trace the duplex helical axis between nucleosomes 4 and 5 (see Fig. 5a in the main text); thin lines denote individual simulation frames extracted every 5 ns, and the thick line marks the average configuration. **d** The DNA linkers were grouped by predicted length and subjected to separate STA refinements in Relion 3.1 [32]. YZ and XZ slices through each linker DNA average are shown with the predicted length matching well to increasing length of the densities. Scale bar in **d** indicates 5 nm. Source data are provided as a Source Data file.

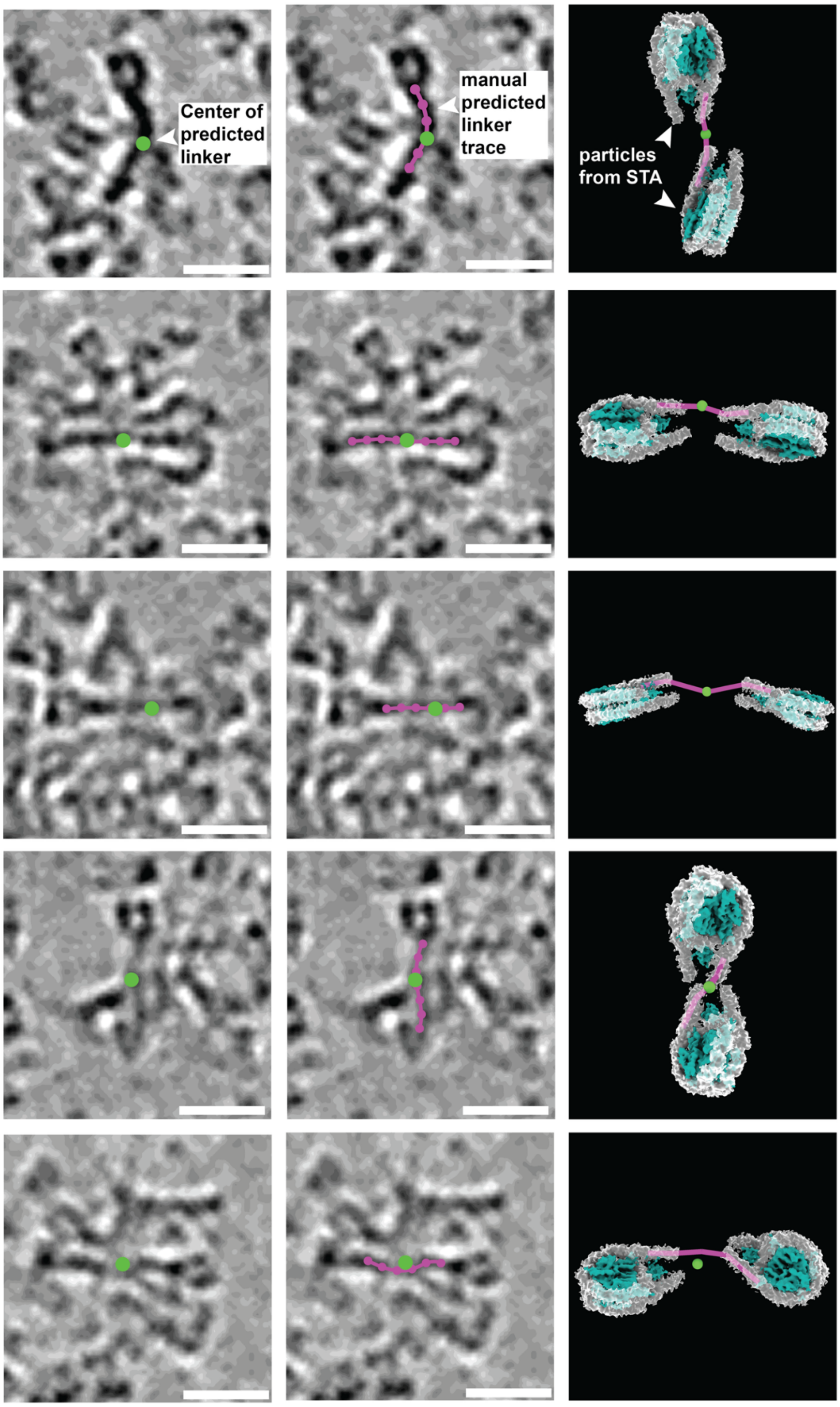

Supplementary Fig. 11. Overlay of predicted DNA linker positions on tomographic slices. From left to right for five predicted linkers, with the center of the predicted linker indicated by a green dot: (leftmost) Tomographic slice showing a DNA linker density and connected nucleosomes; (middle) Same tomographic slice with manually traced DNA linker to illustrate plausible path in pink; (rightmost) Visualization of the nucleosomes (positions and orientations obtained through STA, DNA in transparent grey and histones in teal) and a manually placed linker in pink. Note: The center of the fifth predicted linker is slightly shifted compared to the visually traceable linker density. Scale bar in all slices indicates 20 nm. All tomograms were denoised with cryoCARE [55].

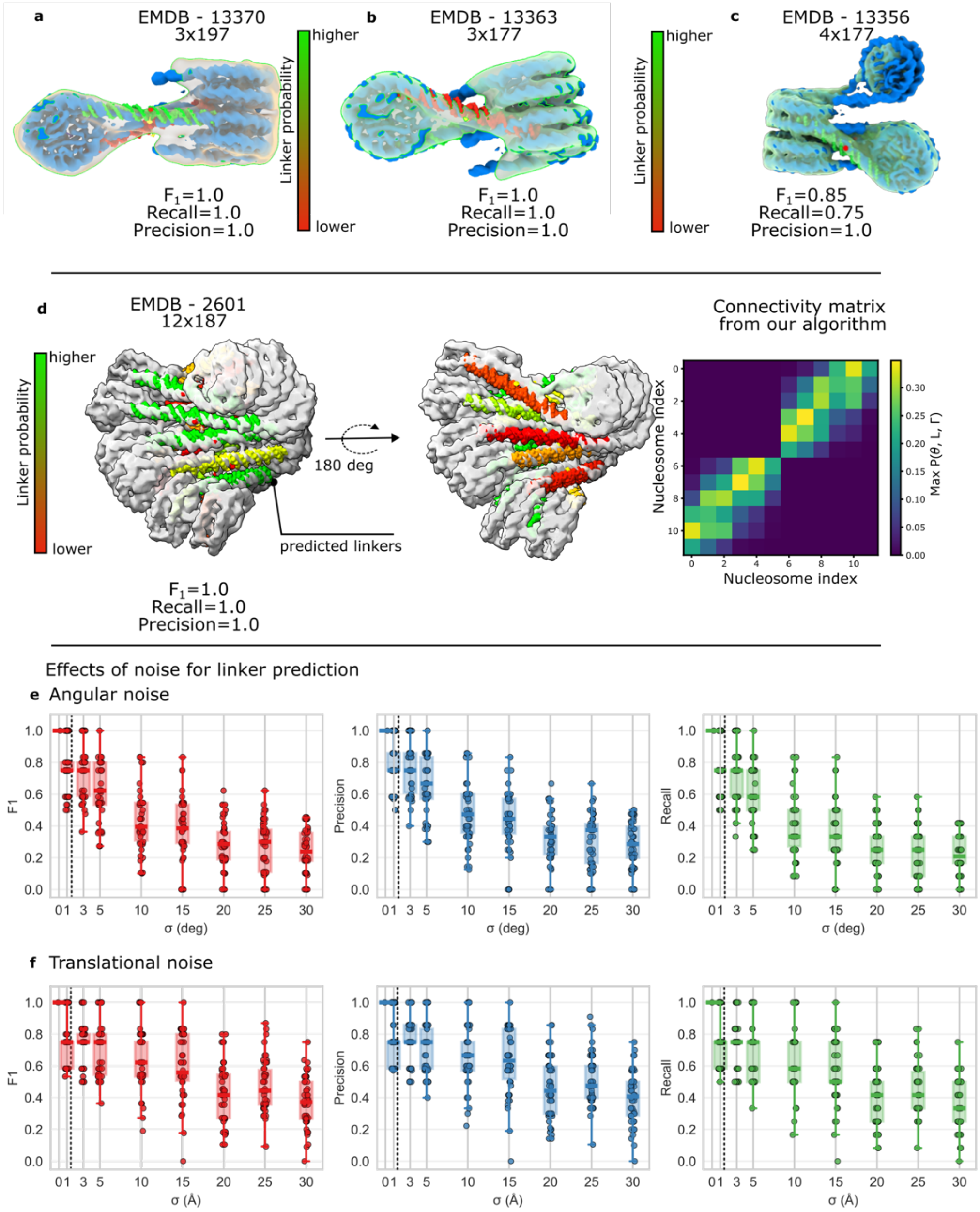

Supplementary Fig. 12. Validation and robustness analysis of the DNA linker prediction algorithm on in vitro chromatin reconstructions. (a-c) Predicted linkers for three in vitro chromatin assemblies (EMD-13370[6], EMD-13363[6], EMD-13356[6]) shown overlaid on their EM density maps. Linkers are colored by probability (green = higher, red = lower). (d) Example prediction on a compact 12-mer (EMD-2601[7], with linker probabilities mapped onto the density and corresponding connectivity matrix (Pmax) for the 12-mer. Note that in cases where the predicted linkers are depicted in red, the experimentally observed density shows a significant degree of bending. (e–f) Robustness analysis of the linker predictions under increasing levels of angular (e) and translational (f) noise (σ = 0°–30°), and (σ = 0–30 Å), respectively. We report F1 score, recall, and precision. Each data point represents one of 50 independent runs per noise level (n=50); box plots show median, interquartile range, and outliers. We find that F1, precision, and recall drop below 0.5 on average for noise levels of ~10 degrees (orientation) and 15-20 Å (translation). These thresholds define the required localization precision for meaningful linker prediction using the elastic model. Source data are provided as a Source Data file.

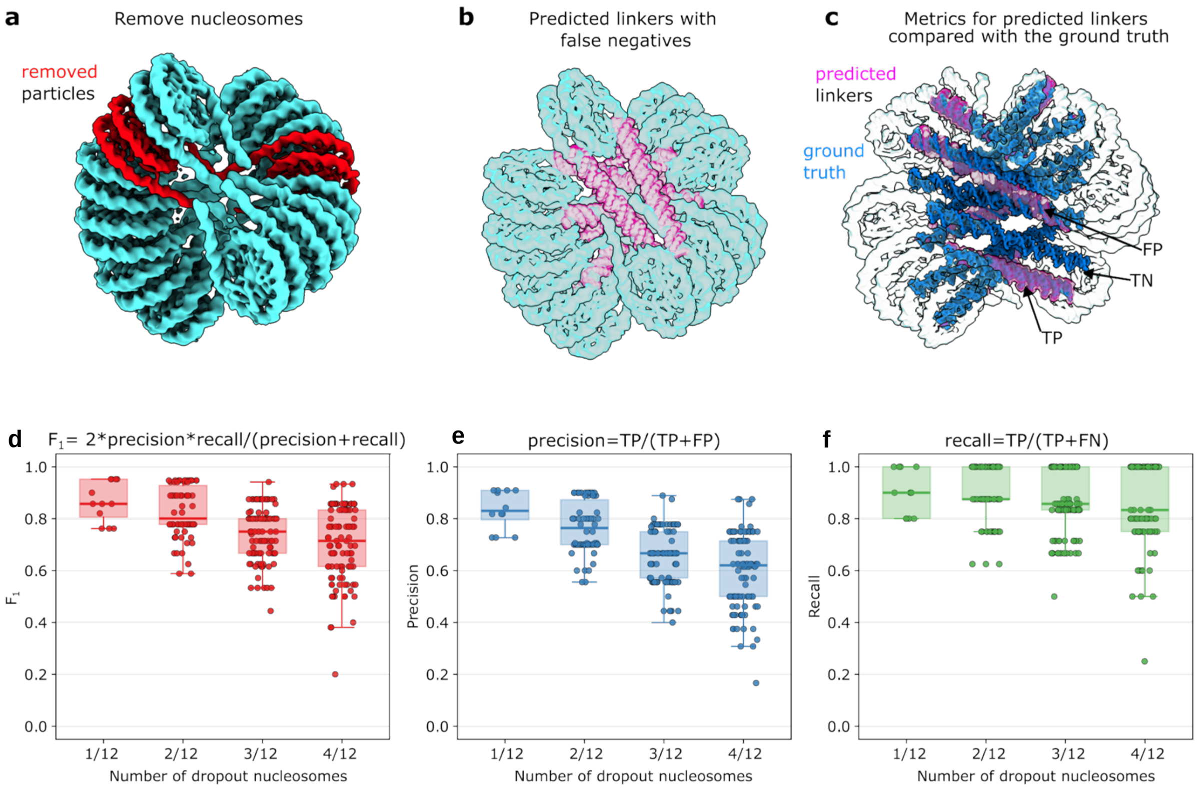

Supplementary Fig. 13. Robustness of linker prediction algorithm to false negatives. (**a**) False negative emulation by removing selected nucleosomes (red) from the list of nucleosomes fitted to the model EMD-2601[7]. (**b**) Linkers predicted from the incomplete list (magenta). (**c**) Overlay of predicted (magenta) and ground-truth linkers (blue) illustrating true positives (TP), false positives (FP) and true negatives (TN) examples. (**d–f**) Box plots of F_1 (**d**), precision (**e**), and recall (**f**) versus the number of dropped nucleosomes. For k=1,2 all possible combinations were evaluated, i.e., n=12, 66, respectively. For k= 3,4, and N=12, we used 100 random subsets of the $\binom{N}{k}$ combinations (n=100). The F1, precision, and recall scores stay above 0.5 on average even with ~1/3 of the nucleosomes removed. Source data are provided as a Source Data file.

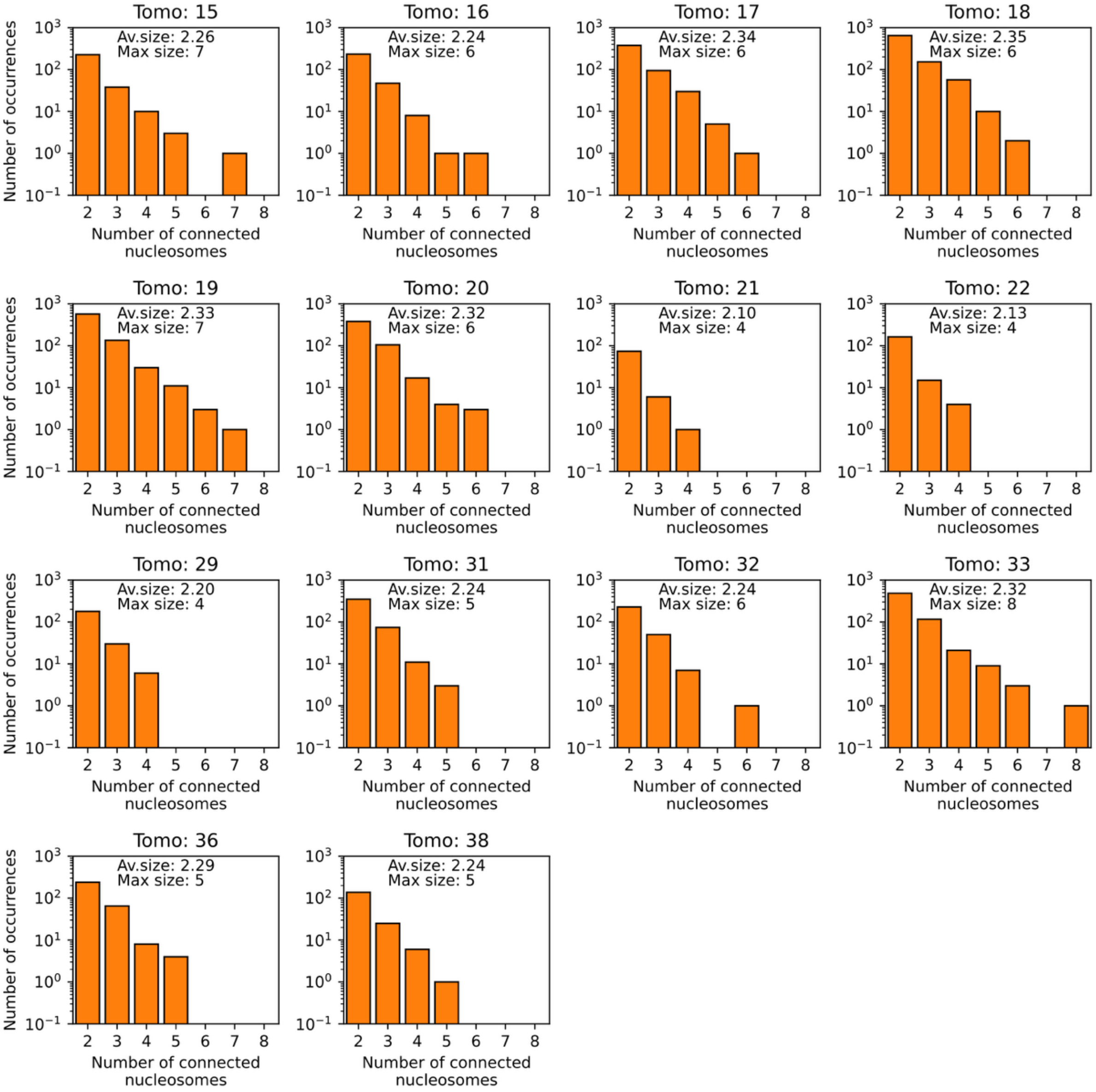

Supplementary Fig. 14. Histograms of the number of connected nucleosomes per tomogram. Distribution of the number of connected nucleosomes per tomogram after the assignment of linker connections. The inset text shows the maximum number of connected nucleosomes per tomogram, as well as the average size. Source data are provided as a Source Data file.

Supplementary Table 1: cryo-ET data acquisition parameters and STA map information.

**Microscope**	Titan Krios G4	
**Voltage (kV)**	300	
**Camera**	Falcon 4	
**Magnification**	64000	
**Acquisition pixel size (Å/px)**	1.971	
**Calibrated pixel size (Å/px)**	1.895	
**Targeted total electron dose (e^-/Å^2)**	135	
**Targeted defocus range (μm)**	-1.75 – 4.25	
**Automation software**	SerialEM	
**Tomograms used for. STA/TM**	14	
**Initial # of nucleosomes**	33560	
**Map type**	Chromatosome with C1 symmetry	Chromatosome with C2 symmetry
**Final # of particles**	33560	33560
**Resolution (Å) (FSC 0.143)**	7.4	6.8

Supplementary Movie 1.
Possible connections between nucleosomes.

Supplementary Movie 2.
Assignments of the elements in the connectivity matrix: Example of the connectivity explaining the procedure and showing that STA averages already 'draw' the linkers.

Supplementary Movie 3.
MD simulation of the 13-chromatosomes.
