# Supplementary material for: Molecular architecture of heterochromatin at the nuclear periphery of primary human cells
Source: Nat Commun. 2026 Jul 3;17:5844. doi: 10.1038/s41467-026-75087-5 (PMC13332202; doi:10.1038/s41467-026-75087-5)
Supplement: Supplementary file 2 — Description of Additional Supplementary Files [file 41467_2026_75087_MOESM2_ESM.pdf]

**Description of Additional Supplementary Files**

**Supplementary Movie 1.** Possible connections between nucleosomes.

**Supplementary Movie 2.** Assignments of the elements in the connectivity matrix: Example of the connectivity explaining the procedure and showing that STA averages already ’draw’ the linkers.

**Supplementary Movie 3.** MD simulation of the 13-chromatosomes.
