# Supplementary material for: Molecular architecture of heterochromatin at the nuclear periphery of primary human cells
Source: Nat Commun. 2026 Jul 3;17:5844. doi: 10.1038/s41467-026-75087-5 (PMC13332202; doi:10.1038/s41467-026-75087-5)
Supplement: Supplementary file 6 — Reporting Summary [file 41467_2026_75087_MOESM6_ESM.pdf]

nature portfolio

Corresponding author(s): Beata Turonová, Marina Lusic, Gerhard Hummer, Martin Beck

Last updated by author(s): May 24, 2026

Reporting Summary

Nature Portfolio wishes to improve the reproducibility of the work that we publish. This form provides structure for consistency and transparency in reporting. For further information on Nature Portfolio policies, see our Editorial Policies and the Editorial Policy Checklist.

Statistics

For all statistical analyses, confirm that the following items are present in the figure legend, table legend, main text, or Methods section.

n/a	Confirmed	
☐	☒	The exact sample size (*n*) for each experimental group/condition, given as a discrete number and unit of measurement
☐	☒	A statement on whether measurements were taken from distinct samples or whether the same sample was measured repeatedly
☒	☐	The statistical test(s) used AND whether they are one- or two-sided<br>*Only common tests should be described solely by name; describe more complex techniques in the Methods section.*
☒	☐	A description of all covariates tested
☒	☐	A description of any assumptions or corrections, such as tests of normality and adjustment for multiple comparisons
☐	☒	A full description of the statistical parameters including central tendency (e.g. means) or other basic estimates (e.g. regression coefficient) AND variation (e.g. standard deviation) or associated estimates of uncertainty (e.g. confidence intervals)
☒	☐	For null hypothesis testing, the test statistic (e.g. *F*, *t*, *r*) with confidence intervals, effect sizes, degrees of freedom and *P* value noted<br>*Give P values as exact values whenever suitable.*
☒	☐	For Bayesian analysis, information on the choice of priors and Markov chain Monte Carlo settings
☒	☐	For hierarchical and complex designs, identification of the appropriate level for tests and full reporting of outcomes
☒	☐	Estimates of effect sizes (e.g. Cohen's *d*, Pearson's *r*), indicating how they were calculated

Our web collection on statistics for biologists contains articles on many of the points above.

Software and code

Policy information about availability of computer code

Data collection	Software used for data collection: SerialEM (version 4.0.20) (see methods part of manuscript).
Data analysis	Publicly available software are described in the Methods section of the manuscript:<br>IMOD (version 4.11.5), AreTomo (version 2.0), Relion (version 3.1), novaCTF, Warp, M (version 1.0.9), GAPSTOPTM (https://gitlab.mpcdf.mpg.de/bturo/gapstop_tm), cryoCAT (https://github.com/turonova/cryoCAT/), cryoTIGER (https://github.com/turonova/cryoTIGER/), ChimeraX (version 1.8), ArtiaX (version 0.5.0), Gromacs (version 2024.2), VMD (version 1.9) HCG method

For manuscripts utilizing custom algorithms or software that are central to the research but not yet described in published literature, software must be made available to editors and reviewers. We strongly encourage code deposition in a community repository (e.g. GitHub). See the Nature Portfolio guidelines for submitting code & software for further information.

Data

Policy information about availability of data

All manuscripts must include a data availability statement. This statement should provide the following information, where applicable:

- Accession codes, unique identifiers, or web links for publicly available datasets
- A description of any restrictions on data availability
- For clinical datasets or third party data, please ensure that the statement adheres to our policy

Data availability

The chromatosome STA maps reported in this paper are deposited in the Electron Microscopy Data Bank (EMDB) with the following accession codes: EMD-58100 (chromatosome with C1 symmetry applied) [https://www.ebi.ac.uk/emdb/EMD-58100] and EMD-58101 (chromatosome with C2 symmetry applied) [https://www.ebi.ac.uk/emdb/EMD-58101].
The lower resolution STA maps used for template matching are deposited at Zenodo [https://doi.org/10.5281/zenodo.20184093].
For the T cell cryo-ET dataset, the raw frames, files for tomogram reconstruction, and 4x binned reconstructed tomograms are deposited on EMPIAR with accession code EMPIAR-13566 [https://www.ebi.ac.uk/empiar/13566].
The MD simulation data are deposited at Zenodo [https://doi.org/10.5281/zenodo.20266252].
Source data are provided with this paper

Code availability
The code for DNA linker prediction is deposited at github [https://github.com/sergiocruzleon/DNA_Linker_prediction].

Research involving human participants, their data, or biological material

Policy information about studies with human participants or human data. See also policy information about sex, gender (identity/presentation), and sexual orientation and race, ethnicity and racism.

Reporting on sex and gender	There is no sex or gender-relevant information as buffy coats were obtained from anonymous blood donors.
Reporting on race, ethnicity, or other socially relevant groupings	This manuscript does not include any research related to race, ethnicity, or other socially relevant groupings.
Population characteristics	Not relevant.
Recruitment	No recruitment was relevant.
Ethics oversight	The study was approved by Heidelberg University Medical Faculty's Ethics Committee S-604/2020 and S-025/2022. Informed written consent, according to regulations of the ethics committee, was obtained from all the anonymous blood donors in the blood bank.

Note that full information on the approval of the study protocol must also be provided in the manuscript.

Field-specific reporting

Please select the one below that is the best fit for your research. If you are not sure, read the appropriate sections before making your selection.

☒ Life sciences ☐ Behavioural & social sciences ☐ Ecological, evolutionary & environmental sciences

For a reference copy of the document with all sections, see nature.com/documents/nr-reporting-summary-flat.pdf

Life sciences study design

All studies must disclose on these points even when the disclosure is negative.

Sample size	We did not pre-determine the sample size when the study was being designed. We acquired the maximum amount of cryo-ET data from the available samples within the available microscope time.
Data exclusions	A fraction of cryo-ET data was excluded due to acquisition problems, or due to low quality leading to problems in alignment and tomogram reconstruction.
Replication	A total of 14 different tomograms from two different lamellae were independently processed and analyzed.
Randomization	We did not use randomization in our study.
Blinding	We did not use blinding in our study.

Reporting for specific materials, systems and methods

We require information from authors about some types of materials, experimental systems and methods used in many studies. Here, indicate whether each material, system or method listed is relevant to your study. If you are not sure if a list item applies to your research, read the appropriate section before selecting a response.

Materials & experimental systems

n/a	Involved in the study	
☐	☒	Antibodies
☒	☐	Eukaryotic cell lines
☒	☐	Palaeontology and archaeology
☒	☐	Animals and other organisms
☒	☐	Clinical data
☒	☐	Dual use research of concern
☒	☐	Plants

Methods

n/a	Involved in the study	
☒	☐	ChIP-seq
☒	☐	Flow cytometry
☒	☐	MRI-based neuroimaging

Antibodies

Antibodies used

Used antibodies are described in the Methods section of the manuscript:
Primary antibodies: anti–lamin B1 (Santa Cruz, sc-365962), anti–H3K27me3 (Cell Signaling Technology, 9733S), and anti–H3K4me3 (Millipore, 04-745). Secondary antibodies Sigma: ATTO 76085 anti-mouse IgG 594 and ATTO 40839 anti-rabbit IgG 647N

Validation

Primary antibodies:
1. anti–lamin B1 (Santa Cruz, sc-365962; 1:100), https://www.scbt.com/de/p/lamin-b1-antibody-c-5?srsltid=AfmBOorLjce3s5A8_tv-3pUclZ5Z_Ndx_iQIGkgRb7dk-minPAt0cBqc
2. anti–H3K27me3 (Cell Signaling Technology, 9733S; ~1:300)
https://www.cellsignal.com/products/primary-antibodies/tri-methyl-histone-h3-lys27-c36b11-rabbit-mab/9733?srsltid=AfmBOoroGtv1gF6kUAOJf0F-rgMWInFTq1OjM023UBrCkVivV0JY9NPt
3. anti–H3K4me3 (Millipore, 04-745; 1:100) https://www.merckmillipore.com/DE/de/product/Anti-trimethyl-Histone-H3-Lys4-Antibody-clone-MC315-rabbit-monoclonal,MM_NF-04-745

Secondary antibodies:
1. Sigma: ATTO 76085 anti-mouse IgG 594
https://www.sigmaaldrich.com/DE/en/product/sigma/76085?srsltid=AfmBOopwJcPaJu5SuASOxORr6Fby88wPm6ASx9Om3Xe362WEwR4IXE1I
2. Sigma: ATTO 40839 anti-rabbit IgG 647N
https://www.sigmaaldrich.com/DE/en/product/sigma/40839?srsltid=AfmBOop_p4G32OvbAuEEl6dAig4Rt0YJLVDrHzp69LFf3VEy3v9dJRNc

Plants

Seed stocks

n/a

Novel plant genotypes

n/a

Authentication

n.a
