# Supplementary material for: Molecular architecture of heterochromatin at the nuclear periphery of primary human cells
Source: Nat Commun. 2026 Jul 3;17:5844. doi: 10.1038/s41467-026-75087-5 (PMC13332202; doi:10.1038/s41467-026-75087-5)
Supplement: Supplementary file 7 — Transparent Peer Review file [file 41467_2026_75087_MOESM7_ESM.pdf]

nature portfolio

Peer Review File

Molecular architecture of heterochromatin at the nuclear periphery of primary human cells

Corresponding Author: Mr Martin Beck

This file contains all reviewer reports in order by version, followed by all author rebuttals in order by version.

Version 0:

Reviewer comments:

Reviewer #1

(Remarks to the Author)
Chromatin organization plays critical roles in genome functions such as transcription, DNA replication, and repair. Kreysing et al. determined the in-situ structures of nucleosomes in intact human resting T cells using cutting-edge cryo-ET combined with template matching, subtomogram averaging. The visualization of nucleosomes at high resolution (~6-7 Å) is very impressive: Relaxed, variable zigzag organizations of nucleosomes are connected by predicted linker DNA. The authors uncovered various higher-order chromatin structures in situ, including a 37-nm wide, elongated, but non-fibrous arrangement. This study is highly impactful for the chromatin field. I would support its publication. For Nature Communications with a broad readership, my specific comments on this short report are as follows:

Major comments:
Supplementary Figure 4. I wonder how often the authors found H1-like structures around the dyad of nucleosome (on- and off-dyad binding).

The 30-nm chromatin fiber is well described in Introduction. However, recent studies suggest that chromatin exhibits a highly variable and flexible structure rather than forming a regular 30-nm fiber (e.g., PMID: 30908980). It would be helpful to provide a more updated view of chromatin organization.

Although the authors focused on the detailed structure of chromatin, it is well known that chromatin is not static, and nucleosomes exhibit significant mobility and fluctuations in live cells, even in heterochromatin (PMID: 28712725; 32574554; 30824489; bioRxiv 2024.10.20.618801). This dynamic property directly influences chromatin organization and genome functions. A discussion of this aspect would strengthen the manuscript.

I wonder whether there are any "clutch" structures similar to those proposed in (PMID: 25768910). Since the samples in (PMID: 25768910) underwent harsh manipulation and treatment, this study could provide valuable insights into this aspect under more native conditions.

Minor points:
Ref. 39: This paper does not appear to be related to chromatin. Could this be a citation error?

Fig. 2c: Should "nuclear density" be "nucleosome density" instead? Also, are the reported density values (20–30 mg/mL) correct? Could the authors clarify what this plot represents?

Fig. 4f: "refer to the cartoon in the upper left" → "refer to Panel a"

Fig. 4g: What do "1-2-3" and "2-3-4" refer to? Are these the numbers in Panel b? Please clarify.

Line 334: Proper references should be added here, such as PMID: 30908980.

(Remarks on code availability)

Reviewer #2

(Remarks to the Author)
The authors present an interesting study investigating the 3D organization of nucleosomes in resting T-cell chromatin through an integrative approach combining cryo-electron tomography (cryo-ET), subtomogram averaging, and molecular simulations. The development of a physics-based algorithm for assigning linker DNA connections represents a notable technical contribution. While the methodology and visualizations are commendable, several methodological and interpretive aspects require clarification or additional validation to ensure the robustness of the conclusions.

Overall, this study advances the understanding of chromatin organization, and the methodological innovations are promising. Addressing the major concerns—particularly regarding algorithm validation—will strengthen the manuscript. Below, I outline specific points need to be addressed and/or further discussed.

Major points:

1. Validation of Linker DNA Assignment Accuracy
The algorithm's reliance on minimizing DNA bending energy (U_bend) and a "greedy" approach for linker DNA assignment requires stronger validation.

1.1) H1-Induced DNA Twist and Orientation Sensitivity
The current model for calculating entry/exit DNA angles does not explicitly address how linker histone (H1)-induced torsional changes (Fig. S6) affect orientation assignments. Misorientation of even a single nucleosome could propagate errors in linkage paths.

To strengthen this, please consider to
▶ Test the algorithm on in vitro chromatin fibers with defined nucleosome spacing to benchmark accuracy.
▶ Include zoomed overlays of predicted linker DNA paths on raw tomograms (e.g., Fig. S6b, 3d) to visually validate connections.

1.2) Cascade Errors from Nucleosome Detection
In dense chromatin regions, false-negative nucleosome identifications could disrupt downstream linkages. While Z-score-based particle picking is mentioned, its recall rate in crowded environments should be quantified using simulated or controlled datasets.

1.3) Resolution Limitations for Orientation Accuracy
The reported subtomogram averaging resolution (6.4–7.3 Å) raises questions about the precision of nucleosome orientation assignments, as DNA double-helical features are not resolved (Fig. 2a).
Please clarify:
▶ How orientation errors were minimized (e.g., angular tolerance thresholds).
▶ Whether resolvable nucleosome features were used to cross-validate orientations.

2. The context-Dependent Interpretation of Nucleosome Occupancy (Fig. 3d)
The reported nucleosome occupancy likely reflects local chromatin states in the sampled tomograms. Please Provide additional low-magnification tomograms (see Minor Point 5) to illustrate chromatin heterogeneity and to discuss the potential regional difference.

3. Potential Oversight of H1-Free Nucleosomes
Hou et al. (Nat. Commun., 2023) identified two nucleosome subtypes (H1-bound vs. H1-free) in T-cells. If H1-free nucleosomes exist in the current dataset, their exclusion may skew linkage analyses.
Please:
▶ Re-analyze particle classes for potential H1-free subpopulations.
▶ Discuss how H1 variability (e.g., partial occupancy) impacts linker DNA geometry.

4. Visualization of Linker DNA in Subtomogram Averages (Fig. S6)
The absence of linker DNA density in averaged maps raises concerns about assignment reliability. To clarify:
▶ Add insets showing linker DNA paths overlaid on corresponding raw tomograms.

Minor points:

1. The resolution of the nucleosome appears to be overestimated. Specifically, the characteristic features of the DNA double helix are not prominent. The DNA should exhibit clear double helix features at this resolution.

2. The molecular dynamics simulation. The MD simulations, while interesting, seem not much related to the main theme of the manuscript. In addition, it includes too many speculative claims, e.g., the H1 tails binds to distant nucleosome. It will be better if it is emphasized on how simulations complement cryo-ET findings (e.g., bending energetics).

3. Clarification of Fibrous Feature Analysis (Fig. 2f). No fibrous feature was claimed to be detected. What it would look like if the fibrous feature do exist? A simulated control would be helpful.

4. Biological Relevance of Nucleosome Stacks (Fig. S8). Are these structures transient or stabilized by specific interactions?

5. Regional Bias in Nucleosome Occupancy (Fig. 3d)
The reported nucleosome occupancy values may reflect localized chromatin states rather than global trends. Please supplement with low-magnification tomograms showing broader chromatin distribution and explicitly discuss the regional sampling difference in the Discussion.

(Remarks on code availability)

Reviewer #3

(Remarks to the Author)
The manuscript by Kreysing et al. presents a study on chromatin organization at the peripheral layer of chromatin in primary human cells using cryo-electron tomography (cryo-ET) and computational approaches. The choice of experimental system is relevant, because indeed dormant T-cells contain have large amount of heterochromatin. The authors interpret the organization of chromatin fibers at the nuclear periphery by identifying nucleosome positions and orientations within the tomograms and predicting DNA linkers based on their most probable directions.

The authors successfully obtained a very high-resolution sub-tomogram average of the nucleosome structure in situ, highlighting their expertise in cryo-ET data processing. They present a single average corresponding to a chromatosome - a nucleosome with a bound histone H1 at the nucleosome dyad region. Does this imply that the chromatosome (and its minor conformational variants shown in Supplementary Fig. 4) represents the exclusive conformation of nucleosomes in the analyzed chromatin? If thoroughly justified, this would be a significant biological finding, especially on a background of the high dynamics of nucleosomes and histone H1, as well as the short residence time of the latter in its bound state (see https://doi.org/10.1101/2025.03.05.641622., and citations therein).

The primary focus of the manuscript is the prediction of DNA linkers based on nucleosome-picking results. However, the novelty of this approach may be limited, as linker DNA prediction for human T-lymphoblasts has already been reported (https://doi.org/10.1038/s41467-023-42072-1), and direct visualization and tracing of DNA linkers have been demonstrated in Drosophila (https://doi.org/10.1101/2022.08.16.502515). Nevertheless, in the context of human cells, the authors introduce a physical parameters based DNA path prediction, which represents advancement.

My key concern is that the linker prediction relies solely on nucleosome positions and orientations determined by computational tools, under the assumption that all nucleosomes adopt a single chromatosome conformation. For this approach to yield reliable results, a thorough justification of the angular accuracy and exhaustiveness of nucleosome annotations is necessary. The visibility of linker arms in the average of 35K particles is insufficient to support claims regarding their absolute stiffness. It is also essential to assess the exhaustiveness of nucleosome annotation, as this directly impacts the reliability of linker path prediction when individual DNA linkers are not visible. This is particularly crucial for predicting and interpreting long-range nucleosome connections.

Specific comments:
1. The use of the word ‘tracing’ for DNA linkers in the abstract and later in the manuscript is misleading. The authors present a ‘prediction’ of the linker or an ‘estimate’ of the linker trajectory (as they say in the discussion).

2. It is unclear how heterochromatin is defined. Do the authors mean only the 50 nm thick boundary layer adjacent to the nuclear envelope, or the entire volume of tomograms, or are they based on the density of nucleosomes? In the latter case, what does euchromatin look like? Can it be separated? No clear estimate of the extent of heterochromatin from the nuclear envelope is given. In my opinion, the attribution of the observed peripheral regions to heterochromatin seems hypothetical without precise mapping. This could be done by CLEM localization of heterochromatin-associated proteins such as HP1 and PRC, for example. The claim that the observed regions represent exclusively heterochromatin requires further evidence.

3. The authors used a template-matching approach to identify nucleosomes. Detailed explanations are required to assess its effectiveness and the absence of a putative bias towards a single nucleosome conformation. The information provided by the authors is indeed insufficient: ‘a limited amount of debris was found’. How was the classification carried out? The classes shown in Supplementary Figure 4 appear to be the result of post-alignment classification, which might be biased towards the original averaging. Was any initial reference-free classification, for example multiclass classification-alignment procedure in Relion, performed after every two rounds of template matching to assess the conformational variability of nucleosomes?

An arbitrary threshold was set by the authors for template matching. How exhaustive was the resulted annotation of nucleosomes? Did you test automatic against manual expert annotation on a representative volume? It is known that template matching can also bias the orientation of nucleosomes towards side views that have higher contrast. The angular distribution of selected nucleosomes should be presented.

4. The term “Multi-nucleosome arrangement” introduced by authors is difficult to define. “Multy-” would fit to anything starting from nano-domains to chromosome territories.

5. Chromatin is very dynamic even in very compact states in vivo. The lowest energy predictions would correspond to the equilibrium state that is very unlikely for the dynamic native chromatin. It might result in bias towards straight linkers.

To my understanding, the connectivity model and physics-based prediction of the linker path assume absolute precision in the positions and orientations of the picked nucleosomes, which may not be entirely realistic. How robust is the model to potential translational (1–4 pixels) and orientation noise (e.g., 10–30°)?

The MD simulation movie shows very limited nucleosome movements without H1 association/dissociation. How realistic are the binding stability and time of H1?

6. Line 55. “stiff linkers” statement is wrong. Stiffness is a relative concept: compare the stiffness of DNA and actin filaments. The Luger et al. citation does not apply to DNA linkers.
7. Line 63. H1 histones have general affinity to DNA and can bind everywhere to chromatin indeed in vivo, not only in the relation to the DNA entry/exit regions.
8. Lines 87-90. The zigzag topology of chromatin in situ was directly identified previously (https://doi.org/10.1101/2022.08.16.502515).
9. Line 124 and the further statements about C2 symmetry. The chromatosome is not a completely symmetrical structure. Can you explain how symmetry was applied to it?
10. Line 392. The term ‘vitrification’ should not be used if cell vitrification is not justified. I cannot judge as access to the raw tilt series was not provided.
11. Supplementary Figure 6b. It is visually difficult to relate the density seen in the images to DNA. Where are the nucleosomes located? I assume they must be resolved as well. Why are only the YZ and XZ species shown? Is the scale bar 2 nm? Is the citation 49 correct?

(Remarks on code availability)

Version 1:

Reviewer comments:

Reviewer #1

(Remarks to the Author)
The authors have adequately addressed my previous comments in the revised manuscript. I am satisfied with the revisions, and I recommend the manuscript for publication.

(Remarks on code availability)

Reviewer #2

(Remarks to the Author)
I appreciate the authors’ efforts in addressing the previous comments and further validating their method using in vitro chromatin map. The study presents a potentially powerful approach for analyzing chromatin organization. However, several conceptual and methodological issues remain to be clarified, and some results require further interpretation before the manuscript can be considered for publication.
My main comments are as follows:
1. The reported overall resolution does not represent the accuracy of each individual particle. The reported high resolutions do not guarantee that each particle in the dataset is aligned with high accuracy. In practice, many particles—especially those with low SNR or suffering from ion-beam–induced damage—may be poorly aligned or even appear false positives. Although the overall resolution may remain high due to the majority of good particles, per-particle alignment uncertainty can significantly affect downstream analyses, particularly the linker DNA prediction, which depends on precise nucleosome positioning.
I strongly recommend that the authors establish a method to quantify the alignment precision for each individual particle, rather than relying solely on the global resolution estimate. This would also help explain the observed reduction in recall for linker prediction when alignment inaccuracies occur. If this is technically infeasible, the authors should at least discuss this potential source of error explicitly and avoid assuming that a high-resolution average map implies uniformly accurate per-particle alignment.
2. It should be clarified whether the nucleosomes used for linker prediction were all obtained from the STA process or whether template-matching (TM) particles were also included. Figure 3 shows both STA and TM. If TM-derived nucleosomes were used, and if their orientations can be accurately determined, then it should be discussed why STA is necessary at all. Alternatively, if the method is robust to moderate angular noise, the authors might justify using TM-only data

for prediction, demonstrating the claimed robustness of the approach.
3. Regarding the H1-free category, in Supplementary Figure 5, the right-hand averaged nucleosome clearly shows weak or missing H1 density, suggesting the loss or flexibility of H1. Moreover, the DNA trajectory in these cases does not appear to follow an H1-constrained path. Even in heterochromatin regions, the presence of H1 cannot be assumed to be homogeneous. The authors should discuss this heterogeneity explicitly and reconsider their interpretation of the “H1-free” population.
4. Although low-magnification tomograms do not show individual nucleosomes clearly, they are essential for assessing the overall chromatin compaction state. The authors should provide representative low-magnification maps for multiple lamellae to demonstrate that the observed chromatin looseness is a general feature rather than a selection bias from the 14 tomograms.
5. It remains unclear why certain nucleosomes are incorrectly connected, even under in vitro conditions where the nucleosome orientation appear correct. The authors are encouraged to identify potential patterns or contributing factors that lead to incorrect connections. Including such a discussion would strengthen the methodological insights of the manuscript.
6. The statement that the predicted linkers “require significant bending” seems conceptually inaccurate. The in vitro chromatin map is all straight DNA. The authors should revisit this interpretation and clarify whether these cases represent algorithmic artifacts or structural deviations.
7. The claim that the nucleosomes are “off-dyad” is not well supported. The current resolution and alignment precision are insufficient to make such an assignment. The apparent H1 density appears more consistent with an “on-dyad” position. The authors could consider to fit the density to a PDB model and provide quantitative evidence supporting this claim.
8. It would greatly enhance the manuscript’s impact to include a specific example demonstrating what can be done with the reconstructed DNA. This would illustrate the practical utility and broader relevance of the method.

(Remarks on code availability)

Reviewer #3

(Remarks to the Author)
The authors have substantially improved the manuscript, addressing most of the previous comments with careful revisions and additional analyses. The quality and clarity of the work have significantly increased. However, two important methodological issues and one terminology-related concern remain that require further clarification or adjustment.

1. Preferential orientation pattern of the angular distribution of picked nucleosomes

The authors have presented the angular distribution of the picked nucleosomes, which reveals a clear bias toward certain orientations. I disagree with the authors’ formulation that “the angular orientation of the nucleosomes was slightly biased towards top views.” This statement does not accurately reflect the situation shown in Letter Figure 3, which reveals a pronounced preferential orientation pattern, visible as distinct angular segments, very similar to those reported by Kelley et al. (https://doi.org/10.1101/2024.12.28.630444) indeed. The distribution indicates that a relatively narrow range of orientations contains a disproportionately high number of picked nucleosomes.
The angular distribution figure should be added to the Supplementary Information in two views (similar to Kelley et al., Supplementary Figure 8G), to give readers the opportunity to recognize this important limitation.

This observation also has important implications:

(i) The presence of angular sectors with severalfold higher particle counts demonstrates that nucleosome picking was far from exhaustive. Consequently, the measured nucleosome density derived from this picking underestimates the actual nucleosome density. This bias may affect relative density comparisons at local scales around 30-50 nm, because chromatin fiber orientation might influence its picking efficiency. For example, a segment of compact chromatin fiber oriented close to the z-axis would likely contain more top views of nucleosomes, which have a lower probability of being picked and might be misinterpreted as chromatin-free fraction. To improve understanding, it would be useful to compare the spatial distribution of nucleosomes in preferred orientations with one of all other orientations.
Furthermore, the orientation bias will influence the global nucleosome density values reported. I suggest that the authors estimate and apply a correction factor by comparing the frequency of nucleosomes picked at preferred orientations with ones of the low picking frequency. Provided that the corrected nucleosome density values remain physically consistent with the spatial constraints of the analyzed chromatin domains, this adjustment would yield a more realistic estimate of nucleosome concentration. If it leads to unrealistically high densities, this would indicate the presence of false positives or a non-uniform orientation of chromatin fibers within the tomogram(s). The latter, although unlikely, would still be important to investigate.

(ii) The presence of preferred orientations will likely influence particle classification. Because of the missing wedge, a sufficiently high population of nucleosomes whose superhelical axes are parallel or nearly parallel to the z-axis of the reconstruction is essential to discriminate between chromatosomes and nucleosomes with more distant entry and exit DNA segments, characteristic of the H1-free state. A dominance of side views among the picked nucleosomes could therefore hinder the detection of an H1-free class. Since the conformational uniformity of nucleosomes as chromatosomes is a key foundation of the linker prediction in this study, it would be important to explore this potential bias. For example, this could be done by balancing the angular distribution of nucleosomes, by reducing the number of particles picked in preferred orientations, and rerunning the classification from scratch, aiming not for a high resolution, but for exploring conformational diversity.

The results of nucleosome picking and classification in Chlamydomonas reinhardtii reported by Kelley et al. (https://doi.org/10.1101/2024.12.28.630444) support this concern. As shown in Supplementary Figure 8D of that work, the initial classification also revealed mainly chromatosome-like conformations. However, since the picking in that study covered the entire nucleus of Chlamydomonas reinhardtii, which mainly contains open euchromatin, it is unlikely that majority of nucleosomes were chromatosomes.

2. Validation of prediction robustness

"Reply: The reviewer raises an interesting point, namely how robust is our algorithm to uncertainties in the positions and orientations of the nucleosomes. This point has also been raised by other reviewers. See reply to comment 1 of Reviewer 2, and the new Supplementary Figure 11.
We explicitly assessed the robustness of our predictions by adding controlled noise to nucleosome coordinates and Euler angles in a challenging, dense, and well-characterized in vitro structure (see new Supplementary Figure 11). The angular and positional accuracies of nucleosome orientations come from Relion 3D refinement, with estimated uncertainties of ~1.6° and ~1.8 Å, respectively. As shown in Supplementary Figure 11e,f, linker predictions remain stable, with high F1 scores and precision up to perturbations far exceeding these reported uncertainties."

The estimated uncertainties provided by Relion represent general statistical values and are not necessarily precise for each particle in noisy data. Therefore, a detailed assessment of the robustness of the linker prediction to variable rotational and translational noise levels would be essential for the study.
To my understanding, all robustness checks were performed only on the 12-mer array EMDB-2601. This is a highly compact structure consisting of three nucleosome tetramers with constant linker length and a repetitive geometry of linkers. It has very limited geometrical freedom and thus does not represent the geometrical diversity of chromatin in situ. I therefore disagree that this structure constitutes a representative in vitro model for testing prediction robustness.

Tomographic datasets presented in the work of Jentink et al. (https://doi.org/10.1016/j.molcel.2023.08.017)) may provide a more relevant and realistic in vitro testing system. These annotated datasets of native isolated human chromatin fragments are publicly available (https://datadryad.org/dataset/doi:10.5061/dryad.ttdz08m21)).

Specific comments:
1. Supplementary Figure 11: It is not clear how rotational noise with σ = 10°–30° was applied to the tightly packed EMDB-2601 structure without causing steric clashes between neighboring nucleosomes. It should by also clarified whether this σ value applies to each rotational angle.
2. A statistical comparison should be provided for the charts shown in Supplementary Figures 11 and 12.

Terminology clarification: word "tracing"

"Reply: We understand the reviewer's point. Although tracing is defined as 'to follow an already defined path', which we think is what we do, our algorithm does not take into account the actually observed electron optical density between nucleosomes, e.g. by segmentation.
We want to stress that we very often have used the term 'predicted linkers' in the manuscript that makes the predictive character of our algorithm perfectly transparent. We nevertheless carefully reconsidered and adjusted our phrasing throughout. We could not entirely eliminate the word trace, because it is also used in the methods part for patches of chromatin (not the linkers), where we think it is appropriate because it indeed refers to connections between confidently identified elements (the nucleosomes). Here, we added this statement:
"We use the verb 'trace' here to describe the connection of nucleosomes into patches of chromatin by interpolating the DNA linkers in-between them."

The authors' interpretation of the term "tracing" appears to correspond to "prediction of the DNA linker" or "interpolation." Moreover, their explanatory statement clarifying this usage is provided only on page 24 within the Methods section. Presenting this term without clarification in the abstract and introduction is potentially misleading for a general audience, as it can be understood as implying direct detection of cryo-EM density for all reported linkers. For the sake of precision and scientific rigor, the term "tracing" should be replaced in the abstract with "physics-based prediction" or "interpolation," and the explanatory statement should be introduced before its first use in the main text. Such a change would not diminish the impact of the study but would instead enhance its clarity and accuracy.

Minor comments:

3. The templates used for the "no H1 included" state classification are not cited. Please indicate the corresponding PDB entry.

4. Supplementary Figure 10: The tomographic slices shown in the left panel are very difficult to interpret. It is nearly impossible to recognize individual nucleosomes, especially for readers outside the cryo-EM field, likely due to excessive denoising. I suggest including corresponding isosurface views to facilitate visual interpretation.

(Remarks on code availability)

Version 2:

Reviewer comments:

Reviewer #2

(Remarks to the Author)
The authors have addressed most of my comments.

(Remarks on code availability)

Reviewer #3

(Remarks to the Author)
I am satisfied with the revisions and recommend the manuscript for publication.

(Remarks on code availability)

Open Access This Peer Review File is licensed under a Creative Commons Attribution 4.0 International License, which permits use, sharing, adaptation, distribution and reproduction in any medium or format, as long as you give appropriate credit to the original author(s) and the source, provide a link to the Creative Commons license, and indicate if changes were made.
In cases where reviewers are anonymous, credit should be given to 'Anonymous Referee' and the source.
The images or other third party material in this Peer Review File are included in the article's Creative Commons license, unless indicated otherwise in a credit line to the material. If material is not included in the article's Creative Commons license and your intended use is not permitted by statutory regulation or exceeds the permitted use, you will need to obtain permission directly from the copyright holder.
To view a copy of this license, visit https://creativecommons.org/licenses/by/4.0/

We thank all three reviewers for their thoughtful and constructive feedback, which has been very helpful to improve our manuscript.

Our major changes are summarized here:

1. The reviewers asked about the abundance of H1-free nucleosomes in our data, and questioned if they were indeed not observed.

   We included additional extensive image classification analysis, in which we specifically targeted H1-free nucleosomes. This analysis did not converge into any decently resolved H1-free structures, but on the contrary, rather recovered features typical for H1-containing nucleosomes. We interpret this finding such that the vast majority of nucleosomes in out tomograms are H1-containing.

   Related to that, the reviewers asked how H1-free nucleosomes with their less restrained linker arms, would influence the performance of our algorithm.

   We note that although our algorithm uses the linker arm orientation of H1 containing nucleosomes as a starting point, it identifies considerable deviations from it, in average of about 15 degrees.

2. The reviewers asked how we justify our classification of the observed state of chromatin as heterochromatin (which contains a higher number of H1-containing nucleosomes).

   We included additional data showing that within the given field of view (up to 400 nm away from the nuclear envelope), heterochromatin markers are highly enriched, while markers for euchromatin are less abundant.

3. The reviewers requested additional analysis that validates our algorithm for the prediction of linkers between nucleosomes against a known ground truth.

   We included additional validation of our algorithm, using in vitro data with a known ground truth. We demonstrate that our algorithm operates with very good accuracy.

   The reviewers further have asked if our algorithm may propagate errors once it may have made a false assignment.

   We thoroughly checked this point and provide statistical support that this is not the case. We note that we focus on high confidence identification. The price to pay for this is that our assignments only sparsely cover chromatin. As an

upside, errors that may occur can only locally propagate but not affect the majority of the other (disconnected) assignments.

At last, due to a now more accurate calibration of the pixel size of our microscope at the given magnification, some numbers have slightly shifted. This regards primarily the Fourier shell correlation plots in the revised Supplementary Figure 2.

Our detailed point by point response explaining the changes that have been made to the manuscript follows below.

Reviewer Comments:

Reviewer #1 (Remarks to the Author):

Chromatin organization plays critical roles in genome functions such as transcription, DNA replication, and repair. Kreysing et al. determined the in-situ structures of nucleosomes in intact human resting T cells using cutting-edge cryo-ET combined with template matching, subtomogram averaging. The visualization of nucleosomes at high resolution (~6-7 Å) is very impressive: Relaxed, variable zigzag organizations of nucleosomes are connected by predicted linker DNA. The authors uncovered various higher-order chromatin structures in situ, including a 37-nm wide, elongated, but non-fibrous arrangement. This study is highly impactful for the chromatin field. I would support its publication. For Nature Communications with a broad readership, my specific comments on this short report are as follows:

Major comments:
Supplementary Figure 4. I wonder how often the authors found H1-like structures around the dyad of nucleosome (on- and off-dyad binding).

Reply: This is certainly a very interesting question that we also thought about; unfortunately we do not have a conclusive answer. We can only say that we have identified on and off dyad species, which at least suggests that they are prominently represented in the data (new Supplementary Figure 6).

More specifically, and in contrast to our previous work, e.g. on ribosomes and TRiC, in which we have used class averaging to quantify the abundance of the identified functional states, the 3D classification of nucleosomes is less consistent across multiple classification runs. This does not affect the types of averages that one obtains, but rather the number of particles contained in each class. We attribute this to the comparably small size of the nucleosomes. We very actively work on a solution, but have not yet achieved stable classifications. Therefore, we

avoid stressing the apparent sizes of classes as a metric of quantification of functional states.

The 30-nm chromatin fiber is well described in Introduction. However, recent studies suggest that chromatin exhibits a highly variable and flexible structure rather than forming a regular 30-nm fiber (e.g., PMID: 30908980). It would be helpful to provide a more updated view of chromatin organization.

Reply: We thank the reviewer for this clarification and have made appropriate textual changes in the introduction.

> p4. "However, recent studies have suggested that chromatin can be highly dynamic and variable inside cells questioning the physiological relevance of a static 30 nm fiber[11]"

Although the authors focused on the detailed structure of chromatin, it is well known that chromatin is not static, and nucleosomes exhibit significant mobility and fluctuations in live cells, even in heterochromatin (PMID: 28712725; 32574554; 30824489; bioRxiv 2024.10.20.618801). This dynamic property directly influences chromatin organization and genome functions. A discussion of this aspect would strengthen the manuscript.

Reply: We agree that chromatin is not a static structure. We have added to the discussion to highlight this limitation of our study.

> p16. "One limitation of our cryo-ET study is the lack of dynamics we can observe. Chromatin is known to be a dynamic structure albeit less so in heterochromatic regions[46-49]. Each tomogram can therefore be seen as one chromatin state frozen in time out of many possible configurations."

I wonder whether there are any "clutch" structures similar to those proposed in (PMID: 25768910). Since the samples in (PMID: 25768910) underwent harsh manipulation and treatment, this study could provide valuable insights into this aspect under more native conditions.

Reply: This is another interesting question. We at least do not frequently observe the discrete nanodomains coined clutches, as observed with STORM by the study the reviewer mentioned. We believe that our density-based clustering analysis should have detected such features, if they were consistent and abundant. Without strong statistical evidence, we can state that the nucleosome arrangement showcased in Figure 1b, c is subjectively observed multiple times; it may be considered consistent with the median number of 8 nucleosomes per clutch (PMID: 25768910).

Minor points:
Ref. 39: This paper does not appear to be related to chromatin. Could this be a citation error?

Reply: Yes, the reference has been corrected.

Fig. 2c: Should "nuclear density" be "nucleosome density" instead? Also, are the reported density values (20–30 mg/mL) correct? Could the authors clarify what this plot represents?

Reply: We have updated the label to make clear that it represents the 'number of detected nucleosomes per μm³'. Labels in Fig. 2a,e now read: 'local density of detected nucleosomes (mg/ml)'

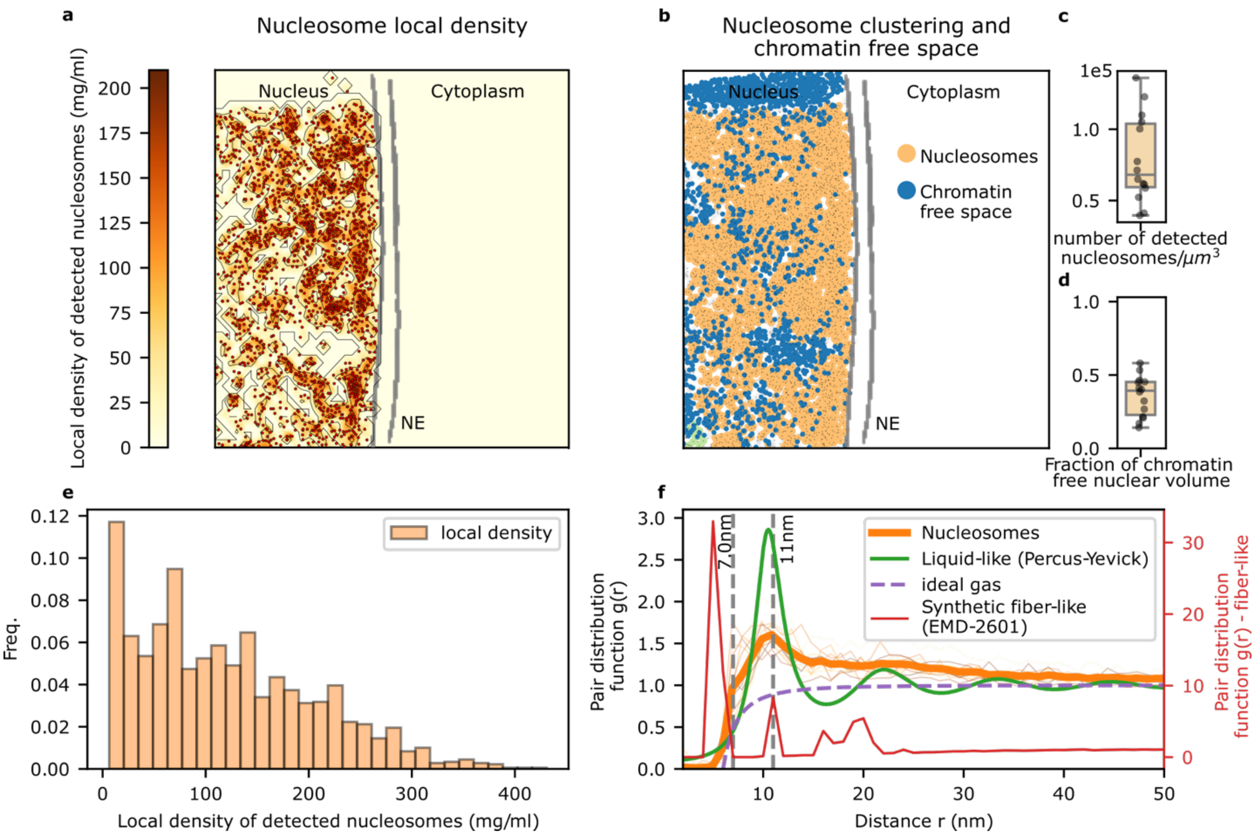

Fig 2: Spatial properties of heterochromatin at nuclear periphery of T cells. **a** Nucleosomes local density. **b** Hierarchical density-based clustering (orange and green clusters) and chromatin free space visualized from the same exemplifying tomogram as in Fig 1a. **c** Overall number of detected nucleosomes per unit volume. **d** Fraction of chromatin free nuclear volume. **e** Local density of detected tomograms; distribution over all tomograms. **f** Pair distribution function g(r) of the nucleosomes. Reference curves for the ideal gas and liquid-like distributions[32], and a synthetic tomogram made of 30nm-fibers segments (EMD-2601[7]) are included for comparison. Gray dashed lines are the peaks of nucleosomes g(r).

Fig. 4f: "refer to the cartoon in the upper left" → "refer to Panel a"

Reply: The text has been changed accordingly.

Fig. 4g: What do "1-2-3" and "2-3-4" refer to? Are these the numbers in Panel b? Please clarify.

Reply: Yes, these labels refer to the numbers in panel Figure 4b, and now has been specified in the legend:

> "Magenta points are the values for the sequence of numbered nucleosomes from panel b."

Line 334: Proper references should be added here, such as PMID: 30908980.

Reply: The suggested reference has been added.

Reviewer #2 (Remarks to the Author):

The authors present an interesting study investigating the 3D organization of nucleosomes in resting T-cell chromatin through an integrative approach combining cryo-electron tomography (cryo-ET), subtomogram averaging, and molecular simulations. The development of a physics-based algorithm for assigning linker DNA connections represents a notable technical contribution. While the methodology and visualizations are commendable, several methodological and interpretive aspects require clarification or additional validation to ensure the robustness of the conclusions.

Overall, this study advances the understanding of chromatin organization, and the methodological innovations are promising. Addressing the major concerns—particularly regarding algorithm validation—will strengthen the manuscript. Below, I outline specific points need to be addressed and/or further discussed.

Major points:

1. Validation of Linker DNA Assignment Accuracy
The algorithm's reliance on minimizing DNA bending energy (U_bend) and a "greedy" approach for linker DNA assignment requires stronger validation.

1.1) H1-Induced DNA Twist and Orientation Sensitivity

The current model for calculating entry/exit DNA angles does not explicitly address how linker histone (H1)-induced torsional changes (Fig. S6) affect orientation assignments. Misorientation of even a single nucleosome could propagate errors in linkage paths.

Reply: We hope we understood the reviewer correctly that they are asking how changes in the torsional angle of both linker arms of a given nucleosome—as induced, e.g., by binding of H1—may affect the accuracy of our algorithm and potentially result in the propagation of errors, if one nucleosome is misassigned. We therefore believe the reviewer is referring to the previous Supplementary Fig 4 (now Supplementary Fig 6) (H1-bound classes) and not Supplementary Fig 6 (now Supplementary Fig 9) (subtomogram averaging of linkers).

As we now show in the revised version, the vast majority of nucleosomes seems to contain H1; thus, we cannot claim that torsional changes are H1-induced (Supplementary Fig 5).

Classes of H1-bound nucleosomes (now Supplementary Fig 6) indicate only very small variations in the torsional angle, at least for the vast majority of nucleosomes that converges into those high-resolution averages. We of course cannot exclude that individual nucleosomes may have larger torsional angles of the linker arms; however, the respective population cannot be large.

In terms of nucleosome linker coverage, our algorithm yields rather sparse results, at this stage, and it makes assignments in local nucleosome clusters. If an individual nucleosome were misassigned, the respective error would only locally propagate but not globally within an entire tomogram.

To strengthen this, please consider to
▶️ Test the algorithm on in vitro chromatin fibers with defined nucleosome spacing to benchmark accuracy.

Reply: We thank the reviewer for this suggestion. As recommended, we benchmarked our algorithm on a set of in vitro chromatin reconstructions with defined nucleosome spacing and known geometry to assess its accuracy and robustness (see new Supplementary Figs 11,12).

Specifically, we applied the linker prediction to the 3-mers EMD-13370 and EMD-13363, the 4-mer EMD-13356, and the more compact and crowded 12-mer EMD-2601. These structures span a range of linker lengths, curvatures, and packing densities. We located each nucleosome by cross-correlation and used these rotations and translations for the prediction of the linkers. Despite the local

complexity, our algorithm recovers the correct connectivity with high accuracy (New Supplementary Figure 11a-d). Linker lengths deviate by only ~3 bp from the expected values.

Furthermore, as suggested by Reviewer 3, we assessed the robustness by systematically perturbing the nucleosome coordinates and orientations in the 12-mer. Specifically, we added isotropic Gaussian noise to the particle coordinates from EMD-2601. Positional, and angular noise was independently swept in the range [0,30] Å and [0, 30] deg, respectively.

Predictions for F1 score, precision, and recall under increasing levels of angular and positional noise are shown in the new Supplementary Fig. 11e,f. The algorithm maintains high accuracy within the range of typical alignment uncertainty (RELION estimates: ~1.6 Å, ~1.8°), indicating reliable performance.

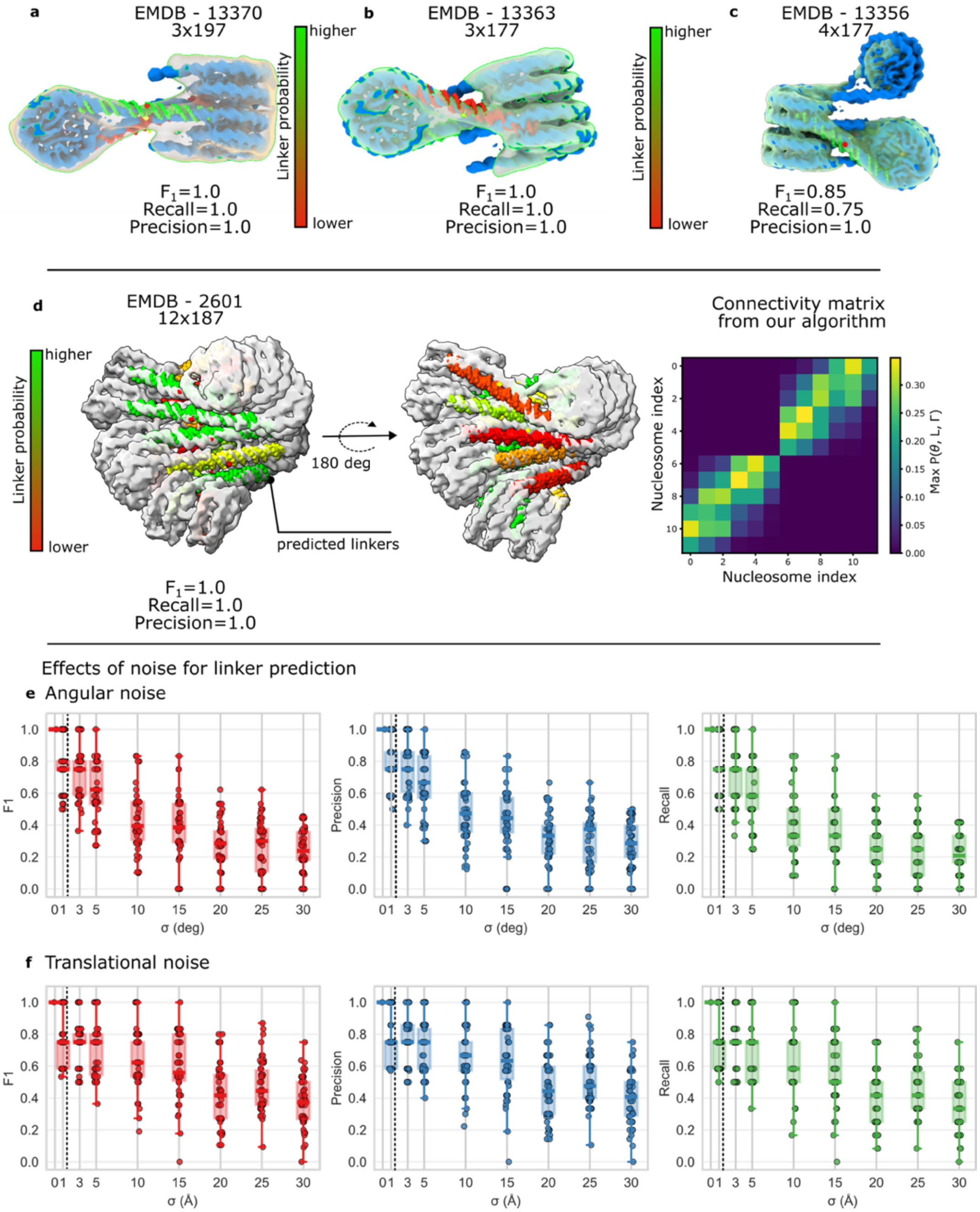

Supplementary Figure 11: Validation and robustness analysis of the DNA linker prediction algorithm on in vitro chromatin reconstructions. Validation and robustness analysis of the DNA linker prediction algorithm on in vitro chromatin reconstructions. (a-c) Predicted linkers for three in vitro chromatin assemblies (EMD-13370[6] EMD-13363[6], EMD-13356[6]) shown overlaid on their EM density maps. Linkers are colored by probability (green = higher, red = lower). (d) Example prediction on a compact 12-mer (EMD-2601[7]), with linker probabilities mapped onto the density and corresponding connectivity matrix (Pmax) for the 12-mer. Note that predicted linkers depicted in red require a significant bending. (e–f) Robustness analysis of the linker predictions under increasing levels of angular (e) and translational (f) noise (σ = 0°–30°), and (σ = 0–30 Å), respectively. We report F1 score, recall, and precision. Each data point represents one of 50 independent runs per noise level (n=50); box plots show median, interquartile range, and outliers.

▶️ Include zoomed overlays of predicted linker DNA paths on raw tomograms (e.g., Fig. S6b, 3d) to visually validate connections.

Reply: We thank the reviewer for this suggestion. We have implemented this as suggested (see below and new Supplementary Fig. 10). This visualization certainly does further strengthen the confidence in our predicted linkers. We want to stress, however, that a disadvantage of this representation is that many of the linkers are slightly bent, and therefore not captured in full in a planar tomographic slice.

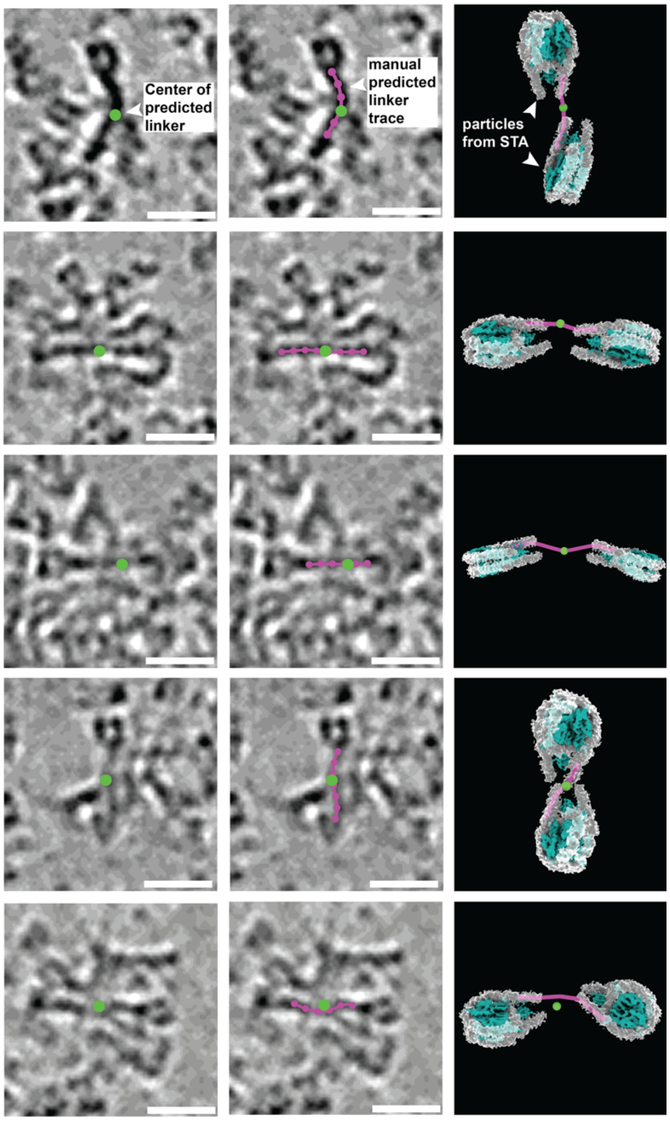

Supplementary Figure 10: Overlay of predicted DNA linker positions on tomographic slices. From left to right for five predicted linkers: (leftmost) Tomographic slice showing a DNA linker density and connected nucleosomes with a green circle indicating the center of the predicted linker. (middle) Same tomographic slice with manually traced DNA linker to illustrate plausible path in pink. (rightmost) Visualization of the nucleosomes (positions and orientations obtained through STA, DNA in transparent grey and histones in teal) and a manually placed linker in pink. Note: The center of the fifth predicted linker is slightly shifted compared to the visually traceable linker density. Scale bar in all slices indicates 20 nm. All tomograms were denoised with cryoCARE[30].

1.2) Cascade Errors from Nucleosome Detection
In dense chromatin regions, false-negative nucleosome identifications could disrupt downstream linkages. While Z-score-based particle picking is mentioned, its recall rate in crowded environments should be quantified using simulated or controlled datasets.

Reply: The reviewer brings up an interesting point that is recurrent in tomographic analysis, namely the false negatives in the detection of particles. However, since the ground truth for experimental tomograms remains unknown, we cannot directly address the question of false-negative identifications.

To address this important point raised by the reviewer, we tested our method on in vitro data with known ground truth, including a compact 12-mer (EMD-2601). Although nucleosomes in this data set are extremely crowded, we found that our algorithm achieves high precision and robust F1 scores (new Supplementary Figures 10 and 11).

We agree that false negatives in nucleosome detection may affect downstream connectivity assignments. To address this, we have performed ‘leave one out’ analysis with the aforementioned in vitro data. This analysis suggests that our linker tracing is resilient to occasional missed nucleosomes, e.g. the F1 score drops to 75% if 4 out of 12 nucleosomes are missed (see below and new Supplementary Figure 12).

We note that our algorithm focuses on pairwise connections of nucleosomes that were identified with high-confidence. The results are sparse, but come with the benefit of lower false-positive rates.

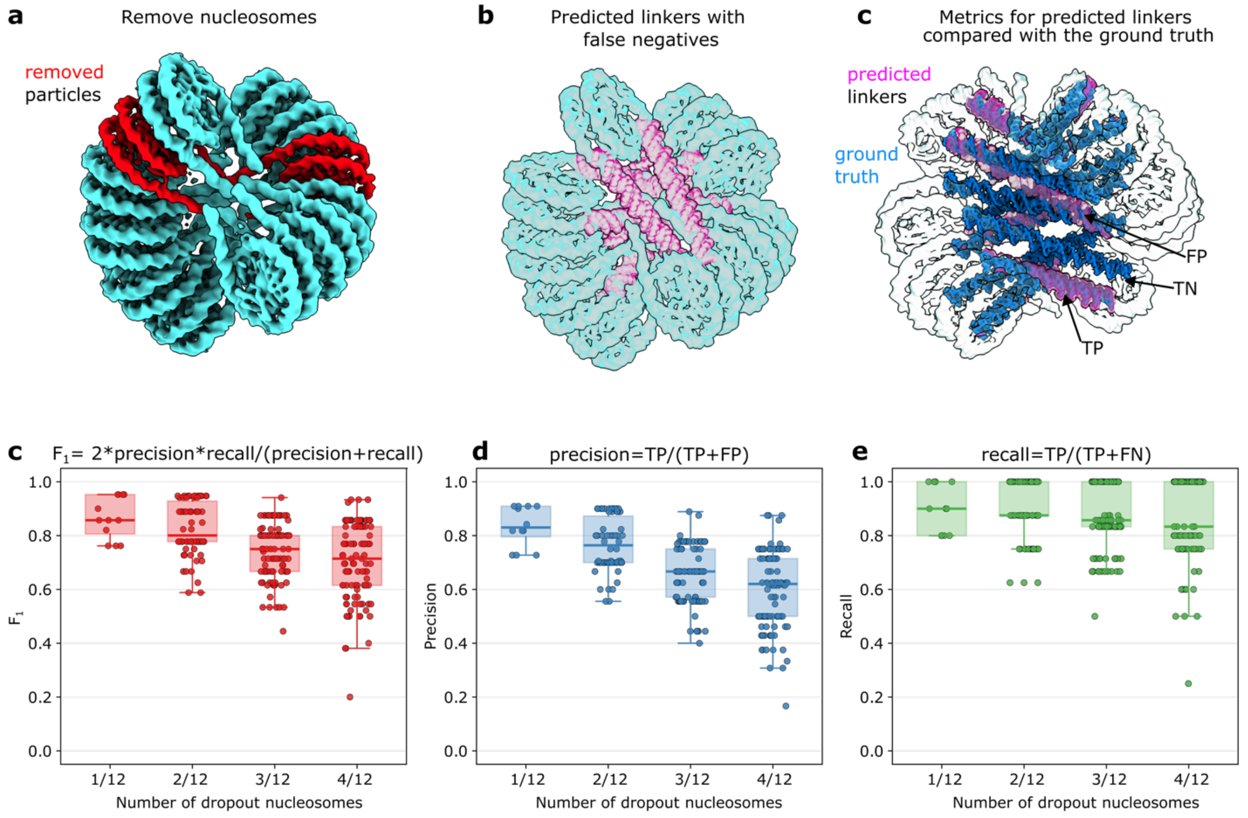

Supplementary Figure 12: Robustness of linker prediction algorithm to false negatives. (a) False negative emulation by removing selected nucleosomes (red) from the list of nucleosomes fitted to the model EMD-2601[7]. (**b**) Linkers predicted from the incomplete list (magenta). (**c**) Overlay of predicted (magenta) and ground-truth linkers (blue) illustrating true positives (TP), false positives (FP) and true negatives (TN) examples. (**c–e**) Box plots of F_1 (**c**), precision (**d**), and recall I versus the number of dropped nucleosomes. For k=1,2 all possible combinations where evaluated, i.e., n=12, 66, respectively. For k=3,4 and N=12, we used 100 random subsets of the $\binom{N}{k}$ combinations (n=100).

1.3) Resolution Limitations for Orientation Accuracy
The reported subtomogram averaging resolution (6.4–7.3 Å) raises questions about the precision of nucleosome orientation assignments, as DNA double-helical features are not resolved (Fig. 2a).
Please clarify:
▶️ How orientation errors were minimized (e.g., angular tolerance thresholds).
▶️ Whether resolvable nucleosome features were used to cross-validate orientations.

Reply: We respectfully disagree with the assessment that DNA double-helical features are not resolved. The major and minor groove are clearly resolved, although their position is only partially consistent with the published high-resolution structures containing the ‘Widom’ nucleosome positioning sequence. Interestingly,

the resolution obtained for the DNA is lower as compared to the histones, where the secondary structure features are very obvious. This may indicate dynamics of the DNA structure and sequence, which is also apparent when class averages are compared.

To make this clearer, we have improved our visualization of the chromatosome structure in Figure 1g to point out where the DNA double-helical features such as the major and minor groove are visible. In Figure 1e,f we now point out that we can resolve parts of the histone tails, more specifically the N-terminal tail of H3 and H2B. We have also created a ChimeraX session for the reviewers to look at the map with a fitted chromatosome model.
Link: https://oc.biophys.mpg.de/owncloud/s/82dSz5nLZzf9iNd
Password: chromatosome25cET

The subtomogram alignment and averaging was performed using Relion3 with the Refine3D routine, which iteratively changes the angular search parameters based on the current resolution. We have included an additional figure to this letter (Letter Figure 1) to show how the alignment converges. Relion estimates the angular accuracy to be 1.8 degrees and the positional accuracy to be 1.6 Å.

We are unsure what exactly the reviewer means when mentioning 'whether resolvable nucleosome features were used to cross-validate orientations'. We want to point out that although in single particle EM, 2D class averages represent orientations, classes in subtomogram averaging do always contain particles in various orientations.

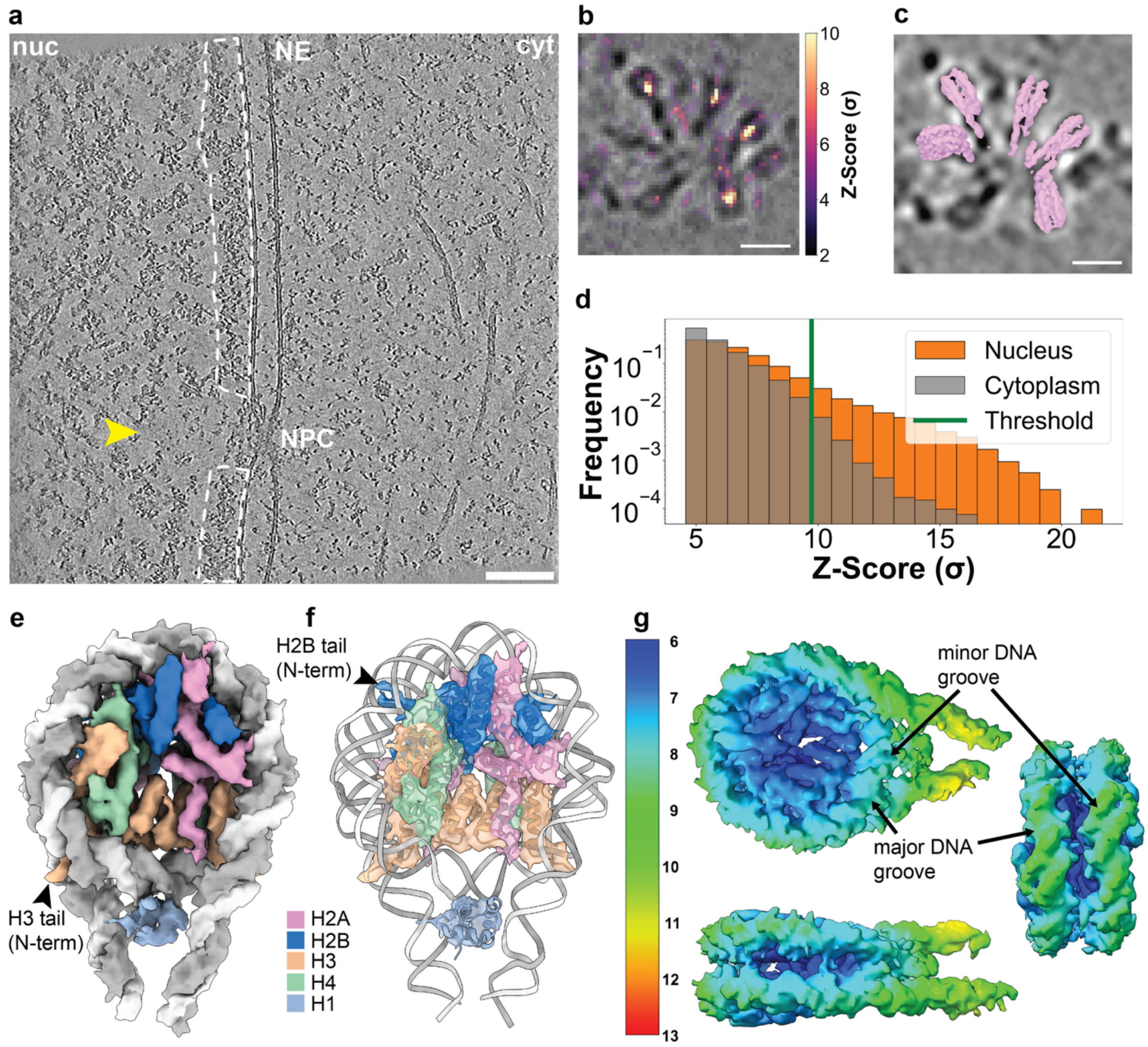

Fig 1: **In situ structural analysis identifies nucleosomes inside of cells and resolves their secondary structure. a** Slice through a cryoCARE-denoised tomogram acquired at the nuclear periphery of resting T cells. Chromatin-free space is indicated with a yellow arrow. A dense layer of chromatin underlying the nuclear envelope is framed white. **b** A local feature of multiple nucleosomes superimposed with the respective cross-correlation function obtained by template matching. **c** The same local feature but with the identified nucleosomes shown as isosurfaces. **d** Histogram of Z-scores for the cytosolic and nucleoplasmic region for exemplary tomogram. **e,f** STA map of the human chromatosome, color-coded by DNA strands (white and gray) and histone proteins H2A (pink), H2B (blue), H3 (amber), H4 (green) and H1 (light blue). The atomic model of the canonical chromatosome (PDB: 7DBP 27) was fitted into our map. The partially resolved N-terminal tails of the H2B and H3 histones are highlighted with arrows **g** Color-coded local resolution STA map of the human chromatosome (from high resolution in blue to low resolution in red, in Å). The major and minor DNA groove are highlighted with arrows. Scale bars 100 nm in **a** and 10 nm in **b,c**.

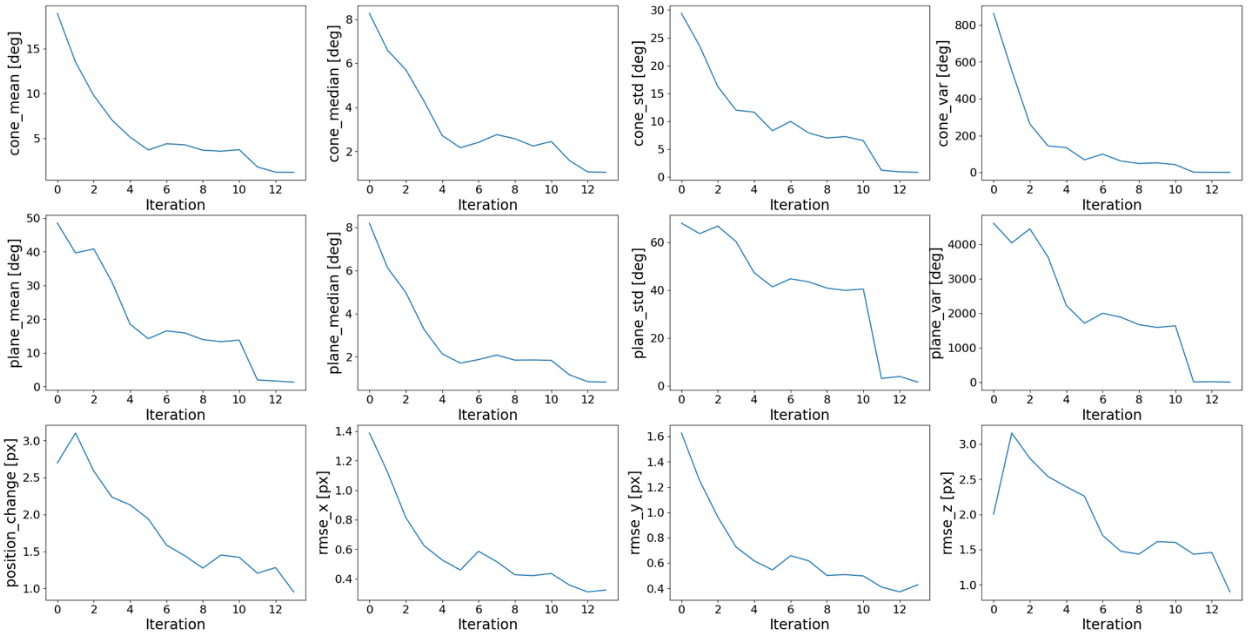

Letter Figure 1: STA converges after multiple rounds of refinement. The changes in nucleosome particle orientations and positions as determined by the Relion STA procedure are shown for each iteration of refinement until it converged after 14 iterations. The orientations of the nucleosome particles in Euler angles are shown split into cone and in-plane rotations. For each of the two categories the mean, median, standard deviation and variance of the differences between iterations is plotted. For the positions, both the pairwise distance as well as the root mean square errors of each of the three coordinates (x,y,z) are plotted.

2. The context-Dependent Interpretation of Nucleosome Occupancy (Fig. 3d)
The reported nucleosome occupancy likely reflects local chromatin states in the sampled tomograms. Please Provide additional low-magnification tomograms (see Minor Point 5) to illustrate chromatin heterogeneity and to discuss the potential regional difference.

Reply: We agree that our tomograms capture the situation at the nuclear periphery, and cannot account for chromatin states that occur deeper in the nucleus. We have deliberately chosen higher magnification for this project, because it is required to confidently detect nucleosomes. Therefore, we do not think that such data could address the point of the reviewer about heterogeneity.

Nevertheless, to make this more transparent, we acquired fluorescence light microscopy data in which we quantified the distance of typical markers for heterochromatin and euchromatin from the nuclear envelope (see below). More specifically, heterochromatin typically extends about 0.5 micron into the nucleus. The chromatin region covered in the field of view of our tomograms, however, reaches only up to 400 nm beyond the nuclear envelope (compare to Figure 1a). These data underscore our interpretation that our tomograms contain primarily heterochromatin.

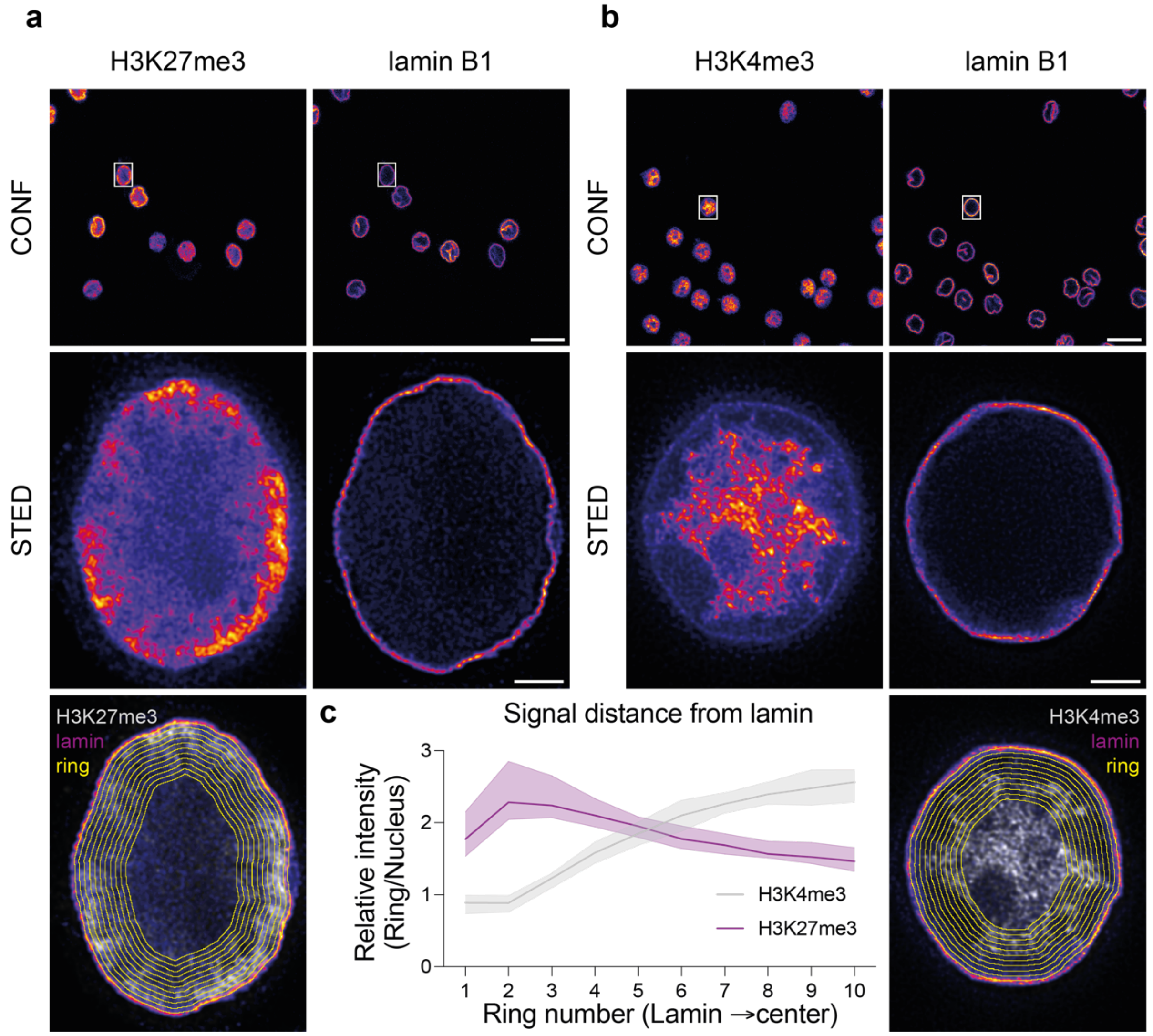

Supplementary Figure 1. STED imaging of chromatin markers in resting CD4+ T cells and quantitative analysis. Fire LUTs of lamin B1 and H3K27me3 (**a**) or H3K4me3 (**b**) signals. The top panels show a broad confocal view (CONF, scale bar = 10 μm), the middle panels a deconvoluted STED acquisition of a central Z-plane enlargement (STED, scale bar = 1 μm). The bottom panels display the channel overlap, with fire LUT for lamin B1, gray LUT for the histone mark, and ten concentric rings of 100 nm width generated from the lamin signal (yellow). (**c**) Quantification of signal intensity (mean gray values) measured in the indicated rings and normalized to the mean signal of the entire nucleus. The distance between rings is 100 nm. Graphs represent the median (solid line) and interquartile range (shaded area) of pooled cells from two independent blood donors (violet: H3K27me3, n = 8 and 7 cells from each donor; gray: H3K4me3, n = 12 and 10 cells).

3. Potential Oversight of H1-Free Nucleosomes

Hou et al. (Nat. Commun., 2023) identified two nucleosome subtypes (H1-bound vs. H1-free) in T-cells. If H1-free nucleosomes exist in the current dataset, their exclusion may skew linkage analyses.

Please:

▶️ Re-analyze particle classes for potential H1-free subpopulations.

▶️ Discuss how H1 variability (e.g., partial occupancy) impacts linker DNA geometry.

Reply: We followed the reviewer’s suggestion and reanalyzed the data. Even extensive classification efforts did not converge into decently resolved subtomogram averages of H1-free nucleosomes (see methods and attached Supplementary Figure 5). Contrary to that, even when H1-free references were used for classification, the resulting averages were recovering H1 density, at least in part.

Although we cannot formally exclude an H1-free subpopulation in our data, we think it is fair to state that the vast majority of nucleosomes appear to be H1-bound. We also would like to note that the cells in which Hou et al. detected a minor fraction of H1-free nucleosomes (albeit at lower resolution), were immortalized human T-lymphoblast CEM cells grown in tissue culture, which are not directly comparable to our healthy blood donor-derived primary CD4+ T cell samples.

We have added the following remark to the limitations of the study section:

> p17. “In this study, the majority of the detected nucleosomes appears to be H1-bound. We based this interpretation on the observation that extensive classification efforts did not result in highly resolved subtomogram averages of H1-free nucleosomes (Supplementary Fig 5). However, we cannot fully exclude the possibility that the performance of our algorithm is negatively influenced by the presence of H1-free nucleosomes with more flexible linker arms, even if they are not prominent in our samples.”

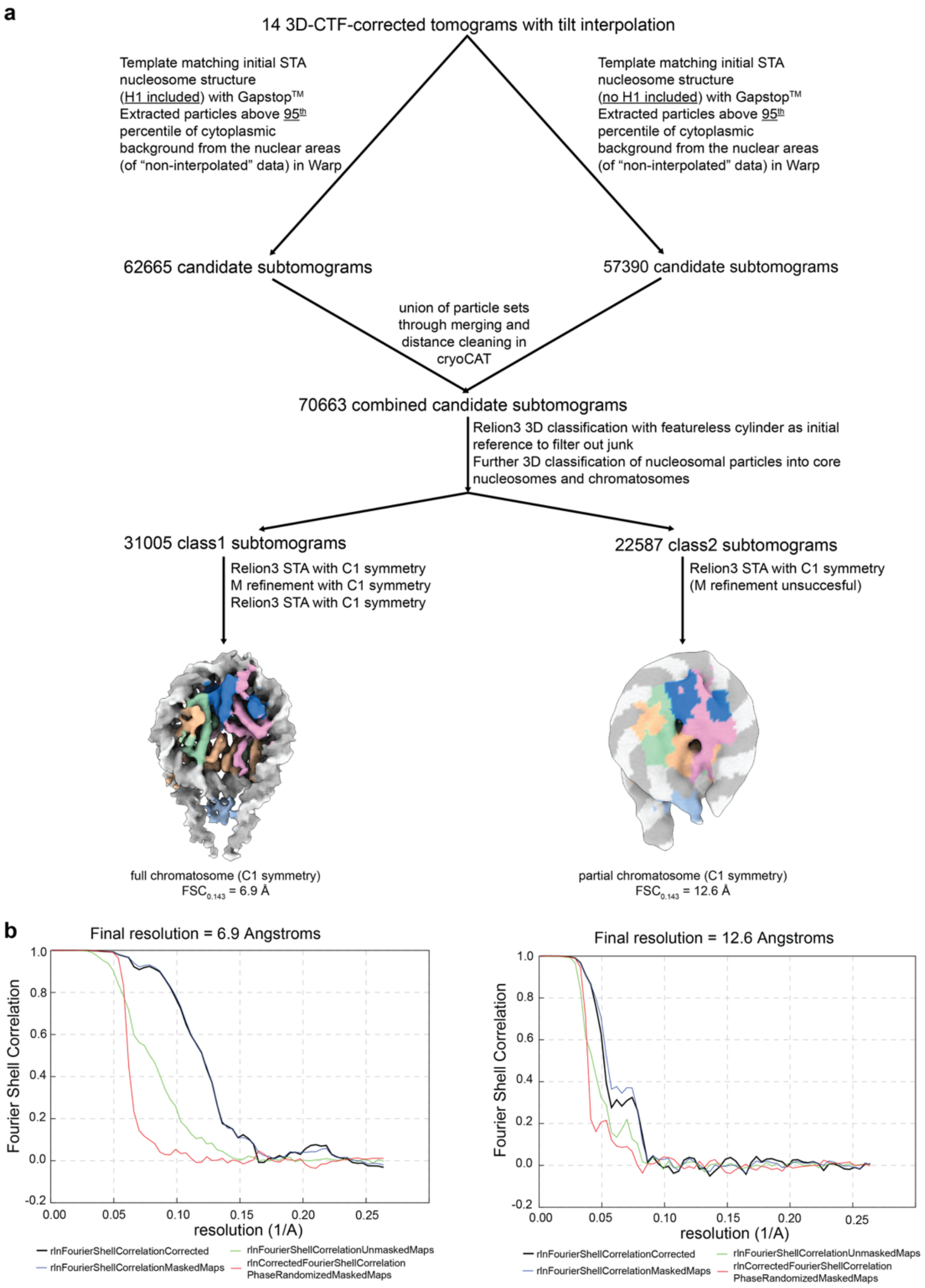

Supplementary Figure 5. Processing workflow for extensive 3D classifications to find H1-free nucleosome class. a Workflow describing the steps taken to classify a H1-free nucleosome population. **b** Fourier shell correlation curves obtained from Relion 3.1[34]. for full and partial chromatosome averages

4. Visualization of Linker DNA in Subtomogram Averages (Fig. S6)

The absence of linker DNA density in averaged maps raises concerns about assignment reliability. To clarify:

▶️ Add insets showing linker DNA paths overlaid on corresponding raw tomograms.

Reply: We first want to clarify that both, averages of nucleosomes (Supplementary Figure S6) and of linkers (Supplementary Figure S9), do contain density for the linker DNA. However, it gradually becomes less apparent with increasing linker length, because the degrees of freedom for bending into different directions and with different angles increases. As a consequence, the respective density is diluted out during averaging.

We have added a figure depicting predicted linkers overlayed with the corresponding tomograms (Supplementary Figure 10) as requested. We want to point out that a shortcoming of this representations is that bent linkers cannot be captured in a planar tomographic slice.

Minor points:

1. The resolution of the nucleosome appears to be overestimated. Specifically, the characteristic features of the DNA double helix are not prominent. The DNA should exhibit clear double helix features at this resolution.

Reply: We respectfully disagree with the reviewer's opinion that the resolution of our chromatosome structure is overestimated. The local resolution mapping indicates that the DNA itself is not resolved to 6-7 Å like the nucleosome core, but rather 8-10 Å. To make this clearer, we have revised the text as follows:

> p.6: We obtained an in situ structure of the chromatosome from resting T cells locally resolved to 6.0 Å in the nucleosome core, 8-10 Å in the DNA (6.8 Å overall) with imposed C2 symmetry (Fig 1e-g).

As mentioned in response to reviewer #2's point 1.3 we also improved our visualization of the chromatosome structure in Figure 2g to point out where the DNA double-helical features such as the major and minor groove are visible. From a 3D inspection of the map the DNA features become more evident. We have created a ChimeraX session for the reviewers to look at the map with a fitted chromatosome model.
Link: https://oc.biophys.mpg.de/owncloud/s/82dSz5nLZzf9iNd
Password: chromatosome25cET

We want to point out that in contrast to *in vitro* nucleosome structures that usually rely on one defined positioning sequence of DNA, we are averaging nucleosomes in situ with no defined DNA sequence. It is therefore to be expected that the DNA has lower resolution than the histone proteins (Figure 1g).

This drop in resolution from the histone core to the DNA is also common to single-particle cryo-EM structures of nucleosomes.

2. The molecular dynamics simulation. The MD simulations, while interesting, seem not much related to the main theme of the manuscript. In addition, it includes too many speculative claims, e.g., the H1 tails binds to distant nucleosome. It will be better if it is emphasized on how simulations complement cryo-ET findings (e.g., bending energetics).

Reply: We are convinced, that the MD simulations add an important dimension to the manuscript, because they are geometrically based on the experimental data, because they are higher resolution, and because they are dynamic. Regarding the example the reviewer mentions, the simulations do not proof that H1 tails bind to distant nucleosomes in the cell, but they emphasize that in a typical nucleosome arrangement that was tomographically identified, this is a real possibility, and important to consider. We have rephrased this passage as follows:

> "In particular, we observe the H1 tails binding to distant nucleosome cores and linker DNAs, promoting chromatin compaction (Supplementary Movie 3). Although we cannot prove that such interactions occur in cells, this observation illustrates that in a tomographically verified nucleosome arrangement such interactions have to be considered as a possible interaction."

More generally, we realize that the complementary nature of simulations and experiments could be emphasized further, in particular with respect to linker bending.

To address this, we have added a visualization of linker bending from MD simulations (revised Supplementary Figure 9c) and connect it to our observations from STA and the linker prediction algorithm. We observe changes in the angle of the linkers in a tri-nucleosome of about 10° across the trajectory (Figs. 4f, 5b), which is consistent with the experimental observations. Nucleosome linkers display significant flexibility (revised Supplementary Figure 9c), which is with our linker prediction model and agrees with the lack of well-resolved linker density in STA at longer predicted lengths (revised Supplementary Figure 9d).

> p. 14: We observe changes in the angle of a tri-nucleosome of about 10° across the trajectory (Figs. 4f, 5b). Linker DNAs are correspondingly flexible (Supplementary Fig 6), in agreement with the assumptions of the linker prediction algorithm and the lack of longer DNA linkers in our chromatosome STA (Fig 1g).

We further added the details of linker bending in the simulations to the Methods section:

> p.28: To illustrate the dynamics of the system, we drew DNA base pair traces every 5 ns starting at 10 ns by aligning the central nucleosome of a trimer with the xy-plane and calculating the center of mass of complementary base pairs across the trajectory. For visualization we rendered the trimer at 70 ns (Fig. 5b). To illustrate linker dynamics, we traced the duplex helical axis between nucleosomes 4 and 5 (see Fig. 5a in the main text) after aligning on the two connected nucleosomes. Frames extracted every 5 ns (Supplementary Figure 9c).

3. Clarification of Fibrous Feature Analysis (Fig. 2f). No fibrous feature was claimed to be detected. What it would look like if the fibrous feature do exist? A simulated control would be helpful.

Reply: We thank the reviewer for this suggestion. A simulated control has been added to Fig. 2f; it displays the characteristic peaks expected for regularly spaced 30 nm-fiber segments, whereas the experimental RDF does not show these patterns. Thus, it further supports the absence of ordered fibers. The figure caption has been updated, and a brief description of the simulation is now included in the Methods (page ABC):

> p.22: "To mimic a random fiber-like arrangement, the 12-particle coordinate set describing one 30 nm-fiber segment from the map EMD-2601 was used as a rigid template. 6000 copies were placed in a 0.7 µm³ box with random orientations and translations while rejecting clashes. From this list of particles, the radial distribution function g(r) was obtained as described above."

4. Biological Relevance of Nucleosome Stacks (Fig. S8). Are these structures transient or stabilized by specific interactions?

Reply: While our cryo-ET data, as a single snapshot of the cell, cannot directly address the temporal dynamics of nucleosome stacks, Supplementary Figure 7 shows that such stacks occur in specific conformations. Furthermore, our simulations suggest that stacking is stabilized by specific molecular interactions (primarily H4 tail-mediated contacts, as previously shown in e.g. doi.org/10.1016/j.jmb.2017.03.016). The literature indicates that these structures exist in a dynamic equilibrium between stacked and unstacked states (doi.org/10.1038/s41467-017-02619-5).

However, any further-reaching interpretation about the interactions and temporal dynamics of which would be speculative.

5. Regional Bias in Nucleosome Occupancy (Fig. 3d)
The reported nucleosome occupancy values may reflect localized chromatin states rather than global trends. Please supplement with low-magnification tomograms showing broader chromatin distribution and explicitly discuss the regional sampling difference in the Discussion.

Reply: We agree that additional information about the distribution of chromatin within the T cells with respect to the cellular position of our tomograms would strengthen our arguments. We however, did not acquire any low magnification tomograms, because we know that nucleosomes cannot be confidently identified at such large pixel sizes.

Instead, we acquired fluorescence light microscopy data in which we quantified the distance of typical markers for heterochromatin and euchromatin from the nuclear envelope (see below). More specifically, heterochromatin typically extends about 0.5 micron into the nucleus. The chromatin region covered in the field of view of our tomograms, however, reaches only up to 400 nm beyond the nuclear envelope (compare to Figure 1a). These data underscore our interpretation that our tomograms contain primarily heterochromatin.

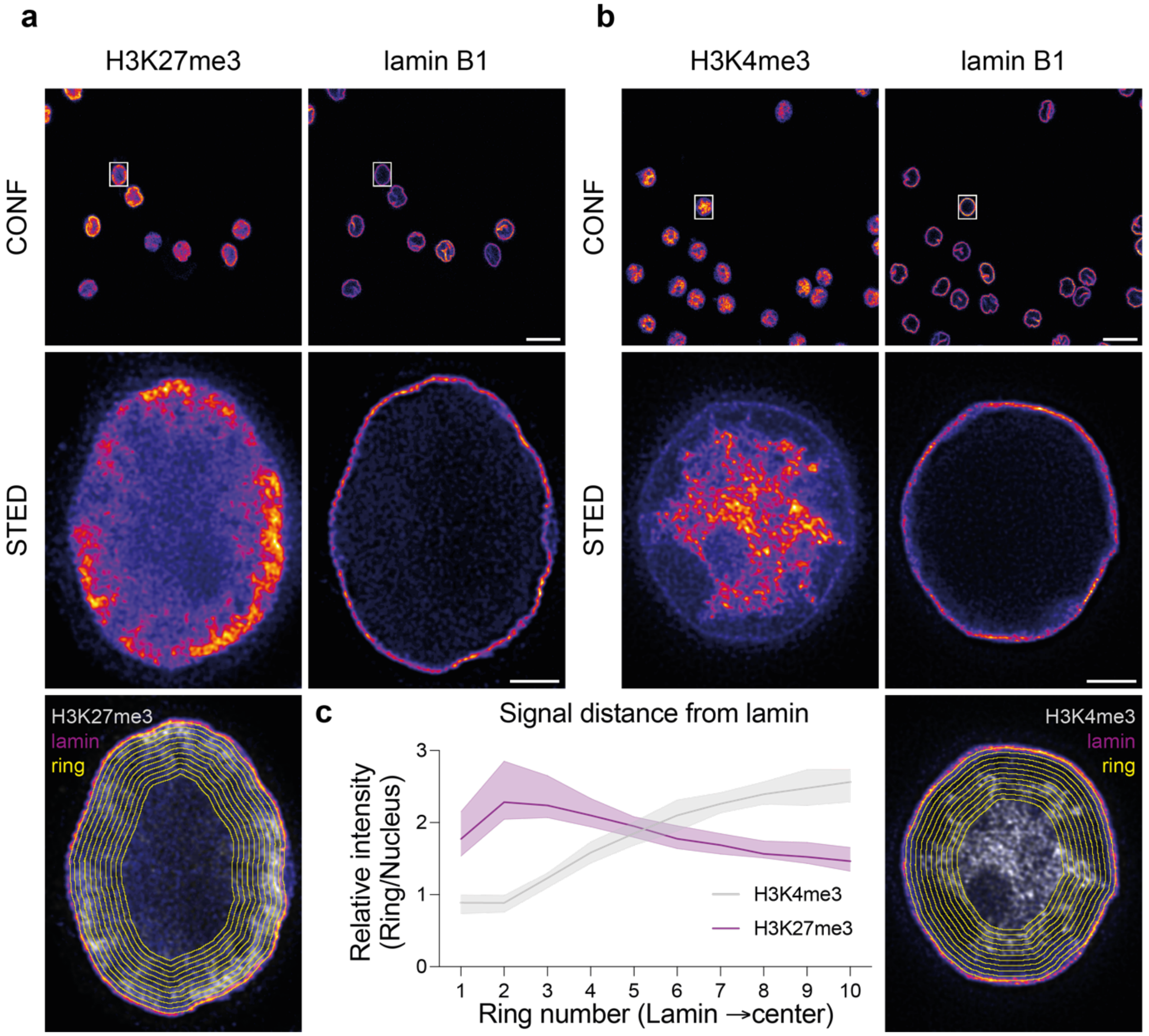

Supplementary Figure 1. STED imaging of chromatin markers in resting CD4+ T cells and quantitative analysis. Fire LUTs of lamin B1 and H3K27me3 (**a**) or H3K4me3 (**b**) signals. The top panels show a broad confocal view (CONF, scale bar = 10 µm), the middle panels a deconvoluted STED acquisition of a central Z-plane enlargement (STED, scale bar = 1 µm). The bottom panels display the channel overlap, with fire LUT for lamin B1, gray LUT for the histone mark, and ten concentric rings of 100 nm width generated from the lamin signal (yellow). (**c**) Quantification of signal intensity (mean gray values) measured in the indicated rings and normalized to the mean signal of the entire nucleus. The distance between rings is 100 nm. Graphs represent the median (solid line) and interquartile range (shaded area) of pooled cells from two independent blood donors (violet: H3K27me3, n = 8 and 7 cells from each donor; gray: H3K4me3, n = 12 and 10 cells).

Reviewer #3 (Remarks to the Author):

The manuscript by Kreysing et al. presents a study on chromatin organization at the peripheral layer of chromatin in primary human cells using cryo-electron tomography (cryo-ET) and computational approaches. The choice of experimental system is relevant, because indeed dormant T-cells contain have large amount of heterochromatin. The authors interpret the organization of chromatin fibers at the nuclear periphery by identifying nucleosome positions and orientations within the tomograms and predicting DNA linkers based on their most probable directions.

The authors successfully obtained a very high-resolution sub-tomogram average of the nucleosome structure in situ, highlighting their expertise in cryo-ET data processing. They present a single average corresponding to a chromatosome - a nucleosome with a bound histone H1 at the nucleosome dyad region. Does this imply that the chromatosome (and its minor conformational variants shown in Supplementary Fig. 4) represents the exclusive conformation of nucleosomes in the analyzed chromatin? If thoroughly justified, this would be a significant biological finding, especially on a background of the high dynamics of nucleosomes and histone H1, as well as the short residence time of the latter in its bound state (see https://doi.org/10.1101/2025.03.05.641622., and citations therein).

Reply: We thank the reviewer for the appreciation of our work. This point was also raised the other reviewers. Therefore, we analyzed our entire dataset again to specifically search for H1-free nucleosomes, but unsuccessfully. Even extensive classification using an H1-free reference, did not result in subtomogram averages that would converge to decent resolution. Instead, the averages recovered H1, at least in part (see response to reviewer #2's point 3 and Supplementary Fig 5). We identified 5 classes of containing H1 in slightly different structural states. Although it is always difficult to argue with absence of evidence, we interpret this data such that at least the majority of nucleosomes we analyzed at the nuclear periphery should contain H1. We mention this in the limitations of the study section as follows:

> "In this study, the majority of the detected nucleosomes appears to be H1-bound. We based this interpretation on the observation that extensive classification efforts did not result in highly resolved subtomogram averages of H1-free nucleosomes (Supplementary Fig. 5). Therefore, we cannot exclude that the performance of our algorithm is negatively influence by the presence of H1-free nucleosomes with more flexible linker arms, even if they were not prominent in our samples."

The primary focus of the manuscript is the prediction of DNA linkers based on nucleosome-picking results. However, the novelty of this approach may be limited, as linker DNA prediction for human T-lymphoblasts has already been reported (https://doi.org/10.1038/s41467-023-42072-1), and direct visualization and tracing of DNA linkers have been demonstrated in Drosophila (https://doi.org/10.1101/2022.08.16.502515). Nevertheless, in the context of human cells, the authors introduce a physical parameters based DNA path prediction, which represents advancement.

Reply: We thank the reviewer and agree that previous work has established the feasibility of DNA linker identification. Here, we develop a computational method that is fully automated and physics-based, allowing large-scale linker prediction directly from nucleosome positions and orientations. In contrast, prior work has relied on intuition or manual approaches, which are low-throughput and labor-intensive.

To illustrate the magnitude of the task at hand, we want to highlight that our workflow allowed us to generate a much larger dataset of linkers (7163 linkers in total) as compared to the manual tracing used in the Drosophila (140 linkers in total, from Supplementary Table 1, doi.org/10.1101/2022.08.16.502515).

This scale enables, for the first time, statistically robust analysis of chromatin architecture inside human cells. Furthermore, by incorporating DNA bending energetics, our approach improves the physical plausibility of predicted linkers beyond proximity-based criteria.

My key concern is that the linker prediction relies solely on nucleosome positions and orientations determined by computational tools, under the assumption that all nucleosomes adopt a single chromatosome conformation. For this approach to yield reliable results, a thorough justification of the angular accuracy and exhaustiveness of nucleosome annotations is necessary. The visibility of linker arms in the average of 35K particles is insufficient to support claims regarding their absolute stiffness. It is also essential to assess the exhaustiveness of nucleosome annotation, as this directly impacts the reliability of linker path prediction when individual DNA linkers are not visible. This is particularly crucial for predicting and interpreting long-range nucleosome connections.

Reply: We thank the reviewer for these comments regarding the use of the chromatosome positions and orientations, angular accuracy, and exhaustiveness of nucleosome annotations. We reply to each of them in detail below. We want to stress that a computational approach has clear-cut strengths as compared to manual annotation in terms of throughput, objectiveness and reproducibility.

Chromatosome conformation: Despite our efforts to find alternative structures, exhaustive 3D classification yields the H1-containing chromatosome as dominant class. Single-particle cryo-EM and MD studies have shown that H1 binding constrains linker-DNA exit geometry and limits arm mobility (e.g., Zhou – PMID: 33238161 et al.; Woods et al. PMID: 32128577). Therefore, we use the class-average linker-arm directions as the most probable exit vectors for prediction, an assumption supported by the quality of our cryo-EM map obtained by subtomogram averaging (Fig. 1e–g).

To optimize our algorithm for alternative linker orientations in non-H1 containing nucleosomes, we would first need a data set that contains a significant number of such nucleosomes. We work on obtaining such data but do not yet have those at hand.

We want to add that the linker orientation, after assignment, in average deviates about 15 degrees from the ideal chromatosome starting position (see Letter Figure 2 below), which means that other orientations are detected.

Angular (and positional) Accuracy: This point has also been raised by other reviewers. See reply to comment 1.1 of Reviewer 1, and new Supporting Figure 11. In short, we explicitly assessed the robustness of our predictions to translational and rotational uncertainties in nucleosome identification by adding controlled noise to nucleosome coordinates and Euler angles in a crowded and dense in vitro structure (EMD-2601, see new Supplementary Figure 11e,f). The angular and positional accuracies of nucleosome orientations come from multi-particle refinement, with estimated uncertainties of ~1.6° and ~1.8 Å, respectively. As shown in Supplementary Figure 11e, linker predictions remain stable, with high F1 scores and precision up to perturbations far exceeding these reported uncertainties.

Exhaustiveness of nucleosome annotation (false negatives): False negatives are a known challenge in cryo-ET because experimental tomograms lack ground truth. This point has also been raised by Reviewer 2, point 1.2.

To address this point, we validated the algorithm on in-vitro data with known ground truth (compact 12-mer, EMD-2601): despite the high crowding, our method achieves high precision and robust F1 (Supplementary Figs 12). A leave-one-out test shows resilience to missed nucleosomes (F1~0.75) even when 4/12 are absent. By focusing on high-confidence pairwise links, predictions are intentionally sparse with correspondingly low false-positive rates.

Long-range connections: Our algorithm does not enforce long-range connections. Chains naturally terminate when no plausible partners remain. The prediction algorithm only assigns linkers probabilistically and in a stepwise manner. Since the data are sparse, many of our connections are simply di-nucleosomes. Still, the reconstructed fragments of multiple nucleosomes align with expected NRL distributions and show structural features such as nucleosome stacking and zig-zag motifs that match previous in vitro and in situ data.

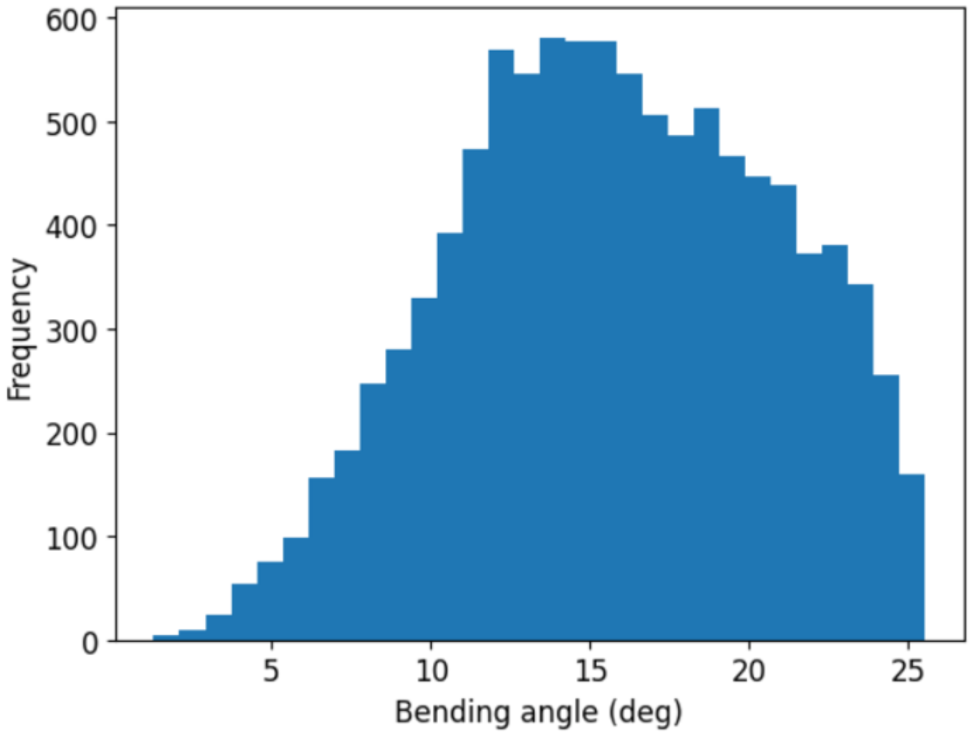

Letter Figure 2: Predicted linker bending angle. Histogram of the bending angle for all the predicted DNA linkers. The bending angle is calculated from the Eq. (2), as shown in Supplementary Figure 8.

Specific comments:

1. The use of the word 'tracing' for DNA linkers in the abstract and later in the manuscript is misleading. The authors present a 'prediction' of the linker or an 'estimate' of the linker trajectory (as they say in the discussion).

Reply: We understand the reviewer's point. Although tracing is defined as 'to follow an already defined path', which we think is what we do, our algorithm does not take into account the actually observed electron optical density between nucleosomes, e.g. by segmentation.

We want to stress that we very often have used the term 'predicted linkers' in the manuscript that makes the predictive character of our algorithm perfectly transparent. We nevertheless carefully reconsidered and adjusted our phrasing

throughout. We could not entirely eliminate the word trace, because it is also used in the methods part for patches of chromatin (not the linkers), where we think it is appropriate because it indeed refers to connections between confidently identified elements (the nucleosomes). Here, we added this statement:

> “We use the verb ‘trace’ here to describe the connection of nucleosomes into patches of chromatin by interpolating the DNA linkers in-between them.”

2. It is unclear how heterochromatin is defined. Do the authors mean only the 50 nm thick boundary layer adjacent to the nuclear envelope, or the entire volume of tomograms, or are they based on the density of nucleosomes? In the latter case, what does euchromatin look like? Can it be separated? No clear estimate of the extent of heterochromatin from the nuclear envelope is given. In my opinion, the attribution of the observed peripheral regions to heterochromatin seems hypothetical without precise mapping. This could be done by CLEM localization of heterochromatin-associated proteins such as HP1 and PRC, for example. The claim that the observed regions represent exclusively heterochromatin requires further evidence.

Reply: This point has also been raised by the other reviewers. We have included additional light microscopy data showing that the region captured in the field of view of our tomograms of up to 400 nm beyond the inner nuclear membrane is highly enriched for heterochromatin markers (see below).

For technical reasons, we cannot yet collect tomograms from the inside of the nucleus (because the density of the nuclear membranes is highly beneficial in improving tracking across tilts during acquisition and also in aligning the projections prior to reconstruction). We work on technical solutions for this problem, but at this stage cannot answer the question how euchromatin looks like.

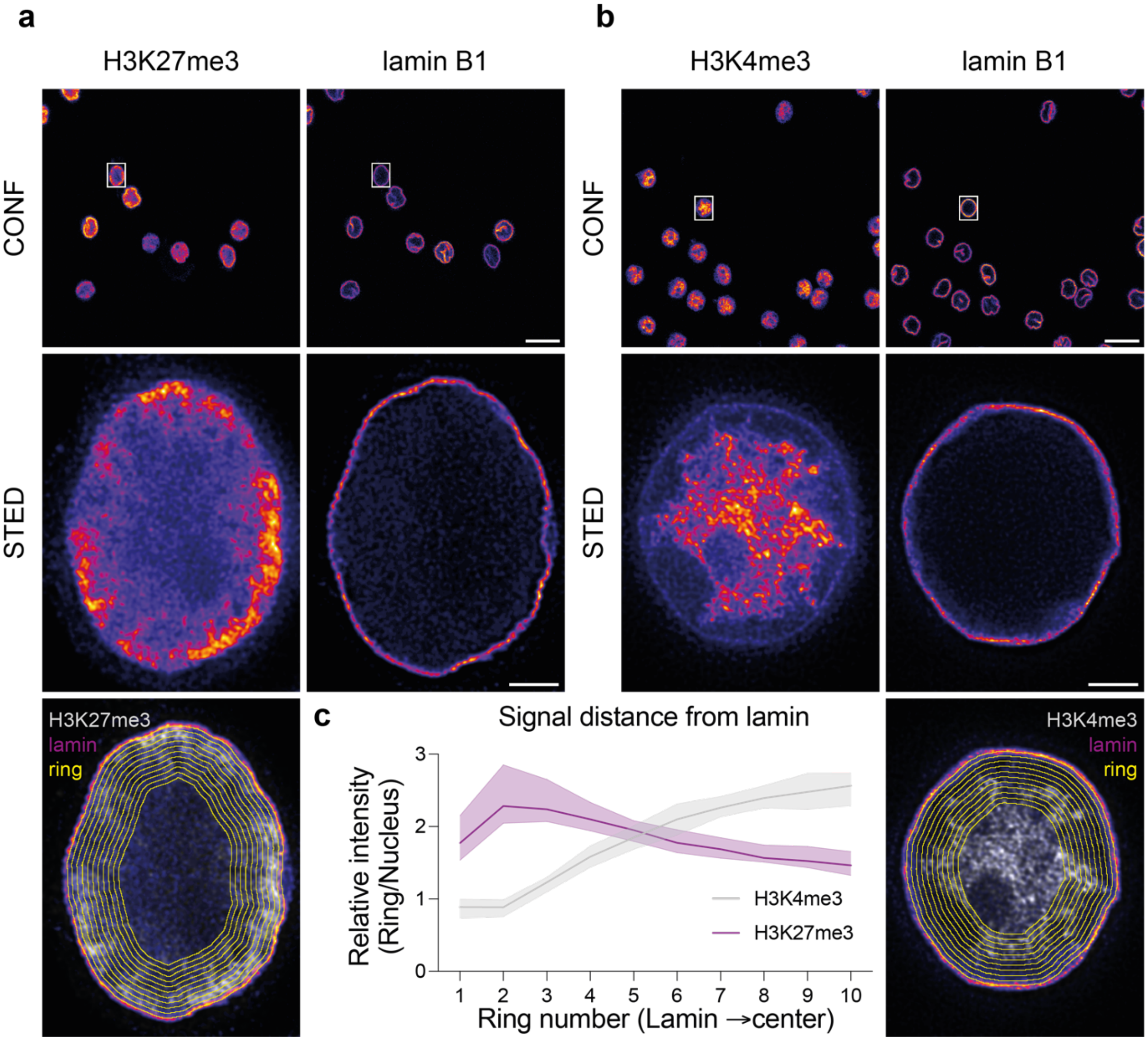

Supplementary Figure 1. STED imaging of chromatin markers in resting CD4+ T cells and quantitative analysis. Fire LUTs of lamin B1 and H3K27me3 (**a**) or H3K4me3 (**b**) signals. The top panels show a broad confocal view (CONF, scale bar = 10 µm), the middle panels a deconvoluted STED acquisition of a central Z-plane enlargement (STED, scale bar = 1 µm). The bottom panels display the channel overlap, with fire LUT for lamin B1, gray LUT for the histone mark, and ten concentric rings of 100 nm width generated from the lamin signal (yellow). (**c**) Quantification of signal intensity (mean gray values) measured in the indicated rings and normalized to the mean signal of the entire nucleus. The distance between rings is 100 nm. Graphs represent the median (solid line) and interquartile range (shaded area) of pooled cells from two independent blood donors (violet: H3K27me3, n = 8 and 7 cells from each donor; gray: H3K4me3, n = 12 and 10 cells).

3. The authors used a template-matching approach to identify nucleosomes. Detailed explanations are required to assess its effectiveness and the absence of a putative bias towards a single nucleosome conformation. The information provided by the authors is indeed insufficient: ‘a limited amount of debris was found’. How was the classification carried out?
?

The classes shown in Supplementary Figure 4 appear to be the result of post-alignment classification, which might be biased towards the original averaging.
?
Was any initial reference-free classification, for example multiclass classification-alignment procedure in Relion, performed after every two rounds of template matching to assess the conformational variability of nucleosomes?

Reply: We have very carefully looked but could not find the phrase 'a limited amount of debris was found' in our manuscript. Nevertheless, the question regarding the presences of a single nucleosome conformation is a very valid point and has also been raised by the other reviewers.

It is true that in the original version of our manuscript, we had only used an H1-containing reference for template matching. To exclude bias, we included additional analysis into Supplementary Figure 5 to address this point. This includes template matching with a H1-free reference and STA refinement against such a reference.

Even extensive classification using an H1-free reference did not result in decently resolved subtomogram averages, as shown in the new Supplementary Figure 5 (see reviewer #2 point 3). We furthermore did 3D classification with a featureless cylinder as initial reference with all identified particles but could only obtain a full chromatosome and a partial chromatosome (less H1-like density and shorter linker arms) structure. We interpret this observation such that the vast majority of nucleosomes contained in our tomograms do contain H1.

We have updated the methods section accordingly.

An arbitrary threshold was set by the authors for template matching. How exhaustive was the resulted annotation of nucleosomes? Did you test automatic against manual expert annotation on a representative volume? It is known that template matching can also bias the orientation of nucleosomes towards side views that have higher contrast. The angular distribution of selected nucleosomes should be presented.

Reply: We respectfully disagree with the assessment that the threshold is arbitrary. We use the fact that our tomogram volumes have the nuclear envelope as a divider in the middle and therefore include also cytoplasm. Thus, template matching analysis in the cytosol can be used as a negative control. We used a peak extraction threshold which excludes 99% of all peaks in the cytoplasm for each tomogram. This value was then applied to only extract peaks from the nucleus which are at or above this threshold, making sure that our particles have

cross-correlation values significantly above the background noise (as represented by false positives in the cytoplasm).

And, no, we have not tested manual annotation. As pointed out above, we are strongly in favor of automatic annotation because of throughput, objectiveness and reproducibility.

We have attached a figure (Letter Figure 3) to show the orientation distribution of our nucleosomes after STA refinement. As in previous reports, the side views are indeed represented to a higher degree than other views (Kelley et al https://doi.org/10.1101/2024.12.28.630444). However, since our linker annotations are sparse anyways, and our algorithm discards candidate linkages in which no confident assignment was possible, and chromatin orients freely in the nucleus, this does not have any influence on the conclusions of our paper. Missing wedge weighting normalizes this effect out during subtomogram averaging.

We refer to this in the limitations of our study section as follows: "The angular orientation of the detected nucleosomes was slightly biased towards top views, in which the DNA linkers are perpendicular to the electron optical axis. Since our greedy algorithm discards non-confident assignments, and chromatin orients freely in the nucleus, this should not influence the estimated linker length pattern."

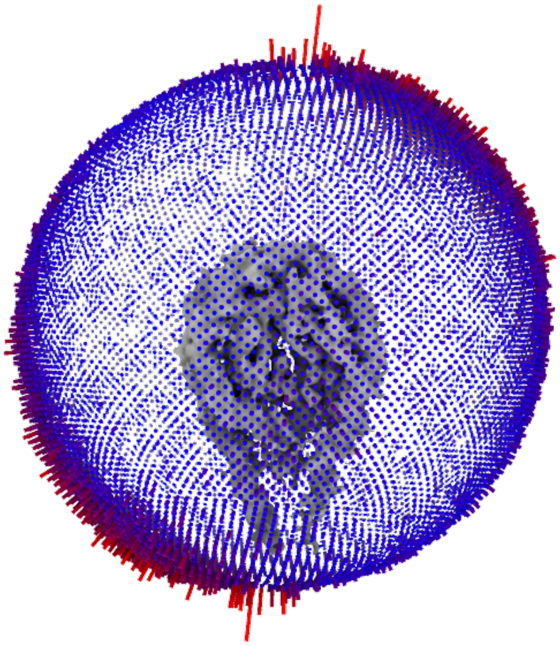

Letter Figure 3: Orientational distribution of unsymmetrized (C1) chromatosome STA. The red peaks indicate more often represented side views of the nucleosomes which have higher contrast.

4. The term “Multi-nucleosome arrangement” introduced by authors is difficult to define. “Multy-” would fit to anything starting from nano-domains to chromosome territories.

Reply: We use the term oligo-nucleosome in the revised version of our manuscript.

5. Chromatin is very dynamic even in very compact states in vivo. The lowest energy predictions would correspond to the equilibrium state that is very unlikely for the dynamic native chromatin. It might result in bias towards straight linkers.

Reply: We agree that native chromatin is highly dynamic and does not necessarily adopt lowest-energy conformations. However, our approach does not minimize the global energy of the system. Once nucleosome positions and orientations are fixed, our approach computes the relative probabilities of all plausible linker paths based on bending energetics.

Importantly, this probabilistic scoring allows and account for DNA bending as shown Letter Figure 2. Equation (3) penalizes both highly bent and overly stretched linkers, but those are still possible (see Letter Figure 2). Physically, large bending angles for a short piece of DNA would require an enormous amount of energy. In cells, this could happen with other machinery, but we cannot detect it.

We further validated the approach using in vitro chromatin reconstructions with known geometries, where the algorithm accurately recovered correct linker configurations across different levels of compaction and linker curvature (see Supplementary figure 11d).

To my understanding, the connectivity model and physics-based prediction of the linker path assume absolute precision in the positions and orientations of the picked nucleosomes, which may not be entirely realistic. How robust is the model to potential translational (1–4 pixels) and orientation noise (e.g., 10–30°)?

Reply: The reviewer raises an interesting point, namely how robust is our algorithm to uncertainties in the position and orientations of the nucleosomes. This point has also been raised by other reviewers. See reply to comment 1 of Reviewer 2, and the new Supplementary Figure 11.

We explicitly assessed the robustness of our predictions by adding controlled noise to nucleosome coordinates and Euler angles in a challenging, dense and well-characterized in vitro structure (See new Supplementary Figure 11). The angular and positional accuracies of nucleosome orientations come from Relion 3D refinement, with estimated uncertainties of ~1.6° and ~1.8 Å, respectively. As shown in Supplementary Figure 11e,f, linker predictions remain stable, with high

F1 scores and precision up to perturbations far exceeding these reported uncertainties.

The MD simulation movie shows very limited nucleosome movements without H1 association/dissociation. How realistic are the binding stability and time of H1?

Reply: The reviewer is correct, the globular domain of the H1 proteins show very limited movement in the simulations. This is expected due to the time-scale of the simulations (ns) and the residence times of H1. It has been reported in the literature that H1 unbinding occurs in the order of minutes (https://doi.org/10.1101/2025.03.05.641622) and requires the competition with other charged proteins not present in our simulations (https://doi.org/10.1038/s41557-021-00839-3).

6. Line 55. "stiff linkers" statement is wrong. Stiffness is a relative concept: compare the stiffness of DNA and actin filaments. The Luger et al. citation does not apply to DNA linkers.

Reply: We have rewritten the sentence. It now reads:

> p3: 147 base pairs (bp) of DNA are wrapped in a left-handed superhelical turn around this octamer, and flanked by DNA linkers of variable length that project towards the next nucleosome[2].

7. Line 63. H1 histones have general affinity to DNA and can bind everywhere to chromatin indeed in vivo, not only in the relation to the DNA entry/exit regions.

Reply: The sentence in the introduction now reads:

> p3: "H1 variants can bind to the two DNA linkers, the DNA wound around the histone octamer, or all three DNA structures simultaneously, resulting in differential stabilization and either on-dyad or off-dyad arrangements"

8. Lines 87-90. The zigzag topology of chromatin in situ was directly identified previously (https://doi.org/10.1101/2022.08.16.502515).

Reply: The reviewer is correct; we have added the reference.

> pp. 4-5: "Studies in Drosophila embryos[17] and frog erythrocyte nuclei[22] have pointed to irregular zig-zag chromatin folding patterns."

9. Line 124 and the further statements about C2 symmetry. The chromatosome

is not a completely symmetrical structure. Can you explain how symmetry was applied to it?

Reply: We are aware that the chromatosome is not completely C2-symmetrical due to H1 being bound. The core nucleosome structure is, however, C2-symmetrical and C2 symmetrization in the Refine3D routine in Relion3 was used to enhance the structural features of the histone core octamer. We include both the C2-symmetrized (with better core octamer resolution, Figure 1e,f,g) and the C1-unsymmetrized chromatosome (with better resolved H1 density, revised Supplementary Fig 4). The linker prediction was performed on the particle list originating from refinement with C1 symmetry (meaning no symmetrization).

10. Line 392. The term ‘vitrification’ should not be used if cell vitrification is not justified. I cannot judge as access to the raw tilt series was not provided.

Resting T cells are a particularly small mammalian cell type and generally vitrify very well, which is one reason why we chose them for this study. We always screen our tomograms for non-vitrified patches and are confident that the term is justified. Of course, the original data will be deposited prior to publication of the study.

11. Supplementary Figure 6b. It is visually difficult to relate the density seen in the images to DNA. Where are the nucleosomes located? I assume they must be resolved as well. Why are only the YZ and XZ species shown? Is the scale bar 2 nm? Is the citation 49 correct?

Reply: We thank the reviewer for those comments. Now we make the location of the nucleosomes in the density clear (see revised Supplementary Fig 9b and d; previously S6). The nucleosomes are indeed resolved as blurry densities on either end of the linker DNA (indicated as N1 and N2 in revised Supplementary Fig 9b,d). Since these averages were aligned on the linker DNA it is to be expected that the nucleosomes are not well resolved. Note that a difference in 1 bp will induce a rotation of ~33 degrees on the second nucleosome. Only the YZ and XZ views are shown since the XY view shows a less informative circular density “looking down” on the nucleosome+linker density. We now include the scale bar information; The bar indicates 5 nm. We apologize for the citation error in the supplementary information where reference 49 was incorrectly not referring to Relion3, this has been corrected.

REVIEWER COMMENTS

Reviewer #1 (Remarks to the Author):
The authors have adequately addressed my previous comments in the revised manuscript. I am satisfied with the revisions, and I recommend the manuscript for publication.

We thank the reviewer for the positive assessment of our manuscript.

Reviewer #2 (Remarks to the Author):
I appreciate the authors' efforts in addressing the previous comments and further validating their method using in vitro chromatin map. The study presents a potentially powerful approach for analyzing chromatin organization. However, several conceptual and methodological issues remain to be clarified, and some results require further interpretation before the manuscript can be considered for publication. My main comments are as follows:

1. The reported overall resolution does not represent the accuracy of each individual particle. The reported high resolutions do not guarantee that each particle in the dataset is aligned with high accuracy. In practice, many particles—especially those with low SNR or suffering from ion-beam–induced damage—may be poorly aligned or even appear false positives. Although the overall resolution may remain high due to the majority of good particles, per-particle alignment uncertainty can significantly affect downstream analyses, particularly the linker DNA prediction, which depends on precise nucleosome positioning.
I strongly recommend that the authors establish a method to quantify the alignment precision for each individual particle, rather than relying solely on the global resolution estimate. This would also help explain the observed reduction in recall for linker prediction when alignment inaccuracies occur. If this is technically infeasible, the authors should at least discuss this potential source of error explicitly and avoid assuming that a high-resolution average map implies uniformly accurate per-particle alignment.

As mentioned by the reviewer, we acknowledge that a subset of particles may exhibit a lower SNR. However, to the best of our knowledge, a metric for quantifying the precision of individual particle orientations has not yet been established for subtomogram averaging (STA). Such a metric is a fundamental challenge in cryo-ET that so far remains unaddressed, at least for in situ data. In our view, developing and benchmarking such a metric is beyond the scope of this study.

That said, we want to point out that we have validated our linker predictions in several ways:

1. In Supplementary Fig. 10d we show that an increase in the predicted linker length correlates well with the increasing linker DNA densities, thereby arguing that potentially poorly aligned or false positive particles do not substantially interfere with the downstream analysis of linker length prediction.
2. In the first round of revisions, we benchmarked our algorithm on in vitro data with known ground truth to establish how the outcome is influenced by inaccuracies and missing assignments.

3. We show that the predicted linker lengths on average agree with genomic measurements by protected read sequencing.

We agree that we cannot exclude that a subset of misaligned particles is present in our data. However, the available evidence indicates that this effect is unlikely to dominate our conclusions.

We now refer to this possibility in the limitations of the study section as follows:

> *p.16: "In addition, we estimated the accuracy of the orientations and positions of the nucleosome particles only with global metrics for the entire ensemble, but not for each individual particle. Therefore, due to local fluctuations in SNR the accuracy of the linker DNA prediction may locally differ. Nevertheless, given that our linker prediction correlates well with the experimental data (Supplementary Fig. 10d), particles with poor alignments likely make up only a minor fraction of all particles."*

2. It should be clarified whether the nucleosomes used for linker prediction were all obtained from the STA process or whether template-matching (TM) particles were also included. Figure 3 shows both STA and TM. If TM-derived nucleosomes were used, and if their orientations can be accurately determined, then it should be discussed why STA is necessary at all. Alternatively, if the method is robust to moderate angular noise, the authors might justify using TM-only data for prediction, demonstrating the claimed robustness of the approach.

The nucleosome particles used for linker prediction were initially localized with TM. Their orientations and positions were not used directly, but rather refined with STA to obtain more precise positions and orientations with reasonable computational expense.

We have clarified this in the manuscript as follows:

> *p. 22: "We used the 33,560 positions obtained from subtomogram averaging and M refinement for further spatial analysis and linker prediction."*
>
> *p.24: "Starting from the nucleosome positions and orientations obtained from STA after M refinement"*
>
> Fig. 3 (p. 10) now reads: "*Nucleosome positions and orientations obtained from STA".*

3. Regarding the H1-free category, in Supplementary Figure 5, the right-hand averaged nucleosome clearly shows weak or missing H1 density, suggesting the loss or flexibility of H1. Moreover, the DNA trajectory in these cases does not appear to follow an H1-constrained path. Even in heterochromatin regions, the presence of H1 cannot be assumed to be homogeneous. The authors should discuss this heterogeneity explicitly and reconsider their interpretation of the "H1-free" population.

The less well-resolved “partial chromatosome” class shown on the right in Supplementary Fig. 6 likely represents a mixture of both H1-bound and H1-free nucleosomes. Despite considerable effort, we were not able to obtain a class average of an H1-free nucleosome through 3D classification. We maintain that the H1-free population is only a small subpopulation of all particles that we detected. Please also see response to reviewer #3 point 1 (ii). We already discussed the potential negative effect that this may have on our linker algorithm in the limitations of the study (p. 16). We have now further revised this section to be fully transparent:

> *p.16: “In this study, the majority of the detected nucleosomes appears to be H1-bound. We based this interpretation on the observation that extensive classification efforts did not result in highly resolved subtomogram averages of H1-free nucleosomes (Supplementary Fig. 6). However, a minor subpopulation of H1-free nucleosomes likely exists in the nuclear regions we analyzed, leading to a certain degree of nucleosome heterogeneity. At this stage, we cannot fully exclude the possibility that the performance of our algorithm is negatively influenced by the presence of such H1-free nucleosomes with more flexible linker arms, even if they are not prominent in our samples.”*

4. Although low-magnification tomograms do not show individual nucleosomes clearly, they are essential for assessing the overall chromatin compaction state. The authors should provide representative low-magnification maps for multiple lamellae to demonstrate that the observed chromatin looseness is a general feature rather than a selection bias from the 14 tomograms.

To address this point, we have added a new Supplementary Fig. 1 showing the respective low-magnification lamella maps. At this magnification, the entire nucleus has a similar appearance, and therefore they do not show any apparent visual differences in terms of chromatin compaction state. We emphasize here that the 14 tomograms analyzed in this study have not been divided into regions that appear unusual or biased in any way.

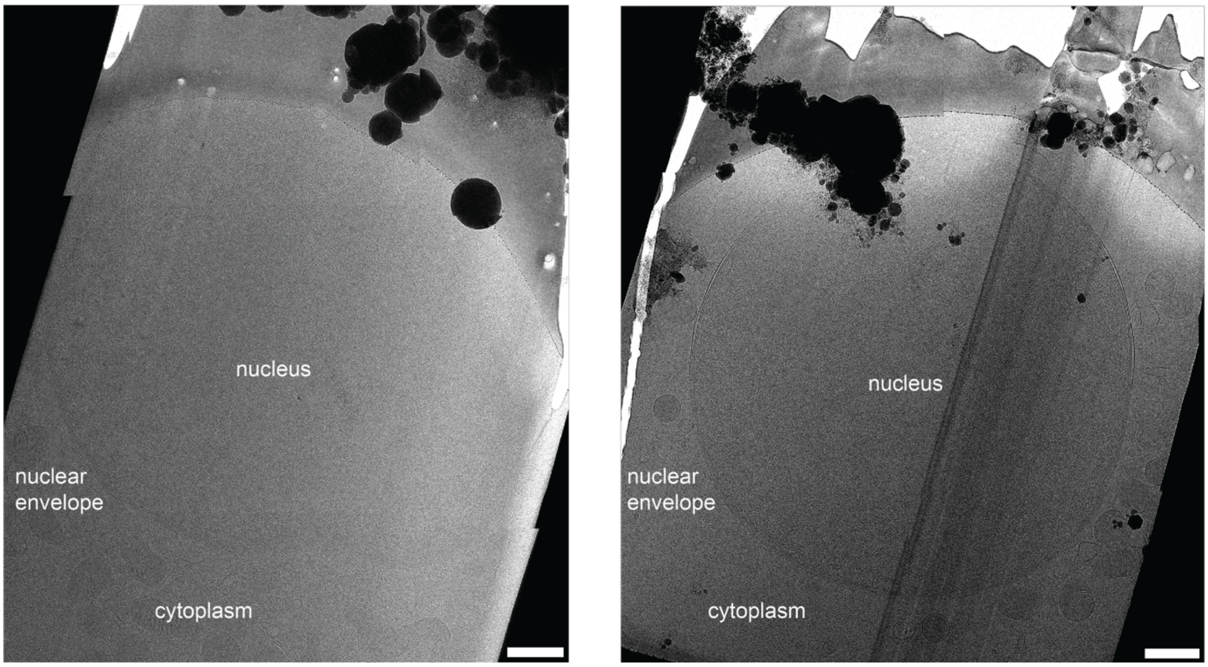

New Supplementary Fig. 1. Lamella maps. Low-magnification lamella maps for two different lamellae with nucleus, nuclear envelope, and cytoplasm indicated. There are no apparent visual differences in terms of chromatin density inside the nuclei. All tilt series were collected at the nuclear envelope. Scale bar indicates 1 µm.

5. It remains unclear why certain nucleosomes are incorrectly connected, even under in vitro conditions where the nucleosome orientation appear correct. The authors are encouraged to identify potential patterns or contributing factors that lead to incorrect connections. Including such a discussion would strengthen the methodological insights of the manuscript.

In the analysis of the in vitro chromatin map (Supplementary Fig. 12,13), we did observe a small number of false positive connections. These arose mainly in crowded local configurations during the 'drop out' analysis, where the removal of one of multiple stacked nucleosomes resulted in skipping of the false negative nucleosome because the two nearby nucleosomes to appear as a plausible direct pair. In such cases, the algorithm selects the most favorable local connection from the available geometry, which can occasionally favor an incorrect shortcut (Supplementary Fig. 13).

We note, however, that this in vitro chromatin fiber is a particularly challenging test system because of its compactness and symmetry. In cellular tomograms, we expect such cases to be less frequent, as the relative orientations and distances of nucleosomes more often yield lower probabilities for these incorrect connections (see Supplementary Fig. 10a).

6. The statement that the predicted linkers "require significant bending" seems conceptually inaccurate. The in vitro chromatin map is all straight DNA. The authors should revisit this interpretation and clarify whether these cases represent algorithmic artifacts or structural deviations.

We respectfully disagree with the statement that 'The in vitro chromatin map is all straight DNA.' The in vitro chromatin map actually is not all straight DNA, but rather shows bending. In Supplementary Figure 12d the bending of the linker DNA can be seen when comparing the colored predicted (straight) linker DNA to the experimentally observed density. In particular in case of the linkers marked in red, the experimentally observed connection clearly bends above the predicted linker. We have added a note to the figure legend to make this clearer to the reader:

> Supplementary Fig. 12: "*Note that in cases where the predicted linkers are depicted in red, the experimentally observed density shows a significant degree of bending.*"

7. The claim that the nucleosomes are "off-dyad" is not well supported. The current resolution and alignment precision are insufficient to make such an assignment. The apparent H1 density appears more consistent with an "on-dyad" position. The authors could consider to fit the density to a PDB model and provide quantitative evidence supporting this claim.

We agree with the reviewer's concern that we cannot make a definitive claim regarding on-dyad or off-dyad positioning given the current resolution and alignment precision. We have thus revised the manuscript to remove this claim. The figure legend of Supplementary Fig. 7 now reads as follows:

> Supplementary Fig. 7: *"3D classification in Relion 3.1 [34] identified nucleosome classes in which H1 was shifted towards either DNA linker (highlighted with orange arrows)."*

And the main text:

> *pp. 5-6 "Instead, we identified nucleosomes in which H1 was shifted to either DNA linker (Supplementary Fig. 7)."*

8. It would greatly enhance the manuscript's impact to include a specific example demonstrating what can be done with the reconstructed DNA. This would illustrate the practical utility and broader relevance of the method.

We assume that the term 'reconstructed DNA' refers to connected nucleosomes in our tomograms.

For one, our approach of measuring linker lengths could be used to determine local nucleosome spacing in different types of datasets. One could, for example, envision this being used to compare chromatin in different states of differentiation for one cell type or between different genetic or pharmacological perturbations. With further improvements, acquisition of active chromatin will also become feasible, thus allowing a comparison of different chromatin states within one cell, for example in response to external stimuli.

We have added the following text to the discussion:

p. 15: "*For example, one could envision our approach being used to compare different cellular states associated with, e.g., cell differentiation, genetic and pharmacological perturbations, or in response to external stimuli.*"

Reviewer #3 (Remarks to the Author):

The authors have substantially improved the manuscript, addressing most of the previous comments with careful revisions and additional analyses. The quality and clarity of the work have significantly increased. However, two important methodological issues and one terminology-related concern remain that require further clarification or adjustment.

1. Preferential orientation pattern of the angular distribution of picked nucleosomes

The authors have presented the angular distribution of the picked nucleosomes, which reveals a clear bias toward certain orientations. I disagree with the authors' formulation that "the angular orientation of the nucleosomes was slightly biased towards top views." This statement does not accurately reflect the situation shown in Letter Figure 3, which reveals a pronounced preferential orientation pattern, visible as distinct angular segments, very similar to those reported by Kelley et al. (https://doi.org/10.1101/2024.12.28.630444) indeed. The distribution indicates that a relatively narrow range of orientations contains a disproportionately high number of picked nucleosomes.
The angular distribution figure should be added to the Supplementary Information in two views (similar to Kelley et al., Supplementary Figure 8G), to give readers the opportunity to recognize this important limitation.

We thank the reviewer for pointing this out. We have now performed the suggested orientation bias analysis, which indeed yielded similar results to those reported by Kelley et al. although it is less pronounced in our data (Jensen-Shannon distance to uniform distribution (values between 0-1) of ~0.31 compared to ~0.45 for Kelley et al.). For reference, a ribosome particle list with 30k particles (obtained from Hoffmann et al. https://doi.org/10.1038/s41467-022-34997-w), which should be close to uniform in angular distribution, exhibits a Jensen-Shannon distance of ~0.07. This analysis is now included in Supplementary Fig. 3c.

In light of these results, we also revised the respective section of the manuscript as follows:

p.15: *The angular distribution of the detected nucleosomes was biased towards side views (Supplementary Fig. 3c), where the thin edge of the nucleosome disk showing the two DNA gyres is parallel to the electron optical axis. These findings are consistent with a recent study on Chlamydomonas reinhardtii, which also reported nucleosome structures in situ [24]. This will affect the number of false negative nucleosomes, thus resulting in an underestimation of nucleosomes present in the sample. However, since our algorithm estimates probabilities in pairs, discards non-confident assignments, and chromatin orients freely in the nucleus, we do not expect a strong influence on the estimated linker length pattern.*

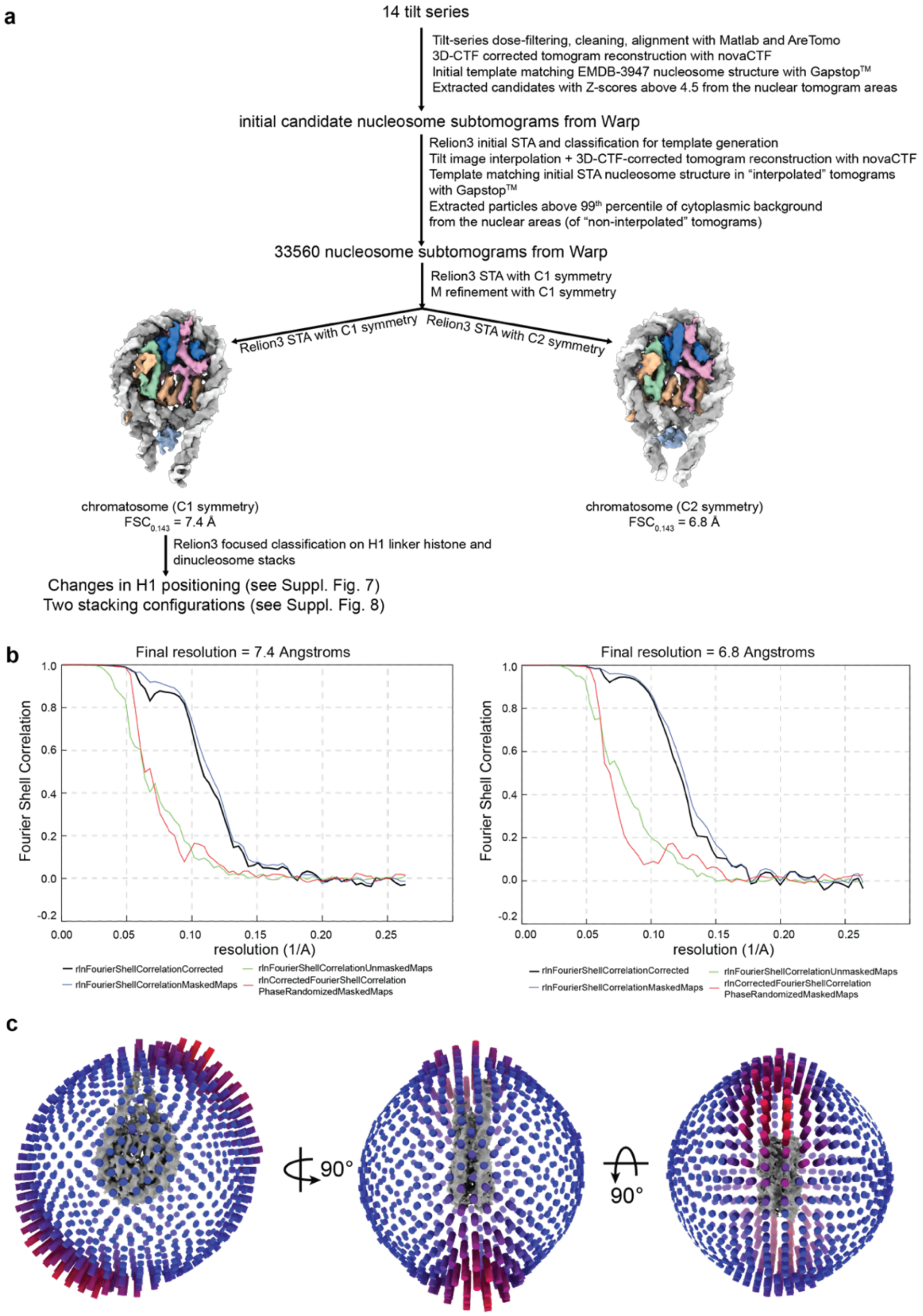

Supplementary Fig. 3. Image processing workflow for the chromatosome. a Workflow describing the steps taken from raw tilt series to final chromatosome STA structures. **b** Fourier shell correlation curves from Relion 3.1[34] after M refinement[59] for structures with C1 or C2 symmetry imposed. **c** The angular distribution of the detected nucleosomes exhibits a bias towards side views, where the thin edge of the nucleosome disk showing the two DNA gyres is parallel to the electron optical axis.

This observation also has important implications:

(i) The presence of angular sectors with severalfold higher particle counts demonstrates that nucleosome picking was far from exhaustive. Consequently, the

measured nucleosome density derived from this picking underestimates the actual nucleosome density. This bias may affect relative density comparisons at local scales around 30-50 nm, because chromatin fiber orientation might influence its picking efficiency. For example, a segment of compact chromatin fiber oriented close to the z-axis would likely contain more top views of nucleosomes, which have a lower probability of being picked and might be misinterpreted as chromatin-free fraction. To improve understanding, it would be useful to compare the spatial distribution of nucleosomes in preferred orientations with one of all other orientations.
Furthermore, the orientation bias will influence the global nucleosome density values reported. I suggest that the authors estimate and apply a correction factor by comparing the frequency of nucleosomes picked at preferred orientations with ones of the low picking frequency. Provided that the corrected nucleosome density values remain physically consistent with the spatial constraints of the analyzed chromatin domains, this adjustment would yield a more realistic estimate of nucleosome concentration. If it leads to unrealistically high densities, this would indicate the presence of false positives or a non-uniform orientation of chromatin fibers within the tomogram(s). The latter, although unlikely, would still be important to investigate.

We thank the reviewer for raising our attention to this issue. As detailed above, performing the orientation bias analysis indeed showed that a considerable number of nucleosomes were missed in our analysis.

As suggested, we examined whether nucleosomes in preferred orientations show a distinct spatial distribution compared to all other orientations. We did not observe any obvious spatial segregation; nucleosomes in preferred orientations appear broadly distributed throughout the tomograms (see Fig. R1).

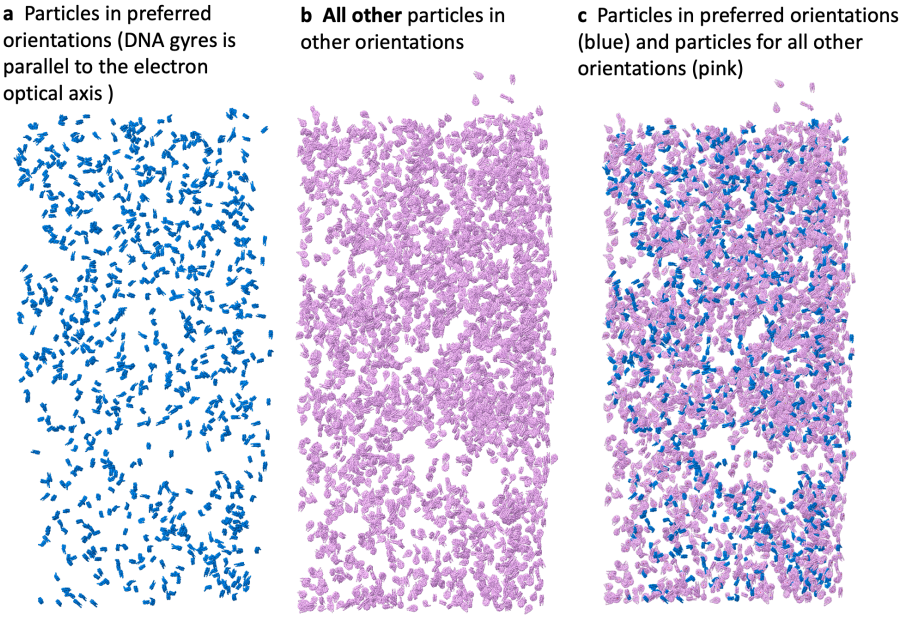

Fig. R1: Spatial distribution of nucleosomes with preferred and non-preferred orientations. **a** Nucleosomes in preferred orientations, defined here as particles with DNA gyres parallel to the electron optical axis. **b** Nucleosomes in all other orientations. **c** Overlay of both populations, showing no obvious spatial segregation of preferred-orientation particles within the tomogram.

In addition, as also suggested by the reviewer, we have estimated a correction factor to our detected nucleosomes to account for under-picked nucleosomes in certain

orientations using two different methods: (i) manual expert inspection, and (ii) adjustment of the angular histogram to the expected uniform angular distribution.

(i) We manually picked additional particles from three different tomogram slices in two different tomograms (see Fig. R2). This approach yielded a correction factor of (1.58±0.13)X. We note, however, that this method is labor intensive and subjective.

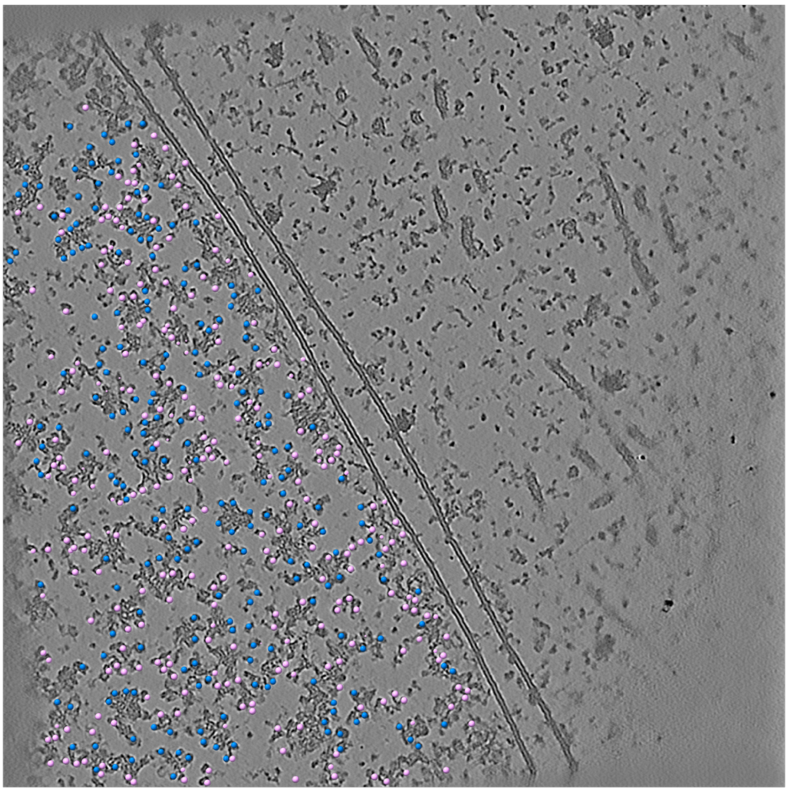

Fig. R2: Manual picking of additional particles. Slice through IsoNet2 (Liu et al. https://doi.org/10.64898/2025.12.09.693325)-denoised tomogram with particles detected with template matching in pink and manually picked additional particles in blue.

(ii) As an alternative, we created a 3D histogram of angular orientations and estimated the number of particles required to fill up the underrepresented bins. The details are in the methods section of the manuscript (see below). This approach yields a correction factor of about 2.14X.

Although both, the manual and computational metric yield comparable numbers, we opted for including the computational metric into the revised manuscript, because it is scalable and more conservative. We state that we estimate the current approach to detect around half of the nucleosomes present in the tomograms, i.e., a correction factor of 2X, as follows in the methods part:

> *p.21: "To assess the extent of the orientation bias, we adapted existing code from the pyem python package* [62] *to assign particle orientations to angular bins utilizing the HEALPix library. Each nucleosome orientation was assigned to one of 768 HEALPix bins (HEALPix order value = 3), then all bins with counts below the mean+1sigma bin count value were filled up to that value.*

This approach yielded a correction factor of about 2.14X needed to obtain a balanced angular distribution of nucleosomes.”

Since the under-picking does affect the nucleosome density estimations (local and global) that we reported previously in Figure 2. Therefore, we have decided to remove the density estimations from this figure (panels a and e) because we cannot infer local effects of under picking. Instead, we refer to the number of detected nucleosomes per volume. Additionally, we have rephrased the text in the other panels in Figure 2 to clearly state that these calculations were based on the number of detected nucleosomes which should be seen as a lower boundary.

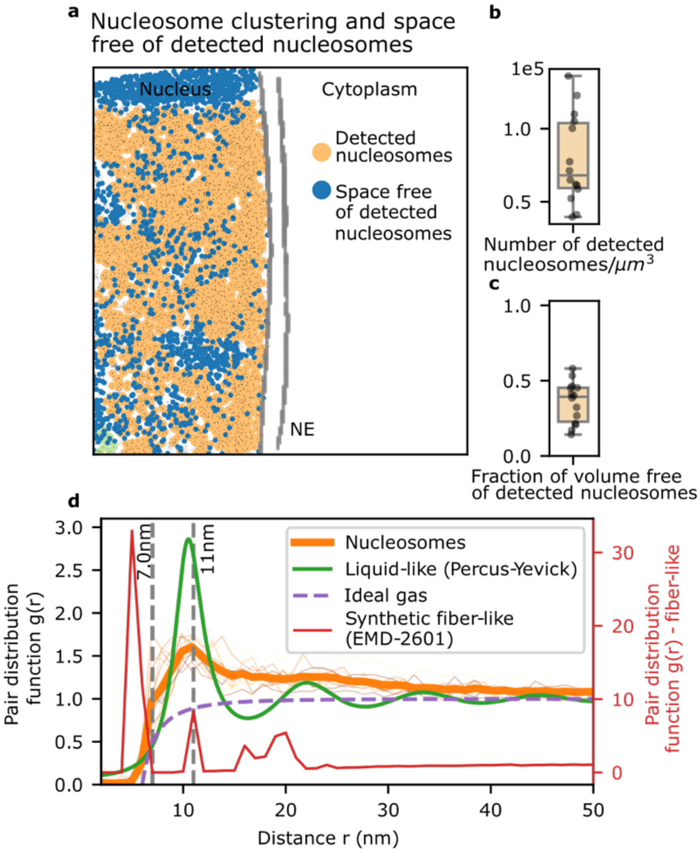

Fig. 2: Spatial properties of detected nucleosomes at nuclear periphery of T cells. a Hierarchical density-based clustering (orange and green clusters) and space free of detected nucleosomes visualized from the same exemplifying tomogram as in Fig. 1a. **b** Overall number of detected nucleosomes per unit volume with each tomogram displayed as single dot. **c** Fraction of nuclear volume free of detected nucleosomes with each tomogram displayed as single dot. **d** Pair distribution function g(r) of the detected nucleosomes. Reference curves for the ideal gas and liquid-like distributions[32], and a synthetic tomogram made of 30-nm-fiber segments (EMD-2601[7]) are included for comparison. Gray dashed lines are the peaks of nucleosomes g(r) function.

The main manuscript text has also been changed to the following.

p 7. “We next used the set of detected nucleosomes to determine spatial properties (Fig. 2). Clustering analysis revealed an overall densely packed nucleoplasm with some occasional nucleosome-free patches, e.g.,

underneath nuclear pores (Fig. 1a). We also quantified how much nuclear volume was occupied by the detected nucleosomes as compared to free space (Fig. 2a, c), and found that less than half of the volume at the nuclear periphery is free of detected nucleosomes, using a 5 nm probe volume. Finally, we quantified native chromatin in terms of the nucleosome pair distribution function $g(r)$ (Fig. 2d). In terms of $g(r)$, we find chromatin to be intermediate between a liquid and a gas with two short-range peaks at 7 and 11 nm center-to-center distance likely representing stacked (Supplementary Fig. 8) and edge-contacting nucleosomes, and a gradual decay beyond 20 nm indicating a weak tendency to condense (Fig. 2d). The relatively featureless curve provides no evidence for long-range order in chromatin, arguing against fibrous chromatin structures (theoretical curve for 30-nm fiber shown for comparison in Fig. 2d), even if one accounts for false negatives in nucleosome detection."

(ii) The presence of preferred orientations will likely influence particle classification. Because of the missing wedge, a sufficiently high population of nucleosomes whose superhelical axes are parallel or nearly parallel to the z-axis of the reconstruction is essential to discriminate between chromatosomes and nucleosomes with more distant entry and exit DNA segments, characteristic of the H1-free state. A dominance of side views among the picked nucleosomes could therefore hinder the detection of an H1-free class. Since the conformational uniformity of nucleosomes as chromatosomes is a key foundation of the linker prediction in this study, it would be important to explore this potential bias. For example, this could be done by balancing the angular distribution of nucleosomes, by reducing the number of particles picked in preferred orientations, and rerunning the classification from scratch, aiming not for a high resolution, but for exploring conformational diversity.

We reran the classification with a more balanced particle list as suggested by the reviewer but still did not obtain an H1-free nucleosome class.

In more detail, we again assigned particle orientations to angular bins and randomly removed particles in preferred orientations (i.e., in bins with higher counts than the median count) up to the point at which each angular bin contained the median count of particles or fewer. This process produced a particle list with 16829 out of the original 33560 nucleosomes. This new balanced particle list (Jensen-Shannon distance of ~0.17, as compared to the ~0.31 of the original list) was then used for 3D classification with a featureless cylinder reference in Relion 3.1. This did not yield an H1-free class and we therefore conclude that at least a large majority of our detected nucleosomes are H1-bound.

The results of nucleosome picking and classification in Chlamydomonas reinhardtii reported by Kelley et al. (https://doi.org/10.1101/2024.12.28.630444) support this concern. As shown in Supplementary Figure 8D of that work, the initial classification also revealed mainly chromatosome-like conformations. However, since the picking in that study covered the entire nucleus of Chlamydomonas reinhardtii, which mainly contains open euchromatin, it is unlikely that majority of nucleosomes were chromatosomes.

We agree with the reviewer that the points regarding the orientation bias during particle detection all have to be taken into account and we have revised the manuscript accordingly. However, as pointed out above, we are not convinced that we have exactly the same effect size in our data as was reported for the chromatosome-like conformations in Chlamydomonas. In contrast to the 3D maps shown in Fig. S5 of Kelley et al. Mol Cell 2026, we observe a more distinct density for H1 (see Fig. 1, Supplementary Fig. 5). We are thus confident that we observe true H1-bound nucleosomes in our dataset. This being said, we cannot fully exclude the presence of small sub-population of H1-free nucleosomes. We have revised the text to make this transparent to the reader. Please see our response to reviewer #2- point 3 on the possibility of H1-free nucleosomes.

2. Validation of prediction robustness

"Reply: The reviewer raises an interesting point, namely how robust is our algorithm to uncertainties in the positions and orientations of the nucleosomes. This point has also been raised by other reviewers. See reply to comment 1 of Reviewer 2, and the new Supplementary Figure 11.
We explicitly assessed the robustness of our predictions by adding controlled noise to nucleosome coordinates and Euler angles in a challenging, dense, and well-characterized in vitro structure (see new Supplementary Figure 11). The angular and positional accuracies of nucleosome orientations come from Relion 3D refinement, with estimated uncertainties of ~1.6° and ~1.8 Å, respectively. As shown in Supplementary Figure 11e,f, linker predictions remain stable, with high F1 scores and precision up to perturbations far exceeding these reported uncertainties."

The estimated uncertainties provided by Relion represent general statistical values and are not necessarily precise for each particle in noisy data. Therefore, a detailed assessment of the robustness of the linker prediction to variable rotational and translational noise levels would be essential for the study.
To my understanding, all robustness checks were performed only on the 12-mer array EMDB-2601. This is a highly compact structure consisting of three nucleosome tetramers with constant linker length and a repetitive geometry of linkers. It has very limited geometrical freedom and thus does not represent the geometrical diversity of chromatin in situ. I therefore disagree that this structure constitutes a representative in vitro model for testing prediction robustness.

Tomographic datasets presented in the work of Jentink et al. (https://doi.org/10.1016/j.molcel.2023.08.017)) may provide a more relevant and realistic in vitro testing system. These annotated datasets of native isolated human chromatin fragments are publicly available (https://datadryad.org/dataset/doi:10.5061/dryad.ttdz08m21).

We appreciate the reviewer's suggestion to test the robustness of our algorithm on other datasets besides the 12-mer array.

As suggested, we explored the dataset from Jentink et al. However, a systematic robustness analysis on that dataset would require de novo annotation and refinement of nucleosome positions and orientations, as the available annotations are not

accurate enough for our linker-prediction workflow. In practice, this would require substantial manual re-assignment and validation of particle poses, which is non-trivial and beyond the scope of the present study.

On top of this, there is another technical obstacle: We performed template matching of nucleosomes using the pytom-match-pick software package (Chaillet et al. https://doi.org/10.1016/j.yjsbx.2025.100125). We were not able to run our regular workflow with GAPSTOP™ (Turoňová. https://doi.org/10.5281/zenodo.10822455) on this dataset because we could not obtain the necessary metadata to create the precise wedge masks that GAPSTOP™ requires. Therefore, analysis shown in Fig. R3 below is not comparable to the technical standards employed throughout our paper.

Nevertheless, for a manually annotated example from that dataset (Fig. R3), our algorithm predicted linker connections that agree well with the experimentally observed densities. We therefore consider this dataset supportive of the general applicability of the approach, while a full reprocessing and benchmarking effort would require a dedicated study.

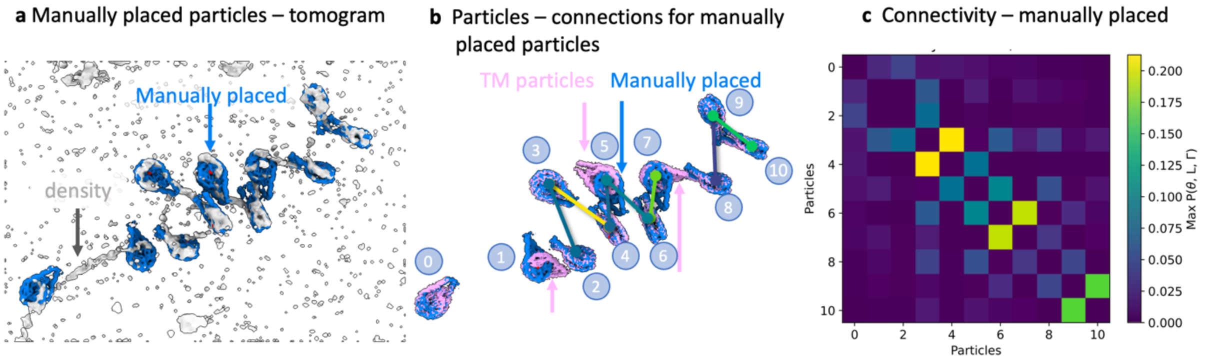

Fig. R3. Testing of the prediction algorithm on in vitro dataset. a Tomographic isosurfaces with manually placed nucleosomes (blue) overlaid on the density map. **b** Same region showing the manually placed nucleosomes (blue), the corresponding template-matching particles (pink), and the linker connections predicted from the manually placed particles. Nucleosomes are numbered for reference. **c** Connectivity matrix derived from the manually placed particle set. The template matching was performed with 5 degree global angular sampling on the deposited bin1 tomographic volumes.

Specific comments:
1. Supplementary Figure 11: It is not clear how rotational noise with σ = 10°–30° was applied to the tightly packed EMDB-2601 structure without causing steric clashes between neighboring nucleosomes. It should by also clarified whether this σ value applies to each rotational angle.

The rotational noise was introduced by adding independent Gaussian perturbations to each of the three Euler angles (ϕ, ψ, and θ); thus, the stated σ=10-30 deg applies to each angle separately. We did not explicitly filter perturbed configurations for steric clashes. As in the experimental STA workflow, the goal here was not to generate physically optimized chromatin conformations, but to assess how robust linker prediction remains under increasing uncertainty in nucleosome positions and orientations.

2. A statistical comparison should be provided for the charts shown in Supplementary Figures 11 and 12.

We thank the reviewer for asking us to strengthen the statistical analysis of the data in SI Figures 11 and 12 (now SI Figures 12 and 13). In response, we now analyze the F1, precision, and recall scores for the linker prediction more thoroughly (SI Figure 12). We find that F1, precision, and recall drop below 0.5 for noise levels of ~10 degrees (orientation) and 15-20 Å (translation). These thresholds give us an idea of the required localization precision for reliable linker predictions. We added the following text to SI Figure 12 caption:

We added the results of this analysis to the caption of SI Fig. 12:
"We find that F1, precision, and recall drop below 0.5 on average for noise levels of ~10 degrees (orientation) and 15-20 Å (translation). These thresholds define the required localization precision for meaningful linker prediction using the elastic model."

In SI Figure 13 (formerly 12) we now state that
"The F1, precision, and recall scores stay above 0.5 on average even with ~1/3 of the nucleosomes removed."

We also considered performing paired t-tests on the score distributions for different noise levels (SI Fig. 12) and for different fractions of nucleosomes dropped (SI Fig. 13). However, with the F1, precision, and recall scores and their distributions already being the result of extensive statistical analysis, we concluded that this is not what may have been asked for. In other words, any increase in positional or orientational noise, and any drop in the number of resolved nucleosomes will lead to a significant decrease in performance, given a large enough sample; however, the question is not about significance of the effect (which is evident) but about the magnitude of the effect. That is, based on the results for our test system: at which levels of noise does the performance drop below a reasonable threshold. We therefore concentrated our efforts on this point.

Terminology clarification: word "tracing"

"Reply: We understand the reviewer's point. Although tracing is defined as 'to follow an already defined path', which we think is what we do, our algorithm does not take into account the actually observed electron optical density between nucleosomes, e.g. by segmentation.
We want to stress that we very often have used the term 'predicted linkers' in the manuscript that makes the predictive character of our algorithm perfectly transparent. We nevertheless carefully reconsidered and adjusted our phrasing throughout. We could not entirely eliminate the word trace, because it is also used in the methods part for patches of chromatin (not the linkers), where we think it is appropriate because it indeed refers to connections between confidently identified elements (the nucleosomes). Here, we added this statement:
"We use the verb 'trace' here to describe the connection of nucleosomes into patches of chromatin by interpolating the DNA linkers in-between them."

The authors' interpretation of the term "tracing" appears to correspond to "prediction of the DNA linker" or "interpolation." Moreover, their explanatory statement clarifying this usage is provided only on page 24 within the Methods section. Presenting this

term without clarification in the abstract and introduction is potentially misleading for a general audience, as it can be understood as implying direct detection of cryo-EM density for all reported linkers. For the sake of precision and scientific rigor, the term “tracing” should be replaced in the abstract with “physics-based prediction” or “interpolation,” and the explanatory statement should be introduced before its first use in the main text. Such a change would not diminish the impact of the study but would instead enhance its clarity and accuracy.

We have revised the respective sentence in the abstract as follows:

> *p. 2: “By predicting the paths of linker DNA, we identify oligo-nucleosome arrangements and uncover higher-order chromatin structures in situ, including a 37-nm wide, elongated but non-fibrous arrangement.”*

In the introduction we do not refer to term “tracing”, rather assignment.

> *p. 4: “We resolve the chromatosome to secondary structure in situ; we predict DNA linkers between individual chromatosomes up to kilobase pair (kbp) genomic fragments; “*

Minor comments:

3. The templates used for the “no H1 included” state classification are not cited. Please indicate the corresponding PDB entry.

For the “no H1 included” state, we used the same template as for the “with H1” state, but removed the density of H1 manually using the ChimeraX eraser function to only leave a core nucleosome density. The “with H1” template was derived from our own data.

We have clarified this in the Methods section as follows:

> *p 21:. “First, template matching with 5 degree global angular sampling in GAPSTOP^{TM} [27,28] was performed on bin2 3D-CTF corrected tomograms enhanced with the cryoTIGER workflow (described above in methods section about nucleosome template matching) with two different nucleosome templates, one with H1 density included, the other without it. To obtain the “no H1 included” template, the density of H1 was manually removed from the “with H1” template described above using the ChimeraX eraser function to only leave a core nucleosome density.”*

We now provide the templates as supporting material with a Zenodo deposition.

4. Supplementary Figure 10: The tomographic slices shown in the left panel are very difficult to interpret. It is nearly impossible to recognize individual nucleosomes, especially for readers outside the cryo-EM field, likely due to excessive denoising. I suggest including corresponding isosurface views to facilitate visual interpretation.

The right-most panel shows the respective isosurface view for each tomographic slice. The green dot that indicates the center of the predicted linker is shown in all three images of a row and can thus be used as a point of orientation when comparing the views. We have revised the respective figure legend to make this clearer:

Supplementary Fig. 11: “From left to right for five predicted linkers, with the center of the predicted linker indicated by a green dot:”

REVIEWERS' COMMENTS

Reviewer #2 (Remarks to the Author):
The authors have addressed most of my comments.

Reviewer #3 (Remarks to the Author):
I am satisfied with the revisions and recommend the manuscript for publication.

We thank both reviewers for the positive assessment of our manuscript.
